# Topical Ocular Drug Delivery: The Impact of Permeation Enhancers

**DOI:** 10.3390/pharmaceutics17040447

**Published:** 2025-03-31

**Authors:** Gonçalo Santos, Esmeralda Delgado, Beatriz Silva, Berta São Braz, Lídia Gonçalves

**Affiliations:** 1 Research Institute for Medicines (iMed.ULisboa), Faculty of Pharmacy, Universidade de Lisboa, 1649-003 Lisboa, Portugal; goncalonunosantos@campus.ul.pt; 2 CIISA—Centro de Investigação Interdisciplinar em Saúde Animal, Faculty of Veterinary Medicine, Universidade de Lisboa, 1300-477 Lisboa, Portugal; esmeralda@fmv.ulisboa.pt (E.D.); brsilva@fmv.ulisboa.pt (B.S.); bsaobraz@fmv.ulisboa.pt (B.S.B.); 3 Associate Laboratory for Animal and Veterinary Sciences (AL4AnimalS), 1300-477 Lisbon, Portugal

**Keywords:** ocular, delivery, permeation, enhancers

## Abstract

Topical ophthalmic drug delivery targeting the posterior segment of the eye has become a key area of interest due to its non-invasive nature, safety, ease of application, patient compliance, and cost-effectiveness. However, achievement of effective drug bioavailability in the posterior ocular segment is a significant challenge due to unique ocular barriers, including precorneal factors and anatomical barriers, like the cornea, the conjunctiva, and the sclera. Successful ocular drug delivery systems require increased precorneal residence time and improved corneal penetration to enhance intraocular bioavailability. A promising strategy to overcome these barriers is incorporating drug penetration enhancers (DPEs) into formulations. These compounds facilitate drug delivery by improving permeability across otherwise impermeable or poorly permeable membranes. At the ocular level, they act through three primary mechanisms: breaking tear film stability by interfering with the mucous layer; disrupting membrane components such as phospholipids and proteins; and loosening epithelial cellular junctions. DPEs offer significant potential to improve bioavailability and therapeutic outcomes, particularly for drugs targeting the posterior segment of the eye. This review is focused on analyzing the current literature regarding the use of penetration enhancers in topical ocular drug delivery, highlighting their mechanisms of action and potential to revolutionize ophthalmic treatments.

## 1. Introduction

According to the World Health Organization (WHO), as of 2019, at least 2.2 billion people worldwide suffer from visual impairment, with nearly 1 billion cases being preventable [1]. As the global population ages, the prevalence of ocular chronic diseases, a major cause of visual impairment, will increase significantly in relevance and magnitude. This highlights the urgent need for improvement in eye health and for investment in research to develop more effective treatments for ocular diseases, recognizing this field as one of the most challenging in medicine.

The successful topical application of drugs to treat intraocular diseases remains a significant challenge in ophthalmology since there are several ocular barriers to overcome. Topical drug delivery is the preferred and most widely used ophthalmic route for the treatment of the anterior ocular segment, essentially due to its non-invasive and easy-to-use nature. Nevertheless, it faces substantial anatomic (cornea, conjunctiva, and sclera) and physiologic (tear dilution, nasolacrimal drainage, reflex blinking, etc.) challenges that negatively affect drug bioavailability in the posterior segment of the eye (vitreous humor, retinal pigmented epithelium, retina, and choroid), where the vast majority of chronic ocular diseases originate and progress [2,3,4,5,6,7]. These limitations often result in less than 5% of the topically administered dose reaching the inner ocular tissues, making enhanced drug penetration an absolute necessity for effective treatment [4].

The cornea is the primary absorption pathway of topically applied drugs and the main route for the posterior ocular segment. However, it is considered to be one of the most specialized barriers in the organism and the predominant ocular anatomical barrier affecting both hydrophilic and hydrophobic drug delivery [8]. In contrast, the conjunctiva has a more porous and permeable epithelium but, as an extremely well-vascularized membrane, contributes substantially to systemic drug absorption, reducing intraocular drug bioavailability and leading to side effects [9,10].

DPEs play a crucial role in overcoming these challenges due to their ability to enhance drug delivery across otherwise impermeable or poorly permeable membranes [11]. DPEs improve drug permeation across the cornea by destabilizing the tear film, disrupting membrane components, and loosening cellular junctions [12]. Ideal penetration enhancers should be locally and systemically safe, effective at low concentrations, chemically and pharmacologically inert, compatible with a wide range of drugs and pharmaceutical excipients, and the effect should be reversible, leading to full recovery of the normal membrane barrier function [13].

While earlier reviews such as Kaur and Smitha [13] laid the groundwork by introducing bioadhesives and penetration enhancers, and Morrison and Khutoryanskiy [10] offered a broader overview of ocular drug delivery strategies with a focus on various delivery systems like implants and inserts, later efforts like Moiseev et al. [11] and Thareja et al. [14] focused narrowly on specific enhancer classes, non-chemical penetration enhancement methods, or posterior segment applications but left significant gaps in clinical translation and mechanistic integration. In contrast, this review focuses on topical ocular drug delivery, with permeation enhancers as the primary topic, providing the most comprehensive, up-to-date, and application-oriented review to date, uniquely combining a deep mechanistic analysis of DPEs with a critical appraisal of their translational potential for both anterior and posterior ocular segments. By uniting cutting-edge scientific developments with practical insights into formulation strategies and incorporating emerging biocompatible carriers, this work sets a new benchmark in the field, addressing unmet challenges and paving the way for next-generation ophthalmic therapies.

This review stands at the forefront of ocular pharmacology, offering critical insights into the transformative role of permeation enhancers in topical drug delivery. By unveiling breakthroughs in enhancing drug bioavailability and transcorneal permeability, this work illuminates new pathways for targeting both the anterior and posterior ocular segments. With the potential to improve therapeutic outcomes, reduce side effects, and elevate patient compliance, the findings presented herein mark a pivotal step toward reshaping the future of ophthalmic treatments.

## 2. Ocular Anatomy and Physiology

### 2.1. Ocular Globe

**Figure 1:**
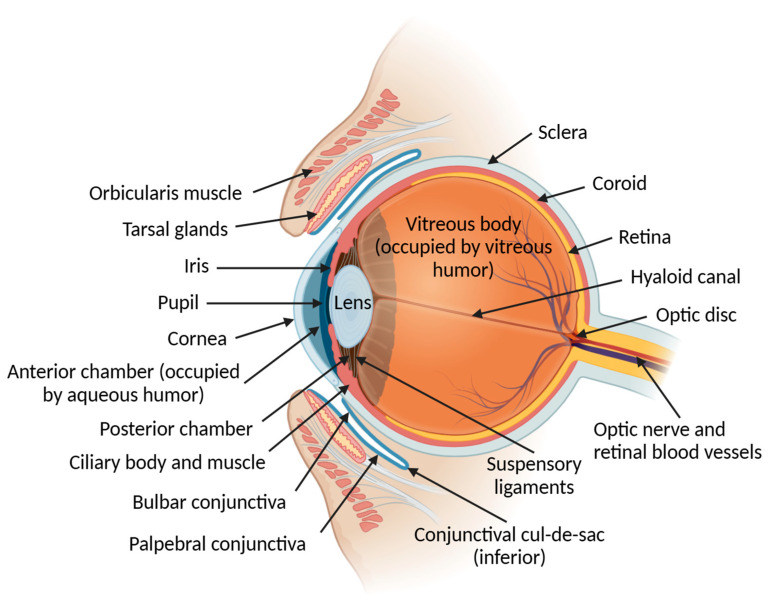
Sagittal section of the eye and eyelids.

The eye is a highly specialized organ with an individual anatomy and physiology that enables the capture of visual stimuli and their transmission via the optic nerve to the visual cortex of the brain for further processing [5]. Successful treatment of various ocular conditions remains a significant challenge since there are multiple anatomical and physicochemical ocular barriers that are intrinsic to this organ structure and function. Figure 1 demonstrates a sagittal section that describes the various components of the ocular and eyelid anatomy.

This initial review focuses on the precorneal tear film and the main anatomical barriers to topical drug administration, from the outer to the inner layers of the ocular medium (Table 1).

### 2.2. Precorneal Tear Film (PTF)

The PTF is the interface through which the ocular surface meets the environment, covering the exposed cornea and conjunctiva. Its stability is crucial for the maintenance of unclouded vision as its primary role is to provide an optically high-quality refracting surface to the cornea, enabling light to correctly move into the visual system [24]. Furthermore, it acts as a lubricant, protecting and moisturizing the cornea and conjunctiva. It is the primary oxygen source to the cornea, it contains enzymes with microbicidal properties, it provides an access route for leukocytes, and it is involved in removing foreign matter or cell debris following normal desquamation [45].

### 2.3. Cornea

The cornea is considered to be the dominant anatomical barrier for drugs applied to the ocular surface [7]. It is an avascular, highly transparent, elliptical-shaped structure whose diameter and thickness vary between species, breeds, and even individually [26,46]. The outer surface contacts with the tear film and is smooth and convex, while the inner surface is concave and corresponds to the anterior boundary of the anterior chamber [47]. Together with the sclera, it forms the fibrous tunic of the globe, which serves to support and protect intraocular contents [48]. Along with the tear film, it provides an appropriate anterior refractive surface [32].

Upon microscopic examination, the cornea is composed of four layers in companion animals. From the outermost part to the innermost part, we have the corneal epithelium and basement membrane, the corneal stroma, Desmecet’s membrane (the basement membrane of the corneal endothelium), and the corneal endothelium [48]. In humans and in most primates, there is a fifth layer called Bowman’s membrane or layer, which lies immediately posterior to the epithelial basement membrane and anterior to the stroma. It has also been found in chicken, deer, giraffe, antelope, guinea pig, and several other species [49].

### 2.4. Conjunctiva

**Figure 2:**
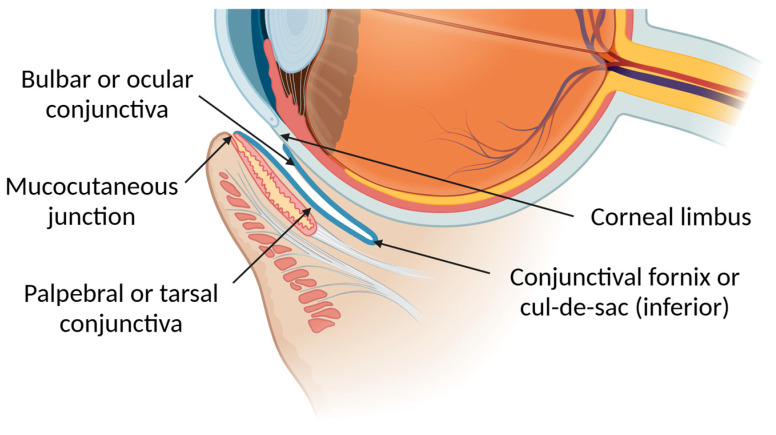
Sagittal section of the eye and eyelid, demonstrating the various regions of the conjunctiva.

The conjunctiva is the most exposed mucous membrane of all and covers about 80% of the ocular surface [50]. It is a mobile, densely vascularized (blood and lymphatic vessels), thin, and translucent mucous membrane that covers the interior surface of the eyelids, curving onto the anterior surface of the eyeball and extending towards the cornea. It is continuous with the skin at the mucocutaneous junction of the eyelid margin and with the corneal epithelium at the corneal limbus [39]. Despite being a continuous membrane, conjunctiva is divided into three main regions: the palpebral or tarsal conjunctiva, the bulbar or ocular conjunctiva, and the conjunctival fornices or cul-de-sacs (superior and inferior) formed by the union of the palpebral and bulbar areas [51]. This division can be more easily observed in Figure 2.

### 2.5. Sclera

The sclera comprises most of the fibrous tunic of the eye. At the posterior segment of the ocular globe, the sclera fuses with the dural sheath of the optic nerve. In the anterior segment, it merges with the peripheral cornea and the bulbar conjunctiva, forming a transition zone, called corneo-scleral junction or corneal limbus [52].

### 2.6. Nasolacrimal Drainage System

**Figure 3:**
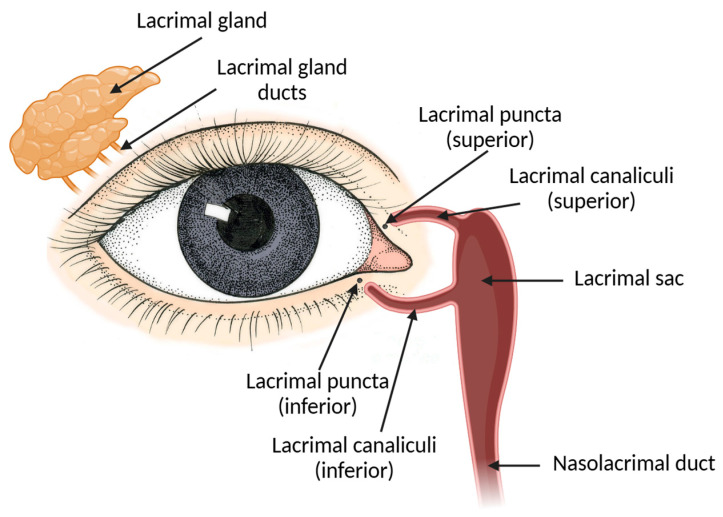
Nasolacrimal drainage system.

The nasolacrimal drainage system (Figure 3) provides the correct balance between inflow and outflow of tears to the lacrimal sac to guarantee the hydration of the ocular surface and the normal refraction of light in the vision process [53]. It comprises secretory, distributive, and collection parts [54].

Under normal conditions, the conjunctival cul-de-sac of humans has a standard volume of 7 to 10 µL, expanding to a maximum of 30 µL without overflow occurring [55,56]. After secretion, tears spread across the ocular surface by the eyelids during blinking. The drainage occurs mainly through the nasolacrimal duct system that begins at the medial inferior and superior lacrimal puncta. The lacrimal puncta open into the superior and inferior canaliculi, which run through the orbicularis oculi muscle and connect to each other before entering the lacrimal sac that lies within the lacrimal fossa in the lacrimal bone. The lacrimal sac drains to the nasolacrimal duct that ends in the nasal punctum, usually located in the ventral lateral nasal meatus [57,58]. The blinking movement has a pumping action in the canaliculi, further promoting tear drainage [59]. The excess tears spill onto the face, which is known as epiphora [60].

## 3. Ocular Pharmacokinetics After Topical Administration

Topical administration is the most employed route of drug administration for the treatment of anterior ocular segment disorders due to its safe and non-invasive nature, rapid onset of action, convenience, and patient compliance [4]. Indeed, formulation for topical administration accounts for nearly 90% of the currently advertised and marketed ocular drug delivery systems [3]. Nevertheless, targeting the posterior segment through this route is an extremely challenging task due to the numerous precorneal factors and ocular barriers that drastically reduce drug bioavailability in inner ocular tissues, such as the vitreous humor, retina, and choroid [2].

An eye drop volume ranges from 25 up to 70 µL, averaging 40 to 50 µL, as the human conjunctival cul-de-sac can only accommodate 30 µL and already has an ordinary volume of 7 to 10 µL. Right after the application of the drop, there is an immediate loss of a significant portion of the solution (20 to 30 µL) on the skin [56,61]. Reflex blinking, tear production, and nasolacrimal drainage further dilute the drug in the first 15 to 30 s, reducing its concentration in the lacrimal fluid by 2 to 4 times [55,60,61,62]. Furthermore, the rapid drainage of about 80% of the administered volume, that is not spilled on the skin, through the nasolacrimal duct allows systemic absorption of the drug [63].

The main factors that influence the drainage rate include the eye drop volume and the viscosity, pH, and tonicity of the formulation [64]. Reducing the drop size has been proven to minimize drug loss, systemic absorption, incidence of systemic side effects, and treatment cost while maintaining or improving ocular bioavailability [63,65,66]. This depends on the design of the dropper tip and bottle, physicochemical properties of the solution, and the patient’s handling of the dropper and administration skills [67]. In general, it is advisable to use a higher drug concentration in a small instillation volume, especially if systemic toxicity is a problem. The increase in viscosity can extend the residence time of the drug in the conjunctival sac and enhance its corneal absorption but may cause discomfort, irritation, and vision blurring [3,63,67]. The physiological pH of human tear film ranges from 6.5 to 7.6 (7.0 on average) [68], but, in cattle, horse, and dog, it has been stated as more alkaline, approximately 8.32, 7.84, and 8.05 on average, respectively [69]. The instillation of acidic or alkaline solutions may produce a burning sensation, which stimulates lacrimation and increases tear turnover, resulting in a loss of drug [61,64]. The tonicity of eye drops should be close to natural tears. Both hypotonic and hypertonic solutions produce discomfort [70]. Additionally, hypertonic solutions promote osmotic movement of water across the membranes of the ocular tissues to the tear film, which increases its volume, dilutes the drug, and promotes nasolacrimal drainage [71]. Furthermore, tear film proteins such as lysozyme, lipocalin, lactoferrin, albumin, transferrin, and immunoglobulins can bind to drugs, reducing their free drug concentration present in the PTF and, consequently, their bioavailability at the targeted site [64,72]. Optimizing these parameters is essential for enhancing the efficacy of topical ophthalmic drug delivery.

## 4. Assessment of Drug Permeability

The development of effective ocular drug delivery systems necessitates a robust understanding of drug permeation across ocular tissues, which serve as both anatomical and functional barriers to therapeutic agents. The complexity of ocular pharmacokinetics related to topical drug delivery is dictated by a multilayered defense system, including the precorneal tear film, corneal epithelium, conjunctiva, and sclera, all of which are essential for maintaining ocular homeostasis but collectively limit drug absorption into both anterior and posterior ocular segments. Effective ocular drug delivery hinges on the ability of active pharmaceutical agents to traverse these barriers, making permeability evaluation a critical component in formulation development and optimization.

The accurate assessment of drug permeability involves a multidisciplinary approach encompassing in vitro, ex vivo, and in vivo methodologies. Each approach provides distinct insights into the drug transport mechanisms, enabling the identification of promising candidates and formulation strategies prior to clinical application.

In vitro cell-based models offer a controlled environment to evaluate drug permeation, cytotoxicity, and tight junction integrity, while artificial membrane systems allow rapid screening of passive diffusion characteristics. Ex vivo studies using excised ocular tissues simulate real-tissue barrier properties more accurately, and in vivo animal models provide valuable pharmacokinetic and pharmacodynamic data that are especially relevant to clinical translation.

### 4.1. In Vitro Models

#### 4.1.1. Cell Culture Models

In vitro cell culture models serve as indispensable tools for the preliminary evaluation of ocular drug delivery systems, offering valuable mechanistic insights into drug permeability, cytotoxicity, and epithelial interactions. By mimicking key structural and functional attributes of ocular epithelial barriers, these models enable the investigation of drug transport dynamics, tight junction modulation, and formulation-induced cellular responses prior to ex vivo or in vivo validation. They are favored for their cost-effectiveness, reproducibility, and ethical advantage in reducing animal experimentation. Nonetheless, these systems present inherent limitations as they fail to fully replicate the physiological complexity of the ocular surface, often leading to overestimations of permeability and poor in vitro–in vivo correlations, particularly for formulations influenced by precorneal mechanisms. Widely used models include human corneal epithelial cells [73,74,75,76,77,78], cultured rabbit corneal epithelial cells [79,80,81], and conjunctival epithelial cells [82,83].

##### Apparent Permeability Coefficient (Papp)

Papp is a pivotal parameter in ocular drug delivery research, quantifying the rate at which a drug permeates through a biological ocular membrane, such as the cornea, conjunctiva, or sclera. It serves as an indicator of a compound’s permeability characteristics, aiding in the prediction of its absorption and therapeutic efficacy within ocular environments.

Papp is defined as the rate of drug permeation across a biological membrane normalized by the membrane’s surface area and the initial drug concentration, which is supposed to be zero [84]. Mathematically, it is expressed as

$$Papp=\frac{J}{CD\times S}$$

where *J* represents the transmembrane fluxes of the investigated substance from the donor to the receptor solution, which is usually determined from a scatter diagram as the slope of the time–concentration regression line at the time interval is predetermined. *CD* is the initial concentration of the drug in the donor solution, and *S* represents the eye membrane surface area [85].

##### Transepithelial Electrical Resistance (TEER)

Transepithelial electrical resistance (TEER) is an extensively utilized, non-invasive gold standard technique for evaluating the integrity and permeability of epithelial cell monolayers in vitro. By quantifying the electrical resistance across these cellular layers, TEER enables a precise assessment of tight junction integrity, an essential determinant of barrier function [86,87]. This method provides a non-destructive means to assess paracellular barrier dynamics, facilitating the investigation of disruption or restoration mechanisms in response to drug delivery systems or permeation enhancers, serving as a crucial tool in biomedical research for the development and evaluation of therapeutic strategies targeting barrier-related functions [86]. The electrical resistance of a cellular monolayer is measured in ohms. Briefly, TEER measurements involve placing electrodes on either side of a cell monolayer cultured on a permeable membrane that defines a partition for apical and basolateral compartments. An alternating current voltage signal is applied, and the resulting voltage drop is measured to calculate the electrical resistance using Ohm’s law [86]. Higher TEER values indicate strong tight junctions and low permeability, while lower TEER values suggest a compromised barrier, indicating increased permeability [87].

### 4.2. Ex Vivo Models

Excised tissue models are pivotal in ocular drug delivery research, providing a relevant platform to evaluate drug retention, permeation, and absorption with high precision under conditions that closely mimic physiological environments.

**Figure 4:**
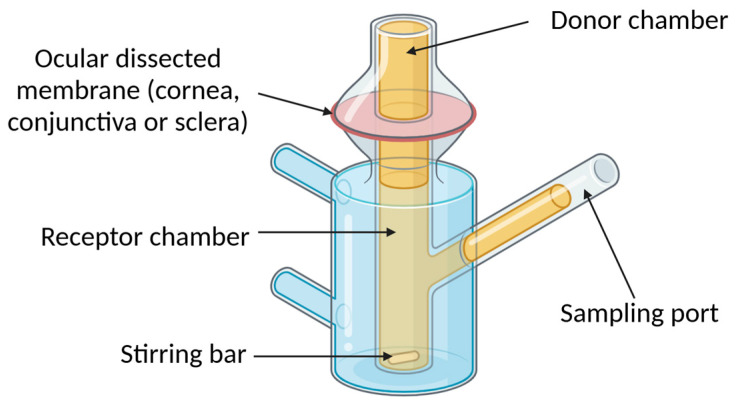
Franz-type diffusion cell.

Utilizing freshly excised corneal tissues from various animal species (bovine, porcine, rabbit, goat, sheep, and buffalo), these models preserve key structural components, including the epithelium, stroma, and endothelium, ensuring a more representative approximation of ocular physiology than traditional in vitro models [85,88,89,90,91,92]. Typically, the excised membranes are mounted onto diffusion cells (frequently Franz-diffusion-type cells), allowing researchers to meticulously investigate drug transport across them (Figure 4). Their controlled experimental conditions enhance reproducibility while reducing reliance on live animal testing, making them a cost-effective alternative for preliminary drug screening and formulation optimization. However, their limited post-excision viability restricts the timeframe for experimentation and necessitates prompt and careful handling to maintain tissue integrity. Furthermore, interspecies differences in corneal permeability and the inability of these models to account for dynamic ocular factors, such as tear film dynamics, blinking, and systemic clearance, which play crucial roles in the distribution and clearance of ophthalmic drugs in vivo, can affect the translational relevance of the findings [74]. Despite these limitations, continuous advancements, such as the incorporation of tear replenishment systems, are enhancing their predictive capabilities [93]. As these models evolve, they remain indispensable in bridging the gap between in vitro studies and in vivo applications, driving innovation in ophthalmic drug development.

### 4.3. In Vivo Models

Animal models are essential tools in ocular drug delivery research, providing a biologically relevant and dynamic environment that closely mimics human ocular physiology. While many key aspects of ocular anatomy and function are conserved across vertebrates, each species exhibits distinct ocular characteristics that must be carefully considered when translating preclinical findings to human applications [94]. Variations in anatomical structures, such as corneal thickness, scleral permeability, aqueous humor dynamics, and retinal composition, can significantly influence drug interactions within the eye. These interspecies variations affect drug distribution, retention, and clearance, whether administered topically, intravitreally, or systemically. Among these models, rabbits are considered the gold standard because they are easy to manipulate and share, at ocular level, anatomical, biomechanical, and biochemical characteristics with humans [95,96]. These animals are widely used to study diseases such as glaucoma [97,98] and corneal neovascularization [99,100]. Ocular pharmacokinetics is commonly assessed by sampling aqueous humor, vitreous humor, or ocular tissues, including the cornea, conjunctiva, sclera, and retina, to determine drug distribution, retention, and clearance rates. By offering a balance between physiological relevance and practical feasibility, animal models remain indispensable in advancing ocular therapeutics while guiding the development of more effective and targeted drug delivery strategies.

Despite their advantages, the use of animal models in ocular drug research presents certain challenges. The limited aqueous and vitreous volumes in commonly used laboratory species complicates dosing methodologies, often requiring highly sophisticated techniques. As a result, high-performance liquid chromatography (HPLC) coupled with mass spectrometry is frequently employed for drug quantification, but the high cost and limited availability of such advanced analytical methods can pose significant constraints.

Future research should focus on refining analytical techniques and exploring alternative models, such as organotypic cultures and computational simulations, to complement in vivo studies. By integrating emerging technologies with traditional models, researchers can enhance the predictive accuracy of preclinical studies and accelerate the development of more effective and targeted ocular drug delivery systems.

## 5. Enhancement of Ocular Membrane Permeability: A Strategy to Improve Topical Ocular Drug Delivery

Recently, the ophthalmic pharmaceutical industry has experienced a seismic shift, with increasing emphasis on the treatment of retinal diseases [101]. Traditionally, topical ocular drug delivery has been predominantly utilized to address conditions affecting the anterior segment of the eye. However, this route offers negligible drug bioavailability in the vitreous chamber given the numerous delivery challenges, such as precorneal factors and complex ocular barriers [6].

The primordial objective in developing ophthalmic formulations is to achieve and sustain an optimal concentration of the active substance at the targeted site for an adequate length of time [102]. To overcome the limitations of topical ocular drug delivery, two different strategies have emerged: increasing corneal residence time (using viscosity enhancers, mucoadhesive agents, or in situ gels) or enhancing corneal permeability (with the application of, for example, penetration enhancers, nanoparticles, or liposomes) [103].

This review article highlights the potential of permeation enhancers as a promising approach for improving the bioavailability of topically applied drugs aiming at the posterior segment of the eye, offering new avenues to overcome the existing challenges in this field.

### 5.1. Cyclodextrins (CDs)

CDs are a family of naturally occurring water-soluble cyclic oligosaccharides with a truncated cone shape that have been increasingly used in ocular drug formulations due to their unique molecular properties [11,104,105].

CDs feature an apolar central cavity that is lined with skeletal carbons and ethereal oxygens, providing a lipophilic microenvironment capable of forming non-covalent inclusion complexes with drug molecules and a hydrophilic outer surface coated with hydroxyl groups that form hydrogen bonds with water molecules, thus enhancing water solubility [106,107,108,109,110]. These complexes improve aqueous solubility, stability, dissolution rate, and bioavailability of poorly soluble drugs while reducing local irritation after ocular application of potentially irritating drugs [106,107,111].

The most common natural CDs are α-CD, β-CD, and γ-CD, which consist of six, seven, and eight α-(1,4)-linked glucopyranose subunits, respectively [112]. βCD has the lowest water solubility among the parent CDs, and γ-CD is more soluble in water than both α-CD and β-CD [113]. The most frequently used CD derivatives in ophthalmology include hydroxypropyl (HP) derivatives of β- and γ-cyclodextrins, randomly methylated (RM) β-cyclodextrin, and sulfobutylether (SBE) β-cyclodextrin [114]. SBE-β-CD and HP-β-CD present better pharmaceutical solubilization properties when compared to RM-β-CD and to the parent βCD [108,115]. The main marketed ophthalmic formulations including CDs are listed in Table 2.

The inclusion complex formation is size-dependent, with α-CD (cavity size 4.7–5.3 Å) presenting a relatively small cavity, unable to form stable inclusion complexes with many drug molecules, and with β-CD (cavity size 6.0–6.5 Å) offering an optimal balance for accommodating a wide range of drugs. When the cavity is excessively large, as with other CDs, drug molecules may bind loosely, diminishing their efficacy.

CDs are unable to permeate across intact lipophilic biological membranes, such as the corneal or conjunctival epithelium, mostly due to their chemical structure and molecular weight [108,116]. Nevertheless, drug molecules can dissociate from the complex and become free to interact with and permeate these membranes [117]. This is advantageous in ocular drug delivery as CDs facilitate drug release near the ocular surface, enhancing their precorneal retention and corneal permeation [107]. In fact, the bulk of latest studies use CDs and their derivatives in ophthalmic formulations for topical use, mainly targeting the anterior segment of the eye (Table 3), especially for the treatment of keratitis and anterior uveitis [111,118,119,120,121,122], corneal ulcer healing [123], fungal keratitis [115,124,125,126], allergic and bacterial conjunctivitis [127,128], and dry eye disease [129,130,131,132]. Although there is some evidence that CDs provide efficient drug delivery to the posterior eye segment [117,133,134,135,136,137,138], few recent studies explore this field, often resorting to formulations that favor CD corneal permeation, such as liposome nanocomposites [139,140], nanoparticles [141,142], inserts, or micelles [143], for this purpose.

### 5.2. Chelating Agents

Chelating agents, particularly calcium chelators, have been explored in ocular drug delivery to enhance drug penetration across the corneal epithelium, which, due to its lipophilicity and junctional complexes, acts as a major barrier for the transcellular and paracellular permeation of hydrophilic molecules.

Chelating agents act as preservative agents as well, with strong antimicrobial and antibiofilm activities, by chelating especially magnesium, iron, and calcium cations, which interferes with the structural integrity of the microbial cell walls and membranes of Gram-negative and -positive bacteria, yeasts, amoeba, and fungi, leading to their death and/or growth inhibition, and strengthens other antimicrobials [145,146]. Calcium plays a critical role in maintaining cellular junctions, particularly tight junctions (TJs) and adherent junctions (AJs), which are essential for epithelial integrity. Calcium chelating agents destabilize these junctions that rely on calcium-dependent cadherin proteins for adhesion, disrupting epithelial architecture [147,148,149,150,151,152]. E-cadherin, a prominent member of the cadherin family, mediates calcium-dependent intercellular adhesion through its extracellular cadherin repeats, whose conformation is controlled by the interaction with calcium ions, switching their adhesive function [153,154,155]. Extracellular calcium depletion results in disengagement of cadherins, leading to loss of cellular adhesion and TJ disruption via actomyosin contraction of the peri-junctional actomyosin ring (PAMR), widening the intercellular spaces and thereby increasing epithelial permeability [156].

Calcium chelators such as ethylenediamine-N, N, N′, N′-tetraacetic acid (EDTA), ethylene glycol-bis(beta-aminoethyl)-N, N, N’, N’-tetraacetic acid (EGTA), and ethylenediamine-N, N’-disuccinic acid (EDDS) have demonstrated the ability to modulate epithelial junction permeability and enhance drug permeation across the corneal epithelium. According to Morrison et al., EGTA and EDTA exhibit superior efficacy in reducing corneal transepithelial electrical resistance and enhancing corneal permeability compared to EDDS [157]. The two studies found on EGTA are in favor of its use as an ocular permeation enhancer, but further investigation is required [157,158]. The most recent literature approaching EDTA is controversial. Some authors [92,157,159,160] describe a significant ocular-permeation-enhancing effect, while others [161,162,163,164,165] report that this effect is not significant for both hydrophilic and hydrophobic drugs. Some studies demonstrate that the combination of EDTA with benzalkonium chloride or boric acid improves the ocular permeation when compared to their application alone [92,160,161,162,163]. Controversially, other studies report no effect or even a significant reduction in the corneal permeation of drugs [159,166].

However, the use of chelating agents must be approached with caution due to potential toxicity that tends to be dose-dependent and noticeable at concentrations as low as 0.01% [167]. EDTA and its derivatives are known to cause morphological, sometimes irreversible, alterations of the corneal epithelium. Furthermore, it can penetrate through the cornea, affecting the corneal endothelium, and it can be accumulated in the anterior segment tissues like the iris and the ciliary body, potentially affecting endothelial cells and uveal tract capillaries [168,169]. To mitigate these potential adverse effects, these compounds could be combined with other permeation enhancers, mucoadhesive formulations, or ocular delivery systems, improving their precorneal residence time and sustained delivery. Ongoing research is essential to develop safe and effective formulations that leverage the benefits of chelating agents while minimizing potential adverse effects.

### 5.3. Crown Ethers (CEs)

CEs are a class of synthetic macrocyclic oligomers of ethylene oxide consisting of linked ether groups and a distinctive crown-like molecular structure [11]. They are typically composed of 3 to 20 oxygen atoms separated by two or more carbon atoms. Their names reflect the number and type of atoms in the polyether ring, divided by the letter “C” or the class name “Crown”, and the number of oxygen atoms in the polyether ring [170]. Among the most effective members, 12-Crown-4, 15-Crown-5, and 18-Crown-6 have been extensively studied for their biological applications, particularly in ocular drug delivery [171].

These flexible molecules adapt to their environment, exhibiting amphiphilic properties that allow them to dissolve in both aqueous and lipidic solvents [171,172]. Their structure features a hydrophobic molecular ring structure encircling a hydrophilic cavity, enabling selective binding and forming stable guest–host complexes with various metal ions (e.g., K^+^, Na^+^, Ca^2+^, and Mg^2+^) and organic species by non-covalent interactions. This ability facilitates the transport of these bound entities across non-aqueous solvents and lipid membranes, the binding efficiency being dependent on their cavity size [173,174,175,176].

CEs are particularly valuable in enhancing drug delivery through ocular tissues. They can significantly improve the extraction of calcium ions from bovine corneal epithelium, loosening calcium-dependent tight junctions, thereby enhancing corneal drug permeation [171]. Additionally, their amphiphilicity and ionophoric properties are ideal for promoting better drug permeation in drug delivery systems, where the formulation must interact with both aqueous and lipophilic phases and cross cellular membranes [11,177]. By improving drug solubility, biocompatibility, and permeation across corneal epithelium, CEs hold promise for better therapeutic outcomes [171,178]. The main studies on CEs as ocular permeation enhancers are listed in Table 4.

Despite their low toxicity and vast potential, CEs exhibit limited in vivo retention on ocular surfaces due to the dynamic nature of these tissues, and, therefore, they have not been significantly studied in recent years. The combination between CE and mucoadhesive molecules could enhance precorneal retention and, consequently, drug permeation, making this a promising area for further research [171].

### 5.4. Chitosan (CH)

Chitosan [poly(1,4-β-D-glucopyranosamine)] is a biodegradable, biocompatible, and cationic linear polysaccharide obtained through the alkaline deacetylation of chitin, a naturally occurring polymer predominantly found in crustacean exoskeletons, insect cuticles, and fungal cell walls [54,179]. This versatile biopolymer has garnered significant attention in biomedical, pharmaceutical, and nanotechnological applications, particularly in ocular drug delivery, due to its unique physicochemical and biological properties, including low toxicity, mucoadhesiveness, antimicrobial activity, and its ability to modulate drug permeability [180]. Furthermore, CH is well tolerated by biological tissues, eliciting minimal inflammatory or allergic responses, making it an ideal candidate for sustained ophthalmic therapies [181,182,183].

CH’s efficacy as a mucoadhesive cationic polymer in enhancing topical drug penetration is largely attributed to its ability to interact electrostatically with mucins, the primary glycoproteins of the tear film. This interaction, driven by hydrogen bonding and ionic attraction between the protonated amino groups of CH and the negatively charged sialic acid residues of mucins, prolongs drug retention at the ocular surface [54]. Additionally, its cationic nature allows it to interact with negatively charged biological membranes [184]. By minimizing premature drug clearance due to blinking and tear turnover, CH and its derivatives, like thiolate, trimethyl, or carboxymethyl CH, extend precorneal residence time, enhancing drug bioavailability and potentially reducing the frequency of administration [185,186,187,188,189,190]. These attributes are particularly beneficial in managing chronic ophthalmic conditions requiring sustained drug release, thereby improving patient compliance and therapeutic outcomes. They have been exploited in the development of drug-loaded polymeric nanoparticles, aiming to enhance drug retention at mucosal sites and improve ocular permeation and therapeutic efficacy. Combining CH with polyanionic polymers like hyaluronic acid (HA) [186,191,192,193,194], baicalin methoxy poly(ethylene glycol)-poly(d,l-lactic-*co*-glycolic acid) [195], sodium alginate [91,185], and chondroitin sulfate [196] has been explored to optimize these delivery systems, showing significant potential to prolong the residence time of the ophthalmic formulations in the conjunctival sac.

Beyond its mucoadhesive properties, CH exhibits intrinsic antimicrobial activity, which is highly advantageous in ocular therapeutics. Given that conditions such as keratoconjunctivitis sicca (KCS) are often associated with reduced tear secretion and compromised ocular defenses, CH’s antimicrobial function helps to mitigate secondary infections by compensating for diminished levels of lysozyme and lactoferrin in the tear film [54].

Mechanistically, CH enhances ocular drug penetration through multiple pathways. It transiently and reversibly disrupts epithelial tight junctions by modulating cytoskeletal components, leading to increased paracellular permeability [197]. Specifically, CH influences the redistribution of ZO-1 proteins and disrupts the actin cytoskeleton, reducing transepithelial electrical resistance and facilitating the transport of hydrophilic drugs [197,198,199,200]. Additionally, CH interacts with the lipid bilayer of epithelial cells, transiently increasing membrane fluidity, thereby promoting transcellular drug diffusion without causing significant cytotoxicity or permanent alterations to cellular integrity [201].

Recent studies highlight CH-based formulations as promising vehicles for enhancing drug permeation and therapeutic efficacy while minimizing drug concentration and dosing frequency. These advancements have significant implications for treating various ocular diseases, including fungal keratitis [91,202,203,204,205,206,207,208], Acanthamoeba keratitis [209], bacterial keratitis [205,210,211,212,213,214,215,216,217,218,219], glaucoma [187,191,220,221,222,223,224,225,226], corneal burns [227], diabetic retinopathy [228], or ocular inflammatory conditions [229,230,231,232,233,234,235,236]. CH has been extensively investigated in the development of innovative drug delivery systems, including nanoparticles [20,21,22,23,24,25,26,27,28,29,30,31,32,33,34,35], gels [36,37,38,39], liposomes [226], nanomicelles [225,230], mixed micelles [234], nanostructured lipid carriers [228], emulsions [237,238], nanoemulsions, microparticles [233], nanospheres [218], inserts [239,240,241,242,243], phytocubosomes [221], CH conjugates [206,207], and contact lenses [244,245,246,247,248,249]. These formulations optimize drug bioavailability, ensuring controlled and sustained release and thereby revolutionizing ophthalmic drug delivery and improving patient outcomes.

Recently, Silva et al. conducted a groundbreaking study in which they developed CH-hyaluronic acid-epoetin beta (CS/HA-EPOβ) nanoparticles for topical ocular administration in a rat model of glaucoma [191]. This innovative nanoformulation demonstrated a remarkable ability to enhance the retinal penetration of EPOβ, leading to a significant acceleration in retinal recovery and further substantiating its neuroprotective efficacy. These findings underscore the transformative role of CH-based nanotechnology in ophthalmology, offering a promising non-invasive strategy for targeted retinal drug delivery. The use of CS/HA-EPOβ nanoparticles not only mitigates the risks associated with conventional invasive administration routes but also improves drug bioavailability, enhances therapeutic efficacy, and fosters long-term patient adherence—critical factors in the management of chronic ocular neurodegenerative diseases such as glaucoma. This study paves the way for the development of advanced nanomedicine-based interventions, emphasizing the potential of polymeric nanoparticles in optimizing ocular drug delivery and neuroprotection.

Given its multifaceted role in enhancing ocular drug penetration, CH continues to be a focal point of research in ophthalmology, with ongoing advancements reinforcing its potential in non-invasive, effective, and patient-friendly ocular therapies.

### 5.5. Surface-Active Agents (SAAs)

SAAs are amphiphilic molecules composed of a polar head and a nonpolar tail. At low concentrations, these compounds accumulate onto surfaces or interfaces between aqueous and non-aqueous media, reducing the interfacial free energy. When the concentration reaches a threshold known as critical micelle concentration (CMC), SAAs self-assemble into micelles, a process influenced by the SAA structure and the pH, the ionic strength, and the temperature of the solution. Longer nonpolar tails are characterized by higher hydrophobicity, resulting in a lower CMC [11,250]. When topically applied, they possess the ability to disrupt tear film, the mucin, and the integrity of corneal and conjunctival epithelial cell membranes [11].

SAAs are classified by the charge of their polar head into four main groups: non-ionic (no charge), cationic (positive charge), anionic (negative charge), and zwitterionic/amphoteric (dipolar, with charge dependent on the environment). For ocular drug delivery, non-ionic SAAs are preferred due to their lower toxicity, reduced CMC, better biocompatibility, enhanced drug solubility and formulation stability, and versatility, making them ideal for novel delivery systems. In contrast, ionic SAAs (cationic, anionic, and amphoteric) are more polar and associated with higher toxicity, which limit their applicability in sensitive ocular environments [250].

#### 5.5.1. Non-Ionic SAAs

##### Polyoxyethylene Alkyl Derivatives (PADs)

Polyoxyethylene alkyl ethers are non-ionic, biodegradable surfactants with low toxicity, synthesized by polyethoxylating linear fatty alcohols with ethylene oxide [251]. The degree of ethoxylation can be tailored, producing a diverse range of compounds, including polyoxyethylene glycol ethers of N-alcohols (e.g., lauryl, myristyl, cetyl, and stearyl) [252]. Being widely utilized in industrial, household, and pharmaceutical products, these surfactants serve as emulsifying, solubilizing, dispersing, and wetting agents [251].

Structurally, the hydrophilic part consists of a polyoxyethylene moiety, while the lipophilic part is represented by alkyl chains, alkyl benzenes silicone derivatives, or polyoxypropylene chains [251]. The alkyl chain length significantly influences their lipidic membrane permeation ability, drug encapsulation efficiency, and release rate, with the longer chains enhancing permeation and entrapment while reducing release rates [253].

At the ocular level, these SAAs can be toxic, disrupting corneal and conjunctival epithelial lipid bilayers through lipophilic interactions, potentially releasing lysosomal enzymes, histamine, and inflammatory mediators [254]. Matsuda et al. [255], using rabbit corneal epithelial (RCE) models, reported a chain-length-dependent inhibitory effect on cell viability, with the polyoxyethylene lauryl ether showing the greatest impact, followed by the polyoexyethylene stearyl ether, the polyoxyethylene cetyl ether, and the polyoxyethylene behenyl ether. Despite this, numerous studies have reported their safe ocular application without at concentrations lower than 0.5% (*w*/*v*) [253,256,257,258,259,260,261], and, when adverse effects exist, they tend to be concentration-dependent [262].

Representative compounds used as permeation enhancers include polyoxyethylene 9-lauryl ether (BL-9), polyoxyethylene 23 lauryl ether (Brij 35), polyoxyethylene 20-stearyl ether (Brij 78), and polyoxyethylene 20 oleyl ether (Brij 99) [253,254,256,257,258,259,263,264].

The vast majority of current research is focused on the use of PADs for the systemic absorption of ocular topically applied compounds, mainly insulin [259,260,261,263,265,266], but also glucagon [267], oxytocin, vasopressin [264], β-endorphin [258], α-melanocyte stimulating hormone, somatostatin, vasoactive intestinal peptide, and adrenocorticotropic hormone [256,257]. Therefore, despite representing promising biocompatible corneal permeation enhancers, further studies are needed to evaluate PADs’ applicability in ocular tissues.

##### Polyoxyethylene Sorbitan Esters (Tween)

Polyoxyethylene sorbitan esters, widely known as Polysorbate or Tween, are classes of biocompatible non-ionic surfactants synthesized by the addition, via polymerization, of ethylene oxide to sorbitan fatty acid esters, which are formed by the esterification of sorbitol or sorbitan with a fatty acid [268,269].

Representative compounds of this class that are used as ocular permeation enhancers are Tween 20 (polyoxyethylene sorbitan monolaurate), Tween 40 (polyoxyethylene sorbitan monopalmitate), Tween 60 (polyoxyethylene 20 sorbitan monostearate), and Tween 80 (polyoxyethylene 20 sorbitan monooleate) [270].

Current studies tend to focus on the use of Tween 80, well known for its safe, non-toxic, non-irritating, and corneal-permeation-enhancing properties [271,272]. Recently, Barbalho et al. developed drug-loaded Tween 80 transferosomes (ultra-deformable elastic bilayer vesicles) that significantly enhanced ex vivo corneal and conjunctival curcumin permeation [273]. In addition, the latest research explores Tween 80 as a surfactant or edge activators in formulations, in combination with other permeation enhancers, surface-acting agents, or mucoadhesive compounds, such as spanlastic nanovesicular systems [274,275,276,277], nanostructured lipid carriers [278], hybridized vesicular systems with Labrasol [279], solid lipid nanoparticles [280,281], CH nanoparticles [219], sorbitan ester nanoparticles [282], nanoemulsified in situ ophthalmic gel [283,284], and microemulsion systems [285,286,287]. These formulations demonstrated increased corneal drug permeation and/or higher drug efficiency.

Studies using Tween 20 [288] and Tween 60 [289] in ocular drug delivery systems are less common, but results are similarly characterized by enhanced corneal drug permeation.

##### Sorbitan Fatty Acid Esters (Spans)

Sorbitan is a hexahydric alcohol derived from a dehydration reaction of sorbitol that is obtained by catalytic hydrogenation of glucose. The esterification of sorbitol with fatty acids leads to a variety of non-ionic surfactants commonly known as sorbitan esters, often referred as Span [290].

Span 20 (sorbitan monolaureate), Span 40 (sorbitan monopalmitate), Span 60 (sorbitan monostearate), and Span 80 (sorbitan monooleate) are examples of sorbitan fatty acid esters used as ocular permeation enhancers [270].

The majority of recent studies report results regarding Span 60 use in formulations such as surfactants and co-surfactants in combination with other permeation enhancers, surface-acting agents, or mucoadhesive compounds. Some examples of these formulations, showing increased corneal drug permeation and efficacy, are spanlastic nanovesicular systems [274,275,276,277,289,291], niosomal in situ gels [287,292,293,294], proniosomal gels [295], nanovesicular/self-nanoemulsifying systems [296], chrownsomes [178], and noisome-loaded in situ gelling ocular inserts [297]., The use of Span 60 in these formulations usually results in vesicles with larger particle size and increased encapsulation efficiency when compared to other compounds of the same class. Although less frequently, there are also studies that mention the use of Span 20, mainly for the development of microemulsion systems [285,286], Span 40 in proniosomal gel formulations [298], and Span 80 in self-nanoemulsifying drug delivery systems [90], with promising enhanced corneal permeation results.

##### d-α-Tocopheryl Poly(Ethylene Glycol) 1000 Succinate (VE-TPGS 1000)

VE-TPGS 1000, a stable derivative of natural vitamin E, is a non-ionic surfactant widely employed as a solubilizer, emulsifier, stabilizer, and vehicle in lipid-based drug delivery systems [299,300]. Formed by esterifying vitamin E with polyethylene glycol 1000, it comprises a hydrophilic PEG (polar head) and a lipophilic (vitamin E succinate) alkyl tail. Beyond its antioxidant and anticancer properties, VE-TPGS 1000 acts as a multi-drug resistance (MDR) protein inhibitor that modulates MDR-1 ATP-dependent drug efflux pumps present in the corneal epithelium, enhancing the ocular permeation of several drugs [301]. Studies have demonstrated its efficacy in increasing the corneal permeation of drugs like riboflavin [300], biotin-12-hydroxystearic acid-acyclovir [302], chlorhexidine [303], coenzyme Q10 [304,305], and brinzolamide [299] without causing ocular irritation or damage. The main studies on its corneal permeation enhancer properties can be observed in Table 5. This highlights its potential as a safe and effective permeability enhancer to be used in future ocular drug delivery systems.

##### Labrasol^®^

Labrasol^®^, a non-ionic surfactant, has recently shown significant potential as an ocular permeation enhancer due to its amphiphilic nature, which enables interactions with the lipidic components of the corneal epithelium. Guo et al. demonstrated that Labrasol enhances corneal permeability by down-regulating tight-junction-associated proteins such as F-actin, claudin-1, and β-catenin, thereby mainly increasing the paracellular transport route [306]. As summarized in Table 6, various studies have demonstrated the permeation-enhancing properties of Labrasol at different concentrations. Studies from Z. Liu et al. [307] and Huang et al. [308], identified 2.0% (*w*/*v*) Labrasol as the optimal concentration for maximizing its penetration-enhancing effects and reported significant synergistic benefits when combined with other permeation enhancers, like menthol or borneol. While its toxicity is concentration-dependent, Labrasol was considered safe at concentrations up to 2.0%, with no significant ocular irritation reported, and having a maximum safe concentration of 3.0%. Innovations like those of M. M. Ibrahim et al. [309], which incorporated Labrasol into a microemulsion formulation containing ribavirin, and significantly improved ribavirin’s corneal permeation, reasserting Labrasol’s value as an ocular drug permeation enhancer. Future research should focus on optimizing its concentration and exploring synergistic combinations with other enhancers to maximize its therapeutic efficacy while ensuring safety.

##### N-Methyl-2-Pyrrolidone (NMP)

NMP is a solvent with high power to solubilize chemicals and pharmaceutical agents, also functioning as a surfactant in the ocular medium, enhancing cellular penetration for both hydrophilic and lipophilic drugs [279]. Upon interaction with the lipid bilayer of the outer cell membrane, NMP is believed to induce polar defects that alter the membrane’s physicochemical properties, leading to its solubilization. Additionally, NMP can form micelles, which remove phospholipids from epithelial cell membranes, thereby increasing transcorneal drug permeability [280]. Table 7 reveals the existing ocular-level studies on NMP-permeation-enhancing properties. NMP proved to be an effective solvent and permeation enhancer in a concentration-dependent manner, particularly from 2.5% to 10%, where it significantly improves the apparent corneal permeability coefficients (Papp) for both lipophilic and, to a greater extent, hydrophilic drugs. At concentrations between 0.1% and 1%, NMP demonstrates limited corneal permeability enhancement [311]. NMP is considered to be non-irritating at concentrations up to 10%, slightly irritating at 15%, and moderately irritating at 20% [312]. These properties suggest that NMP could be a promising permeation enhancer in ocular drug delivery, but further research is required to optimize its concentration and comprehensively evaluate its safety profile.

##### Lecithin

Lecithin is a surface-active phospholipid-rich mixture, typically containing choline, choline esters, palmitic, stearic, and oleic acids, along with glycerol, glycolipids, triglycerides, and phosphoric acid. It is commercially derived from the degumming of crude vegetable oils (soybean, cottonseed, corn, sunflower, and rapeseed) but can also originate from animal sources such as egg yolk or milk fat [313]. Widely utilized in the food and pharmaceutical industries as a surfactant, wetting agent, release agent, and for viscosity reduction and crystallization control, lecithin owes its functional and therapeutic properties to its phospholipid content [314,315].

Phosphatidylcholine (PC), the predominant phospholipid in both plant and animal lecithins, plays a fundamental role in eukaryotic cell membranes, forming a dynamic bilayer that ensures membrane integrity and facilitates essential processes, such as vesicular trafficking, membrane fusion, protein mobility, and signal transduction [313,316,317,318].

Due to their strong affinity for biological membranes, phospholipid-based formulations enhance adhesion, permeation efficiency, and bioavailability of active molecules, making them promising ocular permeation enhancers [319].

Several studies have highlighted the potential of lecithin-based systems for ocular drug delivery. Among some of the notable advancements, Chetoni et al. developed phosphatidylcholine- and cholesterol-based liposomes encapsulating Distamycin A (DA) for the treatment of acyclovir-resistant Herpes simplex virus keratitis. The formulation achieved an encapsulation efficiency of 34.53% [320]. Comparative studies in rabbit corneal epithelial cells revealed that DA-loaded liposomes (DA-Lipo) exhibited lower cytotoxicity than the DA solution. Furthermore, DA-Lipo demonstrated reduced elimination of DA post-instillation, leading to a 1.73-fold increase in DA bioavailability in tear fluid and a 1.28-fold enhancement in DA uptake by the cornea.

Expanding on these findings, Tan et al. explored CH-coated liposomal formulations for the ocular delivery of timolol maleate utilizing soybean phosphatidylcholine and cholesterol as lipid components [321]. The uncoated TM liposomes exhibited a drug entrapment efficiency (EE%) of 70.19 ± 1.48%, with an in vitro transcorneal permeation study revealing a 1.88-fold increase in the apparent permeability coefficient (Papp) compared to TM solutions. Notably, CH-coated liposomes demonstrated superior performance, achieving an EE% of 75.83 ± 1.61% and a Papp enhancement of 3.18-fold relative to TM solutions and 1.69-fold compared to uncoated liposomes. The improved permeation was attributed to lipid vesicle biocompatibility and CH-induced tight junction disruption, facilitating drug transport.

In line with this progression, Londhe and Sharma, developed a novel ophthalmic formulation of methazolamide (MTA) by incorporating the drug into a phosphatidylcholine and cholesterol-based liposomal in situ gelling system, intended for the management of glaucoma [322]. The optimized formulation exhibited a high encapsulation efficiency of 74.12 ± 0.52%. Compared to the conventional MTA solution, both the MTA-loaded liposomes and the liposomal in situ gel significantly enhanced intraocular pressure (IOP) reduction. Notably, the MTA liposomal gel demonstrated a sustained pharmacological effect, maintaining IOP-lowering activity for over 8 h, whereas the effect of the MTA solution was limited to approximately 6–8 h. These findings underscore the superior retention and prolonged therapeutic efficacy of the MTA liposomal in situ gel over both the plain liposomal dispersion and the conventional drug solution.

Further advancements in lecithin-based ocular drug delivery were demonstrated by Peng et al., who engineered methoxy polyethylene glycol- and CH-modified egg yolk lecithin and cholesterol flexible liposomes embedded in thermosensitive sol–gel reversible hydrogels for the delivery of astragaloside IV (AS-IV) and tetramethylpyrazine (TMP) in the treatment of age-related macular degeneration [323]. The formulation achieved entrapment efficiencies of 85.32 ± 0.28% for AS-IV and 73.89 ± 0.15% for TMP. Compared to the control solution, flexible liposomes significantly enhanced the bioavailability of AS-IV by 1.68-fold and TMP by 2.33-fold, with further improvements observed upon modification with mPEG-CS-FL and mPEG-CS-FL-TSG. The study underscored the remarkable drug-loading capacity of flexible liposomes and their ability to navigate ocular barriers due to their exceptional deformability, reinforcing their potential for ocular therapeutics.

Lecithin has demonstrated immense potential as an ocular permeation enhancer, primarily due to its phospholipid composition, biocompatibility, and strong affinity for biological membranes. Its ability to improve drug permeability, precorneal residence time, and bioavailability makes it a highly attractive component in advanced drug delivery systems. However, certain limitations remain, including variability in composition depending on the source, potential stability concerns, and the need for optimized formulations to ensure consistent drug release. Future research should focus on overcoming these challenges through the development of standardized lecithin-based carriers, innovative surface modifications, and hybrid delivery formulations that maximize therapeutic efficacy while maintaining long-term stability. Lecithin-based nanocarriers hold great promise for revolutionizing ocular pharmacotherapy, paving the way for more effective and patient-friendly treatments.

#### 5.5.2. Cationic Surfactants

##### Benzalkonium Chloride (BAC)

BAC is a cationic surface-acting first-generation quaternary ammonium compound that is the most used preservative in ophthalmic preparations. Its primary role is to provide antimicrobial activity against Gram-positive and Gram-negative bacteria, viruses, and some fungi, preventing contamination of the vial and thus maintaining the sterility and extending the shelf life of eye drops [324,325,326,327]. BAC interacts with negatively charged molecules and phospholipid bilayer membranes through its positively charged ammonium group [328]. In this process, BAC’s hydrophobic long alkyl chain is integrated into the cell membrane, destabilizing cells’ lipid structure and leading to its dissolution, thereby causing intracellular content leakage and cell death, resulting in corneal epithelial cell loss [329]. Therefore, BAC acts as an ocular drug permeation enhancer mainly through disruption of the hydrophobic barrier of the corneal epithelium and disruption and expansion of the intercellular space [330,331,332]. Additionally, it can penetrate and modify the structure and features of the tear film lipidic layer [333]. Several in vitro studies have already demonstrated that BAC improves drug permeation through the cornea, which would, in theory, lead to increased bioavailability in the anterior chamber [164,334,335,336,337]. However, in vivo studies suggest that the difference in permeation is not statistically significant [338]. For instance, several studies report that preservative-free latanoprost formulations show equivalent efficacy compared to those with BAC to reduce intraocular pressure [339,340,341,342,343,344,345,346,347,348], while others contradictorily demonstrate an improvement in the corneal epithelial barrier function [344].

It has been known, for decades, that corneal exposure to BAC can disrupt its epithelial cells’ architecture and function, and the extent of these changes is influenced by both the concentration and duration of the exposure [349]. The precise mechanism through which BAC exerts cytotoxic effects on ocular tissues remains incompletely understood, yet it seems that its interaction with the mitochondria, the sole negatively charged intracellular compartment, plays a critical role [350,351,352]. Despite its widespread use at concentrations ranging from 0.004% to 0.025%, BAC has been shown to be cytotoxic even at concentrations as low as 0.005% [330]. BAC exposure has been associated with damage to the cornea, conjunctiva, trabecular meshwork, and ciliary epithelial cells [353,354,355,356,357,358,359,360,361]. It can increase levels of inflammatory markers in ocular tissues [358,359,360], interfere with cells’ gene expression [361], reduce conjunctival goblet cell density [362,363,364], and hinder corneal wound healing [365]. Clinically, BAC-induced toxicity usually manifests as pain, discomfort (e.g., stinging, itching, and ocular dryness) [366,367], increased tearing [367], increased fluorescein staining of the conjunctival and corneal epithelial surfaces [367,368,369,370], decreased tear break-up time [367,368,369,371,372,373,374,375], lower Schirmer scores [367,369], and a higher prevalence of punctate keratitis [371,372,376].

Future ophthalmic research on BAC must focus on reducing its cytotoxicity while maintaining its antimicrobial efficacy. Preservative-free alternatives and drug delivery systems that enhance bioavailability without disrupting the corneal epithelium should be prioritized for long-term therapeutic use.

##### Chlorobutanol (CB)

CB is an alcohol-based preservative with broad-spectrum antimicrobial activity. Its mechanism of action involves the disorganization of the lipid structure of the cellular membrane, thereby increasing its permeability [377]. Additionally, CB reduces oxygen utilization in the cornea, resulting in loosened epithelial cell adhesion, and induces the formation of vacuoles in epithelial cells, increasing drug corneal permeation [378].

An in vitro study by Camber et al. demonstrated that CB at 0.5% significantly improved corneal permeation of pilocarpine and dexamethasone. In vivo studies have revealed that, at a concentration of 2%, CB induces minimal toxicity. In comparison, benzalkonium chloride at the same concentration induces significant damage, including almost complete destruction of the corneal epithelium and endothelium [379]. This may reassure CB safety and potential suitability as a preservative in ocular formulations [380]. In contrast, Chandran et al. reported corneal hydration values suggestive of CB’s adverse effects on the corneal cell structure and integrity despite the markedly increase in flurbiprofen’s Papp, showing the maximum enhancement capacity between all the tested compounds [378].

CB is a promising substance to improve corneal drug permeation, but it may compromise corneal cell structure and integrity, justifying further research to optimize its safety and efficacy.

##### Cetylpyridinium Chloride (CPC)

CPC is a monocationic quaternary ammonium compound with surfactant properties and notable antibacterial and antiviral activity [381,382]. Its mechanism of action involves disrupting negatively charged bacterial membranes, thereby impairing osmoregulation and homeostasis, ultimately causing membrane disintegration and cytoplasmic leakage [381]. Regarding viruses, it interacts and disrupts the integrity of the viral lipid envelope, inhibiting viral fusion with target cells [382,383].

CPC has also been shown to improve corneal permeability affecting not only the cellular membrane but also increasing intercellular space width and thus the paracellular route [334,384,385]. Notably, Green et al. showed that CPC could enhance in vitro corneal permeability to sodium fluorescein, which was later corroborated by other studies [334]. Godbey et al. reported that 0.02% CPC demonstrated an efficacy comparable to a complete removal of the corneal epithelium in enhancing corneal absorption of penicillin G [384]. Additionally, Chetoni et al.considered CPC capable of promoting the corneal permeation of timolol maleate without significantly modifying the corneal resistance [165].

The most recent studies are focused on the use of CPC in the development of nanoemulsions and nanocrystals for ocular drug delivery, with no in vitro cytotoxicity and promising features for in vivo ophthalmic use, although corneal permeation studies are still lacking [386,387].

##### Chlorhexidine (CX)

CX, a cationic bisbiguanide surfactant, is a potent antiseptic widely used in medical and dental applications [388,389]. Its antimicrobial activity comes from its rapid binding to negatively charged bacterial cell walls and biofilms, leading to cell wall disruption and eventual cell death. At low concentrations, it induces a bacteriostatic effect by disrupting osmotic balance, while, at higher concentrations, it causes protein denaturation and cellular lysis. CX is particularly effective against Gram-positive bacteria, with moderate activity against Gram-negative bacteria, fungi, and lipophilic viruses [388,390].

Primarily utilized in dentistry, CX is found in oral rinses, gels, sprays, and dental varnishes, as well as in preoperative antiseptics and hand scrubs [390,391,392]. In ophthalmology, it is used as a treatment for *Acanthamoeba* keratitis and as a contact lens preservative at concentrations ranging from 0.003% to 0.05% [391]. Despite its utility, there are multiple studies demonstrating corneal toxicity when using CX-based solutions, frequently resulting in acute corneal changes, such as epithelial edema, bullous keratopathy, and loss of keratocytes and endothelial cells, resulting in various degrees of ulcerative keratitis with consequent delayed healing, pain, and vision loss [393,394]. Nevertheless, it seems to be generally well tolerated at concentrations of <0.02% (*w*/*w*), although mild irritation and corneal opacities have been reported even at 0.005% [303,395].

At the ocular level, CX electrostatically binds to the glycosaminoglycans in the corneal stroma, thus poorly penetrating this membrane [389]. However, there are studies showing that CX significantly enhanced corneal permeation of compounds like sodium fluorescein [336], pilocarpine, dexamethasone [379], sorbitol, and arnolol [389].

In conclusion, CX is a potent antiseptic with the ability to enhance corneal permeation for several drugs despite not having been explored extensively for this purpose. At low concentrations, CX is generally well tolerated, which highlights its potential as a corneal permeation enhancer, but further investigation should address its optimal effective concentration.

#### 5.5.3. Bile Acids and Salts

Bile salts (BSs), the primary components of bile, are synthesized in hepatocytes via enzymatic processes from cholesterol [396]. In humans, the liver produces primary bile acids—cholic acid (CA) and chenodeoxycholic acid (CDCA)—which are conjugated with taurine or glycine to form taurocholic and glycocholic acids, respectively [397,398]. These conjugated bile salts are secreted into bile, stored in the gallbladder, and later metabolized by intestinal bacteria into secondary bile acids such as deoxycholic acid (DCA) and lithocholic acid (LCA) [397,399]. CDCA can also be converted into ursodeoxycholic acid (UDCA) through bacterial and hepatic enzymatic activity [396].

In the duodenum, BSs play a critical role in lipid digestion by displacing surface-active proteins from fat droplets, facilitating the adsorption of pancreatic lipase and colipase [397]. They also solubilize lipolysis products into mixed micelles, enhancing their transport through the intestinal mucus and absorption at the epithelium [400]. Structurally, BSs are amphiphilic molecules with a steroid nucleus consisting of three six-membered rings and one five-membered ring with a curved or flat structure [401,402,403]. Hydroxyl groups on the concave side confer hydrophilicity, while methyl groups on the convex side impart hydrophobicity to these molecules [404]. This duality allows BSs to self-assemble into micelles at critical micellar concentrations (CMCs), forming either primary micelles at lower concentrations or secondary micelles at higher concentrations through intermolecular hydrogen bonding [400,405,406].

The self-assembly behavior of bile salts and their toxicity are mainly determined by their hydrophobicity, increasing in the following order: UDCA < CA < CDCA < DCA < LCA, UDCA being the most hydrophilic and LCA the most hydrophobic natural bile acid [407]. The CMCs correlate inversely with the hydrophobicity of bile acids. Simple bile acid micelles act as powerful detergents, converting lipid bilayers into mixed micelles, causing a membranolytic effect [408]. Mixed micelles are formed when BSs are combined with polar lipids, conventional surfactants, or amphiphilic drugs, enhancing solubilization of encapsulated molecules and their efficiency as permeation enhancers while reducing the risk of toxic membranolytic effects [405,409].

BSs can encapsulate hydrophilic molecules in secondary micelles and lipophilic molecules in the hydrophobic cavities of primary micelles, making them versatile and suitable compounds for the design of advanced drug delivery systems [405].

The BSs most frequently used in drug delivery systems include primarily sodium glycocholate (GCA), DCA, and sodium taurocholate (TCA), mainly because they have less toxic potential and have reported anti-inflammatory, anti-apoptotic, and antioxidant effects [410]. As summarized in Table 8, they enhance the ocular permeability of peptides and drugs, favoring the transcellular pathway, with glycocholate showing activity at both paracellular and transcellular routes. GCA and DCA seem to have dual action on the membrane and tight junctions; however, GCA seems to be more active at the paracellular than at the transcellular pathway. TCA seems to have a primary effect on the membrane and negligible at the paracellular pathway [411]. However, DCA, the strongest irritant among BSs to the cornea and conjunctival cells, induces dose-dependent membrane damage, indicating that this BS might not be a suitable enhancer for ocular drug delivery [165,337,410,412]. Irritant activity increases in the order tauroursodeoxycholate (TUDC) < UDCA < taurodeoxycholate (TDC) < DCA [337].

BSs exhibit significant potential as drug delivery enhancers. Among them, GCA and TCA have shown promise in ocular drug delivery. Future research should focus on optimizing BS formulations to balance permeability enhancement with safety, particularly by exploring conjugated and modified bile salts with reduced irritant effects. Investigating synergistic combinations with other permeation enhancers and designing BS-based nanocarriers could further improve bioavailability while minimizing adverse reactions. Additionally, mechanistic studies on their interactions with biological membranes and tight junctions will be crucial to refining their role in advanced drug delivery systems.

#### 5.5.4. Glycosides

Glycosides are a diverse group of plant secondary metabolites characterized by the presence of one or more sugar molecules (glycone) combined with a bioactive non-sugar moiety (aglycone) via a glycosidic bond. In plants, glycosides primarily serve as energy storage molecules in the form of inactive sugars, which can be activated by hydrolyzation, releasing the bioactive aglycone unit when needed. This versatile class of compounds includes hormones, sweeteners, alkaloids, flavonoids, antibiotics, and other biologically significant molecules [414].

##### Saponins (SPs)

SPs are a diverse class of naturally occurring high-molecular-weight amphiphilic compounds with a broad range of physical, chemical, and biological properties, often serving as defense mechanisms in plants [415,416]. Structurally, they consist of a lipophilic sapogenin core (either a steroid or triterpene, classifying them as steroidal or triterpenoid SPs) and a hydrophilic glycone component comprising one or more carbohydrate chains. These two regions are linked by a glycosidic bond, and SPs are further categorized as mono-, di-, or tridesmosidic based on the number of carbohydrate chains [415,417]. A defining feature of SPs is their surface-active properties, derived from their ability to reduce surface tension in aqueous solutions and form micelles at concentrations above their CMC [415,418]. This feature makes them effective solubilizing agents. Their lipophilic aglycone regions can interact with cell membranes, disrupting their integrity and potentially causing the extrusion of cytoplasmic components [419]. Consequently, SPs have been investigated as ocular permeation enhancers, particularly to facilitate topically applied ocular drugs’ systemic absorption [261,263,266,267,420,421,422]. When applied topically, and after trespassing the tear film, SPs interact first with the corneal epithelium, a highly lipophilic barrier that limits the absorption of hydrophilic drugs. By destabilizing this structure, SPs increase the permeability of hydrophilic drugs while causing mild effects on lipophilic compounds [337,423]. However, their interaction with cell membranes is also responsible for their well-documented hemolytic and irritative properties, particularly at ocular levels [261,262,266,267,337,423]. Table 9 shows the main studies on corneal-permeation-enhancing effects of SPs. Recently, SPs have shown promising results when included in ocular delivery systems as in situ pH-triggered gelling systems, significantly improving the in vitro corneal permeation without noticeable corneal, conjunctival, or iridal damage [424].

In general, to mitigate adverse effects, SPs must be used at the lowest effective concentration to ensure sufficient drug permeation enhancement across the cornea while avoiding excessive cell lysis and associated side effects. Future research on SPs such as ocular permeation enhancers should focus on optimizing their formulation to balance efficacy and safety. Exploring structural modifications or conjugation with biocompatible carriers could help to mitigate their hemolytic and irritative properties while preserving their permeability-enhancing effects. Additionally, investigating SPs in combination with other permeation enhancers or mucoadhesive systems may provide synergistic benefits, improving drug retention and controlled release. Advanced nanotechnologies, such as SP-based nanoparticles or hybrid vesicular systems, should also be explored to enhance drug targeting while minimizing toxicity.

##### Digitonin (DG)

DG, an amphiphilic steroidal saponin derived from the foxglove plant (*Digitalis purpurea*), is known for its powerful lytic activity on biological membranes [428], and it is widely used as a non-ionic surfactant with detergent properties [429]. DG solubilizes membrane proteins and lipids, which permeabilizes cholesterol-containing membranes and allows high-molecular-weight cytosolic compounds to be released [429]. For decades, it has been used to desquamate corneal epithelial cell layers, to selectively disrupt plasma membranes, and to study membrane permeability to ions, metabolites, and enzymes [430,431,432,433,434]. DG specifically binds to cell membrane cholesterol, forming rigid cholesterol–digitonin complexes that disrupt lipidic organization and increase membrane permeability without causing significant membrane destruction [434,435].

In ocular drug delivery, Liaw & Robinsonfirst reported that DG was able to enhance the corneal permeation of compounds, such as polyethylene glycols of varying molecular weights [436]. However, this effect came with severe alterations in the corneal epithelium, which raised toxicity concerns.

Subsequent studies, like those of Saettone et al. [337], demonstrated that even low concentrations of DG (0.0025%) slightly enhanced the corneal permeability of drugs like timolol maleate and betaxolol hydrochloride, but higher concentrations caused significant corneal swelling and opacification, as highlighted in Table 10. Other permeation enhancers demonstrated superior efficacy with fewer adverse effects.

Given these findings, DG use must be carefully considered because, despite its properties as an ocular permeation enhancer, its potential for corneal toxicity ends up limiting its practical application. Safer and more effective alternatives should be prioritized for ocular drug delivery systems.

##### Escin

Escin, a natural mixture of triterpene saponins derived from the seeds and seed shells of *Aesculus hippocastanum* (horse chestnut), exhibits superior anti-inflammatory and anti-edematous properties through glucocorticoid-like activity without inducing the typical glucocorticoid-associated adverse effects [438,439,440]. Furthermore, it also enhances venous tone, improves venous hemodynamics, and has endothelial-protective and potential anti-oxidative properties [441,442]. At the ocular level, when combined with glucocorticoids, it demonstrated synergistic protective effects on blood retinal barrier breakdown [443,444]. Table 11 outlines the few studies on the application of escin at the ocular level as a permeation enhancer, suggesting that it can act as a corneal permeation enhancer, significantly increasing the corneal permeability of timolol maleate at concentrations as small as 0.015% without causing substantial corneal hydration changes. However, higher concentrations may induce similar adverse effects to digitonin [337,445].

Overall, while escin appears to be a promising ocular permeation enhancer, further research is required to fully understand and establish its efficacy and safety across a broader range of drugs and therapeutic ocular applications.

### 5.6. Azone (1-Dodecylazacycloheptan-2-One)

Azone (1-dodecylazacycloheptan-2-one or laurocapram) is a hybrid cyclic amide derivative with a polar headgroup within a seven-membered ring attached to a C12 [446,447]. Originally developed as a skin penetration enhancer, this lipophilic compound has low toxicity and irritating potential, minimal pharmacological activity, and high compatibility with various solvents [447].

In dermatological applications, azone interacts with the lipid domains of the stratum corneum, particularly those present in the intercellular channels, increasing fluidity and reducing diffusional resistance in this region, mainly supporting the intercellular pathway for drug delivery [447,448,449]. It effectively enhances the permeation of both hydrophilic and lipophilic compounds at concentrations between 0.1% and 5%, being often used between 1% and 3% [447,450]. Table 12 presents key studies that demonstrate that, at the ocular level, azone’s mechanism of action is less understood but appears to involve modifications in the epithelial junctions and increased hydration, thus enhancing the permeability and, potentially, the transcorneal permeation of hydrophilic drugs, yet reducing lipophilic drug penetration by creating a more hydrated barrier [451,452].

Early studies demonstrated its efficacy as an ocular permeation enhancer, with Newton et al. showing improved corneal drug levels and faster steady-state achievement when cyclosporine was formulated with azone [453]. Subsequent research by Ismail et al. [454] and Tang-Liu et al. [452] confirmed significant enhancements in corneal absorption, particularly for hydrophilic drugs that are reliant on paracellular pathways, with permeability coefficients increasing up to 185-fold for drugs like guanethidine. There were marginal or negative enhancement effects on mild lipophilic compounds. More recent authors, like Mao et al. [455], reported improved corneal permeability of gadolinium-diethylene triamine penta-acetic acid (Gd-DTPA), while Abrego et al. [451] demonstrated enhanced anti-inflammatory efficacy of pranoprofen-loaded nanoparticulate formulations containing azone in the cornea, significantly reducing ocular edema without causing irritation (Table 12).

Overall, research continues to highlight azone’s potential as a safe and effective ocular permeation enhancer, particularly for hydrophilic drugs. Nevertheless, additional investigation is needed to refine its applications and fully understand its mechanisms of action.

### 5.7. Cell-Penetrating Peptides

Cell-penetrating peptides (CPPs) or Protein Transduction Domains (PTDs) are a class of short peptides, generally consisting of 5–30 amino acids, that, unlike most of them, can translocate through cell membranes without compromising their integrity [459]. Therefore, they can promote the movement of a cargo, such as nanoparticles, liposomes, small interfering RNA, double-stranded DNA, and several drugs, across the cell membrane into the cytoplasm and facilitate interactions with the target [460,461]. This concept was introduced over twenty years ago, when it was observed that some proteins, mainly transcription factors, could permeate into and between cells [462].

#### 5.7.1. Penetratin (PNT)

PNT (RQIKIWFQNRRMKWKK-NH2) is a 16-amino-acid cationic CPP derived from the third helix of the *Drosophila antennapedia* homeodomain that has emerged as one of the safest and most potent enhancers for corneal drug delivery. It is commonly used as cellular permeation enhancer of nanoparticles, often being covalently linked to their surface [463].

PNT has demonstrated distinctive corneal-permeation-enhancing ability, higher than that observed for TAT and other CPPs like pVec, L17E, protamine, and poly(arginine)_8_ [464,465]. PNT corneal-permeation-enhancing capacity is concentration-dependent, varying also with its hydrophobicity and with the cellular active transport pathways (endocytosis). Furthermore, when PNT is combined with nanoparticles, the drug permeation tends to decrease with the increase in the particle size [466].

Jiang et al. designed a series of PNT derivatives by replacing its main hydrophilic amino acids with the hydrophobic amino acid tryptophan and reported that these hydrophobic derivatives (28-W, 29-W, 89-W, and 289-W) showed improved corneal and scleral permeability and fast drug distribution into the retina, with higher intraocular bioavailability when compared to PNT [461]. Nevertheless, more recently, Morofuji et al. used a reconstructed human corneal epithelial tissue model, LabCyte CORNEA-MODEL2, and reported that PNT-289W cellular uptake was significantly lower than that of PNT [465]. This study suggested that appropriate hydrophilicity is required for corneal uptake and that further research should be carried out to evaluate the ability of these derivatives as corneal permeation enhancers.

PNT has been successfully used as a corneal permeation enhancer for noninvasive topically applied gene delivery systems targeting the posterior segment of the eye [467], for topically applied co-modified PEGylation polyamidoamine (PAMAM) nanocarriers targeting the posterior segment of the eye for the treatment of choroidal neovascularization in age-related macular degeneration (AMD) [468], to enhance corneal permeation of a topically applied co-delivery system capable of transporting fluorouracil and anti-TGF-b2 oligonucleotides to synergistically inhibit fibroblast proliferation and consequently post-trabeculectomy fibrosis [469], to aid retinal delivery of lutein in a nanoemulsion in situ gel for the treatment of AMD to deliver lutein to the retina [470], for the delivery of small interfering RNA (siRNA) by a penetrating derivative (89WP)-modified polyamidoamine polyplex via eye drops to achieve gene silencing in orthotopic retinoblastoma [471], to enhance the corneal permeation and posterior ocular segment bioavailability of dexamethasone [472], for dual-modified ophthalmic liposome eye drops to deliver anti-vascular endothelial growth factor as therapy for neovascular age-related macular degeneration to the posterior segment of the eye [473], and for the co-delivered of dexamethasone sodium phosphate loaded into contact lenses [474]. Overall, PNT’s versatility, safety, and effectiveness as a corneal permeation enhancer make it a valuable tool for advancing ocular drug delivery systems. However, further research is necessary to refine its derivatives and fully explore its potential in broader applications.

#### 5.7.2. Trans-Activator of Transcription (TAT) Protein Transduction Domain

The Trans-Activator of Transcription (TAT) protein from Human Immunodeficiency Virus Type 1 (HIV-1) has emerged as a key molecular tool in biomedical research due to its unparalleled ability to mediate intracellular delivery of diverse therapeutic agents. First identified in 1988 for its capacity to translocate cellular membranes and localize within the nucleus, this property is attributed to its highly cationic, arginine-rich protein transduction domain (PTD) [475,476]. The TAT PTD interacts electrostatically with anionic phospholipids on the cellular membrane surface, enabling its internalization through multiple endocytic pathways, including macropinocytosis and clathrin- and caveolae-mediated endocytosis [477,478,479,480,481]. Importantly, its membrane translocation efficiency is influenced by factors such as peptide length, charge density, and secondary structure, which have been explored to optimize its functional application.

In ophthalmic drug delivery, TAT PTD has demonstrated significant potential as a permeation enhancer by overcoming the physiological barriers of the eye (Table 13). Wang et al.reported that TAT PTD markedly enhanced the corneal penetration of topically applied human acidic fibroblast growth factor (aFGF19-154), effectively facilitating drug delivery across ocular barriers and conferring neuroprotective effects against retinal ischemia–reperfusion injury [482]. Similarly, Zhang et al. utilized the TAT PTD to improve the ocular permeation of topical endostatin, a specific inhibitor of endothelial cell proliferation and angiogenesis [477]. Their findings demonstrated successful distribution of endostatin to the retina and choroid, where it exhibited enhanced inhibition of pathological neovascularization, an effect further potentiated by the fusion with an arginine–glycine–aspartic acid (RGD) tripeptide [483]. Expanding the scope of TAT PTD applications, Zhu et al.developed a modified TAT peptide incorporating the N-terminal 24 amino acids of the p55 regulatory subunit of phosphatidylinositol-3-kinase (p55PIK) [484]. This construct had previously exhibited significant in vitro anti-inflammatory and anticancer properties, and, when topically administered, it effectively suppressed intraocular inflammation in both endotoxin-induced uveitis and experimental autoimmune uveitis models, being considered a potential candidate for the treatment of ocular inflammatory diseases and further studied to alleviate suture-induced corneal neovascularization and inflammation [485]. Further supporting its role as an ocular permeation enhancer, in 2021, Rohira et at. demonstrated that a TAT-natamycin conjugate significantly improved the corneal penetration and antifungal efficacy of natamycin, used as a standard treatment for fungal keratitis [486]. More recently, Thareja et al. reported that conjugation of dexamethasone sodium phosphate with TAT significantly increased its transcorneal apparent permeability coefficient, reinforcing its potential as an ocular permeation enhancer [472].

Beyond direct drug conjugation, TAT PTD has been extensively employed in the functionalization of advanced ocular drug delivery formulations, including nanoparticles [487,488,489,490], human-mesenchymal-stem-cell-derived exosomes [491], liposomes [492,493], and graphene nanocarriers [494]. These systems have yielded promising results, demonstrating improved drug stability, bioavailability, and targeted delivery within ocular tissues.

Collectively, these findings underscore the transformative potential of TAT PTD in ophthalmic therapeutics. By facilitating non-invasive intracellular delivery and enhancing corneal penetration, TAT PTD-mediated strategies hold significant promise for improving the efficacy of ocular treatments. Future research should focus on optimizing peptide modifications to enhance stability, minimize cytotoxicity, and achieve precise targeting, ultimately paving the way for its clinical translation in next-generation ophthalmic therapies.

**Table 13:** Main studies on corneal permeation enhancer properties of TAT.

Cargo and Molecular Weight	Permeation Outcomes	Adverse Reactions	References
Human Acidic Fibroblast Growth Factor (aFGF19-154 or FGF-1) (16.0 kDa)	TAT aFGF-His showed a rapid ocular penetration, detected in retina within 30 min, mediating strong protection against retinal IR injury.	Not mentioned.	[263]
Endostatin (20 kDa)	Significantly enhanced penetration to retina and choroid. Micropinocytosis was the dominant uptake mechanism for TAT PTD.	Not mentioned.	[420]
Endostatin arginine–glycine–aspartic (20.3 kDa)	Improved permeability and higher inhibition of neovascularization.	Not mentioned.	[483]
Flurbiprofen (0.24 kDa)	TAT-functionalized, flurbiprofen-loaded liposomes reduced the drug loss rate from the eye surface and enhanced the intraocular delivery of flurbiprofen.	No toxicity observed.	[495]

#### 5.7.3. Polyarginines (PLAs)

Polyarginines are short peptides consisting of repeating arginine residues, typically ranging from 5 to 15 in length. Their cationic nature facilitates their interactions with negatively charged components of cell membranes, but the high density of guanidinium groups on arginine residues plays a major role regarding efficient electrostatic interaction with negatively charged cell membranes and cellular uptake [496,497,498]. Furthermore, PLAs are reported to change the structure and induce the dissociation of tight junctions, increasing the paracellular permeation of hydrophilic molecules [499,500]. At the ocular level, PLAs have been associated with an increase in paracellular permeation in the conjunctival and corneal epithelium [501,502].

Nemoto et al. designed the first study dedicated to understanding the enhancing effect of PLAs on the ex vivo permeability of hydrophilic compounds through the ocular epithelia [501]. These authors demonstrated that PLAs caused a reversible reduction in the transepithelial electric resistance (TEER) of the conjunctiva and significantly enhanced the permeation of FITC-labeled dextran and pyridoxamine through the cornea, conjunctiva, and conjunctiva/sclera composite. Their in vivo study reported that PLAs significantly enhanced the concentration of pyridoxamine and FITC-labeled dextran in the aqueous humor and the vitreous body without inducing any corneal alteration [503]. More recently, Liu et al. designed a novel octa-arginine-modified lipid emulsion system for the ocular delivery of disulfiram that was concluded by in vitro and ex vivo studies to exhibit the highest permeability and the largest amount of drug homogeneously distributed in ocular tissues compared with other preparations, further demonstrating a marked anti-cataract effect [502].

Polyarginines offer a promising approach for enhancing ocular drug delivery by overcoming physiological barriers and improving drug bioavailability. Their ability to facilitate cellular uptake and modulate tight junctions makes them valuable tools in ophthalmic formulations. However, further research is needed to optimize their safety profile and efficacy for clinical applications.

#### 5.7.4. Pep-1

PEP-1 (Ac-KETWWETWWTEWSQPKKKRKV-cysteamine) is a synthetic CPP designed to efficiently deliver macromolecules, such as proteins, peptides, and nucleic acids, into cells in a fully biologically active form [504]. Its amphipathic structure consists of a hydrophobic domain (KETWWETWWTEW) that interacts with negatively charged proteins or the phospholipid bilayer of cell membranes, a hydrophilic Lys-rich domain (KKKRKV) derived from the nuclear localization signal of SV-40 that enhances solubility and intracellular distribution, and a flexible spacer (SQP) maintaining structural integrity of the other two domains [505,506,507]. The dehydration induced by the hydrophilic domain at membrane surface and the insertion of hydrophobic domain promote membrane destabilization, facilitating the translocation of PEP-1 and therapeutic molecules into cells. There is no involvement of the endocytic pathway or evidence of pore formation or leakage of cytoplasmic components [506,507,508,509]. The main driving force for peptide translocation is a charge gradient across membrane (negative inside) [507]. Alterations in membrane permeability only occurred for high peptide/lipid ratios, which induced complete membrane disintegration [506]. PEP-1 establishes a variety of electrostatic and/or hydrophobic and/or hydrophilic interactions with the cargo [508].

A study by Pescina et al.investigated the permeability of various CPPs, including PEP-1, across the corneal epithelium [505]. They demonstrated that PEP-1 and its derivative pep-7 preferentially locate in the intercellular spaces and in the plasma membrane, suggesting that they diffuse across the corneal epithelium mainly following the paracellular route, which implies facilitated drug delivery by modulation of tight junctions and increasing intercellular space. Both peptides proved to be safe when investigated for their in vitro cytotoxicity in conjunctival cell lines at the concentrations used.

Kim et al. demonstrated that PEP-1 can be efficiently transduced into human corneal epithelial cells (HCE-2) as well as mouse corneal and conjunctival tissue in a dose- and time-dependent manner [73]. Furthermore, topical application of PEP-1-FK506BP (has anti-inflammatory effects in macrophage cells and animal inflammation models) to botulinum-toxin-A-induced mouse dry eye model proved to effectively exert its anti-inflammatory effects, significantly decreasing the amount of corneal fluorescein staining and markedly inhibiting the expression levels of pro-inflammatory cytokines and macrophage inhibitory factor in corneal and conjunctival epithelium. The results were corroborated by more recent studies where the topical application of PEP-1-FK506BP markedly increased the tear volume and significantly prevented corneal and conjunctiva damage in a low-humidity-air-flow-induced dry eye rat model [510] or significantly decreased the number of cells expressing pro-inflammation, apoptotic, and angiogenic factors, consequently diminishing both corneal opacity and corneal neovascularization and accelerating corneal wound healing in an in vivo rat model of an alkali-burn-induced corneal inflammation [511].

Moreover, PEP-1’s versatility extends to its ability to deliver hydrophilic and hydrophobic drugs, as well as large biomolecules that typically face challenges in crossing the hydrophobic corneal barrier. By facilitating effective drug penetration, PEP-1 could potentially reduce dosing frequency, minimize systemic exposure, and improve patient compliance, which are critical goals in the management of chronic ocular diseases. While direct comparisons between PEP-1 and traditional preservatives like benzalkonium chloride (BAC) are limited, the biocompatibility of PEP-1 suggests it may be a safer alternative to agents known to induce oxidative stress and inflammation. Further research is necessary to comprehensively evaluate PEP-1’s efficacy and safety in ocular drug delivery applications. While the potential of PEP-1 is promising, further research is required to optimize its formulation, evaluate its long-term safety, and confirm its efficacy in clinical settings. Challenges such as potential immune responses, production costs, and large-scale manufacturing also need to be addressed. Nevertheless, PEP-1 represents a significant advancement in the field of ocular drug delivery, offering a novel approach to overcoming the barriers of the eye and enhancing therapeutic outcomes.

### 5.8. Cytochalasins

Cytochalasins are a group of small heterocyclic compounds discovered in 1964 as fungal metabolites by Carter, who suggested the name “cytochalasin” based on the Greek words cytos, cell, and chalasis, relaxation, for this new class of compounds based on the findings that they prevented cytoplasmic cleavage, inhibited cell motility, and turned cells flatter [512].

Soon after their discovery, they were already observed to have an impact on inter-cellular membrane attachment [513]. Cytochalasins bind specifically to actin microfilaments, the major component of the cell cytoskeleton, inhibiting the association and dissociation of actin monomers at their barbed ends, which alters their polymerization and network formation [514,515,516,517]. This leads to a marked disorganization of the actin microfibrillar network of the cytoskeleton that extends to the microfilament ring present immediately adjacent to the TJ and at the fine microfilament web spreading throughout the cytoplasm of confluent cells, which results in a widening of intercellular junctions (opening of the paracellular permeation route) and consequently in a decrease in the epithelial electrical resistance [518,519]. In addition to its normal role in regulating cell contractibility, mobility, shape, proliferation, intracellular transport, and cell-surface receptors, there is evidence showing that the cytoskeleton participates in the regulation of epithelial tight-junction integrity, which in addition to being essential for cell to-cell attachment, functions as a structural barrier that restricts the paracellular passage of hydrophilic molecules, cells, and water [520,521,522,523].

As the studies presented in Table 14 highlight, the effect of cytochalasin B appeared predominantly on the junctional portion of the epithelium, influencing the degree of opening of occluding junctions.

Future research should focus on optimizing cytochalasin-based formulations to enhance drug bioavailability into the posterior segment of the eye while ensuring safety and reversibility of action. Advanced nanoformulations, such as cytochalasin-loaded nanoparticles or hybrid biomaterials, may provide targeted and sustained drug release while minimizing cytotoxic effects. Moreover, mechanistic studies on the modulation of corneal and conjunctival epithelial integrity by cytochalasins could pave the way for innovative permeability enhancers in ophthalmic drug delivery. By refining their application, cytochalasins hold significant potential to revolutionize non-invasive therapeutic approaches for ocular diseases, offering safer and more effective treatment modalities, especially for molecules that permeate tissues paracellularly.

### 5.9. Terpenes

#### 5.9.1. Borneol

In traditional Chinese medicine and other Asian countries, this naturally occurring simple bicyclic monoterpene (C_10_H_18_O) has been widely used in the clinic for the treatment and prevention of eye diseases for almost a thousand years due to its analgesic, anti-inflammatory, antibacterial, freshening, and pain-relieving effects [526,527]. As a low-molecular-weight lipophilic compound, it can rapidly and reversibly rearrange the sequence of the phospholipids from the lipid bilayer of the corneal epithelium and loosen its tight junctions, leading to an increase in corneal permeation through both the transcellular and paracellular routes for lipophilic and, in a more significant way, hydrophilic drugs [89,526,527,528,529].

The first study on the impact of borneol on corneal permeation dates to 2005, when Chun-Jie et al. studied the impact of this compound on the penetration of both puerarin and timolol maleate eye drops through the cornea ex vivo [526]. All the concentrations used (0.025%, 0.05%, and 0.1% borneol) led to an increase in the apparent permeability coefficient (Papp) of puerarin eye drops, but only the highest concentration led to a slight increase in the timolol maleate eye drop group as compared to the control group, showing that borneol seemed to tend to favor lipophilic corneal permeation when compared to hydrophilic drugs. Following this investigation, Yang et al. developed a study with the aim of investigating the effect of synthetic and natural 0.1% borneol on corneal permeability of two lipophilic (indomethacin and dexamethasone) and three hydrophilic drugs (ofloxacin, ribavirin, and tobramycin) in vitro [529]. In contrast to the previously cited study [526], it was concluded that the permeation-enhancing effects of borneol on hydrophilic drugs were greater than on lipophilic drugs. For lipophilic drugs, the permeation-enhancing effects of borneol were greater on dexamethasone, and, for the hydrophilic drugs, whose hydrophilicity was in the following order: ofloxacin < ribavirin < tobramycin, the higher the hydrophilicity, the greater the permeation-enhancing effects of borneol. Furthermore, they did not find any statistically significant difference between the permeation-enhancing effects of synthetic and natural borneol. In 2013, Song et al. conducted in vitro transcorneal geniposide (Ge) permeation studies further demonstrating the effectiveness of borneol as a permeation enhancer even at concentrations as low as 0.01% [508]. They also described this enhancing effect as being temporary and closely related to the dose of borneol. Additionally, when compared with 0.5% EDTA, borneol at 0.02% and 0.04% was significantly more efficient in promoting Ge permeation across the cornea. In the same year, Qi et al. demonstrated that borneol could effectively enhance the Papp of hydrophilic compounds with molecular weight 4 kDa or less (sodium fluorescein and FITC-dextran 4 kDa) in a molecular-weight-dependent manner and the Papp of lipophilic rhodamine B [527]. The last published study on the effectiveness of borneol alone as a permeation enhancer dates from 2017. Mao et al., while studying the aqueous humor pharmacokinetics of seven alkaloids from Rhizoma Corydalis Decumbentis, concluded that, after ocular instillation of 3 mg/kg of Rhizoma Corydalis Decumbentis extracts, borneol 0.04% led to a significant increase in the aqueous humor’s concentrations of these alkaloids when compared to the administration of the extracts without borneol [530].

Regarding studies with the combined use of borneol and other permeation enhancers, two were found. Liu et al. carried out the first ex vivo study on the ocular combined application of borneol and menthol as enhancers of permeation for fluconazole through the cornea, finding that their mixed application was significantly more effective than when they were individually applied, the 0.2% mixture of borneol and menthol (weight ratio 1:3) having the best effect [89]. Huang et al. using the water- and fat-insoluble baicalin as model drug, revealed that the Papp values for borneol were significantly increased when compared with the control group in vitro but more importantly that, in the groups where penetration enhancers were combined, the Papp values were statistically increased compared to the same concentrations of individually applied enhancers in vitro [308]. Papp values of 0.1% borneol were increased to almost double when combined with 1% Labrasol and triple when combined with 2% Labrasol, the best result of the entire study.

According to all these authors, the maximum safe concentration of borneol was 0.2%, and, at the concentrations tested, borneol has not shown to exert damage to the corneal epithelium, nor have toxic or inflammatory reactions at the ocular level, proving its long-term clinical safety (Table 15).

#### 5.9.2. Menthol

Menthol, a naturally occurring cyclic terpene alcohol and the primary component of mint and peppermint from labiatae plants, has been widely utilized in pharmaceutical formulations, particularly in topical analgesics, antipruritics, anti-inflammatory drugs, ophthalmic ointments, and ocular drops and other herbal drugs for ocular application in traditional Chinese medicine [89,531]. Menthol acts as an agonist of the transient receptor potential melastatin-8 (TRPM8) channel, activating cold receptors on the ocular surface. This effect is associated with increased tear secretion, alleviating visual fatigue and ocular discomfort, making menthol a potential candidate for ocular therapeutics beyond its permeation-enhancing properties [532].

In ocular drug delivery, menthol exhibits a unique ability to enhance transcorneal drug penetration by preferentially distributing into the intercellular spaces of corneal epithelial cells and reversibly disrupting and increasing the fluidity of the epithelial lipid bilayer [89,531]. This modulation facilitates the transcellular transport of lipophilic molecules, while, at higher concentrations, menthol may also disrupt epithelial tight junctions, permitting paracellular transport of hydrophilic drugs [531]. These properties have led to its investigation as an ocular permeation enhancer for various pharmacological agents.

The first reported study on menthol’s role as an ocular permeation enhancer dates back to 2011, when Xu et al. demonstrated that menthol 0.05% and 0.1% (*v*/*v*) significantly increased the corneal permeability of dexamethasone disodium phosphate in vitro and 0.1% (*v*/*v*) enhanced its ocular bioavailability following topical administration in vivo. However, the enhancement was cornea-specific as menthol did not influence drug penetration into the sclera, vitreous, or retina-choroid regions [531].

Subsequent studies have corroborated these findings. J. Liu et al., in 2012 [89], reported that menthol (0.05% and 0.1%) significantly promoted the ex vivo corneal permeation of fluconazole [89]. Interestingly, the combined application of menthol and borneol (at a 1:3 weight ratio) resulted in a significantly greater permeation enhancement than either compound alone, suggesting a synergistic effect between these terpenoids. Similarly, Huang et al., using baicalin as a model drug, confirmed that menthol (0.1% and 0.2%) substantially increased the apparent permeability coefficient (Papp) in corneal transport assays [308]. Notably, their in vitro study was the first to demonstrate that the co-administration of menthol with another non-terpenoid permeation enhancer, Labrasol, led to a statistically significant increase in permeability compared to the individual enhancers. The most pronounced effect was observed with a formulation containing 2% Labrasol and 0.2% menthol, highlighting the potential for combinatory approaches to optimize ocular drug delivery.

More recently, Bai et al. investigated menthol’s ability to facilitate the corneal penetration of natamycin, a poorly water-soluble macrolide antifungal agent [533]. Their findings indicated that, while menthol alone did not significantly influence corneal drug permeation in vivo, the combination of menthol and iontophoresis significantly improved the therapeutic efficacy of natamycin in treating fungal keratitis, demonstrating a novel strategy for overcoming the drug’s poor ocular bioavailability. Importantly, the optimal concentration for permeation enhancement was determined to be 0.2% as higher concentrations did not yield additional benefits.

The safety profile of menthol as an ocular permeation enhancer has also been evaluated. The maximum safe concentration was established at 0.4%, beyond which potential toxicity concerns may arise [308,533]. At the tested concentrations, menthol has not demonstrated cytotoxicity, inflammatory responses, or long-term damage to the corneal epithelium, underscoring its potential for clinical translation (Table 16).

Taken together, these studies establish menthol as a promising, safe, and effective ocular permeation enhancer with diverse applications in drug delivery. Its ability to modulate corneal permeability, particularly in combination with other penetration enhancers or physical methods like iontophoresis, opens new avenues for improving the bioavailability of ophthalmic therapeutics. Future research should further elucidate its mechanisms of action, optimize formulation strategies, and evaluate its potential for clinical implementation in treating various ocular diseases.

## 6. Conclusions

The continuous evolution of ophthalmic drug delivery is largely driven by the need to overcome physiological and anatomical barriers while ensuring therapeutic efficacy, safety, and patient compliance. Permeation enhancers play a crucial role in facilitating drug absorption across corneal, conjunctival, and scleral barriers, particularly for hydrophilic and macromolecular drugs that inherently struggle to penetrate ocular tissues. Different classes of permeation enhancers have been developed and optimized, each offering distinct mechanisms of action, ranging from modulating tight junctions and lipid bilayers to enhancing drug solubility and bioavailability. While some enhancers are already well established in clinical formulations, others present emerging and promising alternatives with high potential for innovation in future drug delivery systems.

Among the most widely used and well-characterized permeation enhancers, cyclodextrins (CDs) have demonstrated significant advantages in ophthalmic formulations, particularly for anterior segment applications. Their ability to encapsulate poorly soluble drugs within a hydrophobic core enhances aqueous solubility, chemical stability, and overall bioavailability. Unlike many penetration enhancers, CDs do not disrupt cellular membranes or tight junctions, making them exceptionally safe and well tolerated. Their proven biocompatibility, low immunogenicity, and extensive toxicological validation give them a regulatory advantage over other enhancers. However, their intrinsic ability to facilitate posterior segment drug delivery is limited, necessitating their incorporation into advanced nanocarriers such as micelles and liposomes to achieve deeper tissue penetration. By combining CDs with lipid-based nanoparticles and stimuli-responsive carriers, drug release can be fine-tuned to respond to physiological triggers like pH changes, enzyme activity, and temperature fluctuations. Additionally, CDs can be used alongside physical enhancement techniques such as iontophoresis [534] and microneedling [535] to optimize drug delivery. Future advancements in this area are focusing on the integration of cyclodextrins with artificial-intelligence-driven formulation design, allowing for the optimization of CD–drug complexes tailored to an individual’s ocular physiology. Furthermore, their potential as carriers for gene therapies [536] and their inclusion in 3D-printed hydrogel contact lenses [537] could revolutionize sustained drug release strategies, particularly for chronic diseases such as glaucoma, diabetic retinopathy, and age-related macular degeneration. As ophthalmology embraces more sophisticated drug delivery strategies, cyclodextrin-based formulations will continue to drive innovation, enhancing treatment efficacy, safety, and patient outcomes.

In contrast, chelating agents such as EDTA and EGTA act by disrupting cadherin-mediated junctions through calcium depletion, significantly enhancing the permeability of hydrophilic drugs. While this mechanism has proven to be effective, its clinical application is limited by the associated risks of corneal swelling, inflammation, and potential endothelial damage. Moreover, their dual function as antimicrobial preservatives complicates their pharmacological profile, necessitating advanced delivery vehicles to modulate their activity safely. Advances in ophthalmic drug design are exploring chelating agents and nanotechnology-based chelating agent formulations that selectively target intercellular junctions without disrupting overall epithelial function, further improving their clinical applicability. The evolution of chelating-agent-based drug delivery will likely be driven by their integration into nanotechnology and smart drug delivery systems. Artificial-intelligence-driven formulation design could optimize their concentrations and combinations, ensuring maximum therapeutic benefits with minimal side effects. Their incorporation into innovative platforms, such as 3D-printed ocular drug delivery devices, has been explored as solubilizers and stabilizers in the phase of development of biomaterials [538]; however, there are no studies that report their use as permeation enhancers as part of the formulation, something that could be explored in the future. Their application in sustained-release inserts could further refine their application in ophthalmology.

Crown ethers have emerged as promising permeation enhancers in ophthalmic drug delivery due to their unique ability to selectively bind metal ions, particularly calcium, thereby modulating tight junction dynamics in the corneal epithelium. By facilitating transient junctional loosening, they enhance drug permeability without causing significant membrane disruption. Their amphiphilic nature further contributes to their effectiveness, enabling improved drug solubility and bioavailability. Despite these advantages, the clinical translation of crown ethers faces several challenges. Their rapid clearance from the ocular surface limits their retention time, necessitating formulation strategies that prolong their interaction with corneal tissues. Encapsulation within nanostructured lipid carriers, polymeric micelles, or mucoadhesive hydrogels could mitigate these concerns while maintaining their permeation-enhancing properties. Future advancements in ophthalmic drug delivery may combine crown ethers with other permeation enhancers or physical enhancement techniques, such as iontophoresis, to further optimize drug absorption. Their integration into smart drug delivery systems, including stimuli-responsive carriers and controlled-release implants, could revolutionize their application, allowing for sustained and targeted ocular therapy.

Among biopolymers, chitosan (CH) has gained increasing recognition as one of the most promising candidates for ophthalmic drug delivery due to its biocompatibility, biodegradability, and multifunctional properties. Its positively charged nature enables strong interactions with the negatively charged ocular epithelium, facilitating both transcellular and paracellular drug transport while ensuring prolonged retention on the ocular surface. Unlike most permeation enhancers, CH possesses inherent antimicrobial and anti-inflammatory properties, making it an attractive option for treating ocular infections and inflammatory conditions. However, its effectiveness depends on key structural attributes, such as molecular weight, degree of deacetylation, and solubility, necessitating precise formulation strategies to balance permeation enhancement with patient safety. To address challenges such as rapid elimination from the ocular surface and potential irritation, researchers are integrating CH into advanced drug delivery platforms, including nanoparticles, hydrogels, micelles, and liposomes, which enhance stability, prolong drug release, and improve therapeutic outcomes. The future of CH-based ophthalmic drug delivery lies in its synergy with other permeation enhancers and nanotechnology-driven carriers, which could refine drug penetration and controlled release mechanisms. Additionally, its role in emerging smart drug delivery systems, such as stimuli-responsive hydrogels [539] and bioadhesive ocular inserts [540], or 3D printing technology [541] holds great promise for achieving sustained targeted therapies. As ongoing research continues to push the boundaries of ophthalmic pharmacology, CH remains a frontrunner in the quest for safer, more effective, and longer-lasting ocular treatments.

Surface-acting agents (SAAs) have gained significant attention in ophthalmic drug delivery due to their ability to enhance drug solubility, permeability, and therapeutic efficacy. These compounds, which include non-ionic, cationic, and bile-salt-based surfactants, play a crucial role in improving drug retention and penetration through the ocular epithelium. Among them, polyoxyethylene alkyl derivatives (PADs) are particularly noteworthy for their biocompatibility and ability to improve systemic absorption, although further studies are needed to optimize their use for ophthalmic applications.

Polyoxyethylene sorbitan esters, such as Tween 80, have demonstrated significant potential in enhancing corneal drug permeation, making them valuable components in modern ophthalmic formulations. Likewise, sorbitan fatty acid esters (Spans), particularly Span 60, contribute to the development of nanovesicular delivery systems that improve drug retention and bioavailability. A particularly promising non-ionic surfactant, d-α-tocopheryl poly(ethylene glycol) 1000 succinate (VE-TPGS 1000), has been shown to enhance the permeability of drugs like riboflavin and brinzolamide while simultaneously inhibiting multidrug resistance proteins, reducing the risk of therapeutic inefficacy due to drug efflux mechanisms. Another widely investigated SAA is Labrasol, which enhances drug penetration by modulating tight junction integrity, thus facilitating greater corneal permeability when used at optimal concentrations. Similarly, N-methyl-2-pyrrolidone exhibits excellent solubilizing properties, yet its tolerability for ocular tissues remains an area of ongoing investigation. Lecithin, due to its biocompatibility and phospholipid structure, presents a particularly promising approach to enhancing drug retention and permeation while maintaining ocular safety. The advancement regarding SAAs in ophthalmic drug delivery lies in the development of sophisticated nanoformulations, hybrid delivery platforms, and intelligent drug carriers, which hold the potential to significantly enhance therapeutic efficacy. Recent technologies, including mucoadhesive nanoparticles, transferosomes, and lipid-based nanocarriers, present a promising avenue for improving drug bioavailability and patient adherence. Future research should focus on achieving an optimal balance between efficacy and safety by refining dosage strategies and incorporating biodegradable nanocarriers to ensure sustained controlled drug release. These innovations are poised to transform ophthalmic pharmacotherapy by minimizing the need for invasive procedures while maximizing therapeutic precision and patient outcomes.

CB, an alcohol-based preservative, has demonstrated significant permeation-enhancing capabilities with a lower toxicity profile than BAC, although concerns regarding corneal integrity remain. Meanwhile, CPC has shown promise in enhancing paracellular transport by disrupting intercellular junctions, improving corneal permeability. CX, a bisbiguanide antimicrobial agent, is highly effective against a broad range of pathogens and has been used in the treatment of Acanthamoeba keratitis. However, its potential for ocular toxicity at high concentrations suggests that further refinement in formulation strategies is necessary. To improve the safety and efficacy of cationic surfactants, future research is focusing on nanocarrier-based encapsulation, controlled-release systems, and bioadhesive formulations. Encapsulating these surfactants within liposomes, micelles, or polymeric nanoparticles may help to sustain drug release while reducing direct epithelial exposure, improving tolerability and safety. Additionally, the combination of cationic surfactants with biocompatible excipients could allow for lower concentrations while maintaining efficacy, thereby reducing toxicity concerns. These strategies are expected to lead to the development of safer and more effective ophthalmic therapeutics, enhancing drug bioavailability while preserving ocular surface integrity and improving patient comfort.

Bile salts, including sodium taurocholate, sodium deoxycholate, and sodium taurodeoxycholate, have emerged as promising permeation enhancers in ophthalmic drug delivery due to their ability to disrupt tight junctions and modify lipid membranes, thereby facilitating both paracellular and transcellular drug transport. Additionally, their solubilizing properties significantly improve the bioavailability of hydrophobic drugs, making them valuable components in advanced ocular formulations. However, their clinical applicability remains limited due to their potential to cause ocular irritation and compromise epithelial integrity at higher concentrations. To mitigate these drawbacks, formulation strategies such as bile-salt-loaded nanocarriers (liposomes, nanoemulsions, and micelles) are being explored to regulate drug release and minimize epithelial disruption. Furthermore, combining bile salts with mucoadhesive polymers may prolong drug retention on the ocular surface, optimizing therapeutic outcomes while minimizing irritation.

Glycosides, particularly saponins, digitonin, and escin, offer additional permeation-enhancing properties by interacting with lipid membranes and modulating epithelial permeability. Saponins, naturally occurring amphiphilic glycosides, can disrupt cell membranes to facilitate drug penetration while also providing anti-inflammatory and antimicrobial benefits. Similarly, digitonin enhances drug transport by forming cholesterol complexes in cellular membranes, while escin exhibits vascular protective effects, improving microcirculation and enhancing drug absorption. Despite these advantages, toxicity and irritation at higher concentrations remain key limitations of glycosides in ophthalmic formulations. To mitigate these issues, future advancements should focus on dose optimization and encapsulation within biocompatible carriers, such as liposomes or polymeric nanoparticles, to ensure controlled release and minimize ocular irritation. Their multifunctionality as both permeation enhancers and bioactive compounds makes glycosides a valuable tool in ophthalmic drug delivery, with careful formulation strategies paving the way for safer, more effective treatments.

Azone (1-dodecylazacycloheptan-2-one) is a well-established lipophilic penetration enhancer that has shown promise in ophthalmic drug delivery due to its ability to disrupt lipid bilayers and fluidize cell membranes, thereby facilitating transcorneal drug permeation. Its amphiphilic structure enables it to integrate into both hydrophilic and lipophilic environments, making it particularly effective for enhancing the absorption of poorly permeable drugs. However, azone’s high potency requires careful concentration control as excessive use may lead to ocular irritation and membrane destabilization. To maximize its potential while minimizing adverse effects, modern formulation strategies should focus on incorporating azone into nanocarriers, such as liposomes, nanoemulsions, or micelles, allowing for controlled drug release and reduced toxicity. By refining its application, azone holds significant promise in enhancing ocular drug delivery, particularly for challenging hydrophobic compounds, while ensuring safety and patient tolerability.

Cell-penetrating peptides (CPPs) represent a cutting-edge approach in ophthalmic drug delivery, offering a means to facilitate transcorneal and intracellular drug transport without significantly disrupting cellular integrity. Among them, Penetratin (PNT), a peptide derived from the Antennapedia homeodomain, enhances drug uptake through receptor-independent endocytosis and direct membrane translocation. The Trans-Activator of Transcription (TAT) protein transduction domain, derived from the HIV-1 virus, has demonstrated exceptional efficiency in crossing ocular barriers, making it a valuable tool for delivering macromolecules such as peptides and nucleic acids. Polyarginines, particularly those with eight or more arginine residues (R8 or R9), leverage electrostatic interactions with negatively charged cell membranes to improve drug permeability. Pep-1, a fusion peptide, facilitates the cytosolic delivery of hydrophilic and hydrophobic drugs while maintaining cell viability. Despite their high permeability potential, CPPs face challenges related to enzymatic degradation, potential toxicity, and specificity. Looking ahead, the future of CPP-based ophthalmic drug delivery will likely be driven by efforts to improve peptide stability and optimize targeted delivery. One of the most pressing concerns is the susceptibility of CPPs to enzymatic degradation, which significantly reduces their half-life in physiological environments. To address this, research is exploring chemical modifications such as peptide cyclization [542], D-amino acid substitution [543], and conjugation with nanoparticles, all of which have been shown to enhance resistance to enzymatic breakdown while maintaining peptide efficacy. Furthermore, developing strategies to increase the specificity of CPPs for ocular tissues is an important area of ongoing research. By engineering CPPs to recognize and bind to specific receptors on corneal or retinal cells, it may be possible to improve drug targeting while reducing off-target effects. Advances in nanotechnology and peptide engineering could also facilitate the development of multifunctional CPPs that combine permeation-enhancing properties with controlled drug release, offering a more sophisticated and precise approach to ophthalmic treatment. Research into mucoadhesive drug delivery systems and stimuli-responsive peptide carriers may further improve ocular retention and absorption, prolonging therapeutic effects while minimizing systemic exposure. Despite these promising advancements, the widespread clinical adoption of CPPs in ophthalmology faces several key limitations. One of the most critical concerns is toxicity as certain CPPs have been found to disrupt cellular membranes at high concentrations, leading to unintended cytotoxic effects. While many studies suggest that the majority of CPPs exhibit low toxicity at therapeutic doses, further in vivo research is needed to assess their long-term safety, particularly for chronic ocular diseases requiring repeated administration. Immunogenicity is another potential challenge as repeated exposure to exogenous peptides may trigger unwanted immune responses. Efforts to reduce immunogenicity through rational peptide design and surface modifications will be essential in ensuring their clinical viability. Additionally, the large-scale manufacturing and standardization of CPP-based therapeutics remain an obstacle to commercialization. Peptide synthesis is often costly and complex, requiring precise control over peptide length, sequence, and modifications to ensure consistent biological activity. While challenges remain, the continuous refinement of CPP technology holds immense potential for transforming ophthalmic pharmacotherapy. By improving peptide stability, enhancing targeting mechanisms, and developing innovative delivery systems, CPPs could provide a non-invasive, highly effective alternative to traditional ophthalmic treatments.

Cytochalasins are fungal-derived metabolites known for their ability to modulate actin cytoskeletal dynamics, leading to the transient opening of tight junctions in epithelial cells. This mechanism enhances paracellular drug permeation, making cytochalasins potential candidates for improving ocular drug delivery. Cytochalasin B has been shown to primarily affect the junctional portion of the corneal epithelium, facilitating increased drug transport without significant cytotoxicity when used at controlled concentrations. Despite their promising permeability-enhancing effects, concerns regarding cytoskeletal integrity and epithelial stability remain. Future advancements should explore cytochalasin-loaded nanoparticles or hybrid biomaterials to ensure targeted and sustained release, minimizing adverse effects. Additionally, mechanistic studies on their impact on corneal and conjunctival tight junction modulation could lead to novel strategies for enhancing ophthalmic drug bioavailability, particularly for hydrophilic molecules that rely on paracellular pathways.

Finally, terpenes such as borneol and menthol have attracted attention for their ability to enhance ocular drug permeability while providing additional pharmacological benefits. Borneol facilitates transcorneal drug penetration by disrupting lipid bilayers and transiently opening tight junctions, making it particularly effective for hydrophilic drug delivery. Similarly, menthol enhances drug permeability through its interaction with transient receptor potential melastatin-8 (TRPM8) channels, which also contribute to its soothing and tear-stimulating properties. However, their volatility, potential irritation, and formulation stability remain concerns that need to be addressed. Recent advancements have focused on encapsulating terpenes in nanocarriers such as liposomes, nanoemulsions, and polymeric micelles to ensure controlled release and prolonged ocular retention. The future of terpene-based ophthalmic drug delivery lies in the integration of these compounds into hybrid drug delivery systems, combining their permeability-enhancing properties with advanced excipients for optimized therapeutic efficacy.

Cyclodextrins, chitosan, and surface-acting agents currently stand out as the most established due to their well-characterized safety profiles, regulatory approval, and broad applicability in ophthalmic formulations. Cyclodextrins, particularly HP-β-CD and SBE-β-CD, are highly valued for their ability to solubilize hydrophobic drugs without disrupting cellular integrity, making them indispensable in aqueous formulations. Chitosan, with its strong mucoadhesive properties and capacity to transiently open tight junctions, offers prolonged drug retention and enhanced permeability, making it a preferred choice for sustained-release ocular systems. Surface-acting agents, including lecithin and polyoxyethylene derivatives, further optimize drug solubility and penetration, particularly when integrated into lipid-based nanocarriers. However, while these enhancers remain the gold standard, emerging candidates such as cell-penetrating peptides, bile salts, and terpenes hold significant promise for the future. CPPs, with their unparalleled ability to transport macromolecules intracellularly, represent a transformative approach, particularly for gene- and protein-based ophthalmic therapies. Bile salts, leveraging their dual role as solubilizers and membrane permeability enhancers, present an opportunity for improving drug absorption in both anterior and posterior segment drug delivery. Terpenes, particularly borneol and menthol, not only enhance permeability but also offer additional pharmacological benefits, such as anti-inflammatory effects, positioning them as multifunctional excipients. All these emerging compounds offer promising avenues for further development.

In general, despite the remarkable potential of modified nanotechnology-based ocular drug delivery systems, several critical limitations hinder their widespread clinical application. Formulation stability remains a significant challenge as nanoparticles and nanocarriers must maintain structural integrity over extended periods while ensuring consistent drug release profiles. Moreover, large-scale manufacturing and reproducibility pose technical and economic barriers as variations in nanoparticle synthesis can impact batch-to-batch consistency, regulatory approval, and cost-effectiveness. Regulatory hurdles further complicate clinical translation as nanocarrier-based formulations often require extensive safety evaluations and long-term biocompatibility studies. Additionally, ocular clearance mechanisms, such as tear turnover, blinking, and enzymatic degradation, can limit the residence time of nanocarriers on the ocular surface, necessitating even more advanced formulation strategies to enhance bioavailability. While innovations in mucoadhesive polymers, stimuli-responsive nanocarriers, and targeted drug delivery approaches continue to address these challenges, further research is needed to optimize their safety, scalability, and clinical feasibility before these systems can be fully integrated into routine ophthalmic care.

As the field advances, the convergence of biotechnology, material science, and computational modeling will unlock unprecedented possibilities, transforming the way ocular diseases are treated. The fusion of biomimetic nanocarriers, stimuli-responsive drug systems, nanotechnology-driven formulations, hybrid drug carriers, gene-editing therapeutics, and AI-assisted drug design will not only optimize drug penetration but also enable personalized medicine, tailoring treatments to individual patient needs with unparalleled precision and redefining the boundaries of what is possible in ophthalmic pharmacotherapy. The era of invasive, repetitive ocular drug administration is approaching obsolescence, giving way to a future where a single intelligently designed formulation can achieve sustained targeted therapy with minimal side effects. The future is not merely about refining drug delivery; it is about orchestrating a revolution in vision science, where sight is no longer passively preserved but actively restored, where once-incurable ocular diseases become manageable, and where the barriers of pharmacological limitations are shattered via the integration of nanotechnology, biomimetic engineering, gene therapy, and artificial intelligence.

## Figures and Tables

**Table 1:** Main ocular barriers.

Layer/Component		Description	Function	Key Components	References
Precorneal Tear Film (PTF)	Tear Film Lipid Layer (TFLL)	Composed of meibomian gland secretions (polar and nonpolar lipids); forms the anterior layer of PTF, coating the aqueous layer as a continuous sheet spread by blinking.	The main function is to reduce PTF surface tension, but it also provides smooth optical surface, enhances stabilization, and aids the spreading of the PTF, delays aqueous layer evaporation, prevents tear spillover, and seals lid margins during sleep.	Glycerophospholipids, sphingophospholipids, u-hydroxy fatty acids, wax esters, cholesterol esters, triglycerides, free fatty acids, hydrocarbons.	[15,16,17,18]
	Aqueous Middle Layer	Comprises 90% of PTF. Mainly produced by the lacrimal and by the third eyelid glands.	Lubricates ocular surface, washes away debris, nourishes the avascular cornea (oxygen, glucose, proteins, and salts), and provides antibacterial, antifungal, antiparasitic, and antiviral protection.	Water, electrolytes (sodium, potassium, calcium, magnesium, etc.), lysozyme, lactoferrin, lipocalin, secretory IgA, cytokines, vitamins, and peptide growth factors.	[19,20,21,22,23]
	Mucous Layer	Present in the apical epithelial ocular surface. Merges with the aqueous middle layer.	Protects against pathogens, offers an interface between tear film and ocular surface, helps the hydration of the ocular surface, and maintains tear film stability.	High-molecular-weight O-linked glycoproteins (Mucins).	[24,25]
Cornea	Epithelium	The most superficial layers are flat with apical microvilli covered by a glycocalyx, which maximizes the surface area contacting the mucinous layer of the tear film.	Forms the first anatomical line of defense, offering a highly resistant barrier against foreign substances and preventing the transcellular and paracellular transport of many drugs, mainly due to its lipophilic character and the existence of strong intercellular junctions.	It is characterized as a non-keratinizing stratified epithelium consisting of three layers: the stratum superficiale (superficial nonkeratinized stratified squamous cells), the stratum intermedius (wing cells), and the stratum basale (the basal cell layer)	[11,26,27,28]
	Stroma	Constitutes 90% of the cornea’s volume.	Provides structural corneal integrity and transparency.	Highly ordered Type I and V collagen fibrils aligned in lamellae, proteoglycans (e.g., lumican, keratocan, and mimecan) that regulate water retention and collagen organization, keratocytes that maintain the extracellular matrix, monocytes, and dendritic cells.	[29,30,31,32]
	Descemet’s Membrane	Elastic and acellular structure that anchors the corneal endothelium to the corneal stroma.	Regulates the bidirectional flow of nutrients, growth factors, cytokines, and macromolecules between the aqueous humor and stroma.	Type IV collagen and laminin, produced by endothelial cells.	[32,33]
	Endothelium	Hexagonal-shaped lipophilic monolayer of cells that represents the innermost sheet of the cornea, forming a barrier between the stroma and aqueous humor.	Maintains corneal transparency by actively transporting ions and passively regulating water movement to keep the stroma in a relative dehydrated state.	Endothelial cells.	[6,34,35]
Conjunctiva	Epithelium	Outer layer of conjunctiva	Provides a barrier function and assists in tear film adherence and spreading through microvilli. Goblet cells produce mucin.	Epithelial cells and goblet cells.	[36,37,38]
	Submucosal Lamina Propria (Stroma)	Underlying connective tissue layer supporting the conjunctival epithelium.	Provides structural integrity and contains blood and lymphatic vessels.	Connective tissue, blood vessels, and lymphatic vessels.	[36,39]
Sclera			Maintains intraocular pressure, protects intraocular contents, prevents deformations caused by muscle contractions, and reduces internal light scattering to ensure optimal retinal imaging.	Dense Types I (>90%), III, V, and VI collagen bundles irregularly interspersed with elastic fibers and microfibrils in a hydrated matrix of proteoglycans (the water content of sclera is around 68%), glycoproteins, and elastin, interspersed with fibroblasts.	[40,41,42,43,44]

**Table 2:** Main marketed ophthalmic formulations, including CDs.

Drug	Cyclodextrin	Trade Name	Company
Chloramphenicol	RM-β-CD	Clorocil^®^	Oftalder (Lisbon, Portugal)
Diclofenac	HPγ-CD	Voltaren^®^/ Voltarol^®^	Novartis (Basel, Schwitzerland)
Indomethacin	HP-β-CD	Indocid^®^/ Indocyllir^®^	Baush & Lomb (Bridgewater, NJ, USA)
Naphasoline hydrochloride	β-CD	Clear eyes^®^	Prestige Consumer Healthcare Inc. (Tarrytown, NY, USA)
Thimerosal	β-CD	Vitaseptol^®^	Novartis (Basel, Schwitzerland)

**Table 3:** Main studies on corneal permeation enhancer properties of CDs.

Cyclodextrin	Drug Model	Permeation Outcomes	Adverse Reactions	References
SBE-β-CD	Cyclosporine A	Enhanced corneal permeation, improved tear volume, reduced inflammation, and better dry eye management.	Not mentioned.	[131]
HP-γ-CD	Spironolactone	Superior therapeutic effects in corneal wound healing compared to potassium canrenoate.	Well-tolerated.	[123]
HP-β-CD	Ketoconazole	Increased precorneal retention, corneal permeation, and ocular bioavailability using ion-sensitive gel.	Well-tolerated.	[126]
β-CD	Itraconazole	Improved ocular kinetics with dissolving microneedle system	Well-tolerated.	[125]
SBE-β-CD	Fluconazole	Faster drug delivery and higher bioavailability using ocular patch system.	Well-tolerated.	[124]
γ-CD	Vitamin A palmitate	Faster recovery of dry eye disease using crosslinked γ-CD framework.	Well-tolerated.	[129]
β-CD	Hesperidin	Sustained release and enhanced corneal permeation using in situ gel.	Well-tolerated.	[144]
HP-β-CD	Vorinostat	Increased corneal and conjunctival bioavailability compared to suspension.	Well-tolerated.	[122]
HP-β-CD	Nepafenac	Improved corneal permeability and bioavailability compared to suspension eye drop.	Not mentioned.	[111]
2-HP-β-CD	Triamcinolone acetonide	Increased retention time, prolonged release, and enhanced corneal permeability using PLGA nanoparticles.	Not mentioned.	[120]
β-CD	Dexamethasone	Reduced vessel area in the cornea using ROS-responsive nanogel with β-CD (once-daily) compared to free drug solution (twice-daily)	Well-tolerated.	[121]
SBE-β-CD	Dexamethasone	Prolonged residence time and enhanced permeability using chitosan/SBE-β-CD nanoparticles.	Well-tolerated.	[119]
γ-CD	Dexamethasone	Improved bioavailability and anti-inflammatory effect using nanoemulsion-based pseudopolyrotaxane hydrogel.	Well-tolerated.	[118]

**Table 4:** Main studies on corneal permeation enhancer properties of crown ethers.

Crown Ether Concentration	Permeation Outcomes	Adverse Reactions	References
12C4, 15C5, and 18C6 1 mg/mL and 30 mg/mL	Enhanced calcium sequestration and riboflavin permeability in bovine corneas; 12C4 showed highest efficiency.	12C4, 15C5, and 18C6 induced similar epithelial changes. Similar epithelial changes. Less toxic than BAC > 0.1% or 1 mg/mL EDTA. 12C4 was the least toxic.	[171]
18C6 1 mM	Enhanced corneal permeability of phenytoin sodium and promoted healing of alkali-induced corneal ulcers.	Minimal irritation and safe ophthalmic use.	[178]

**Table 5:** Main studies on corneal permeation enhancer properties of VE-TPGS 1000.

VE-TPGS 1000 Concentration	Permeation Outcomes	Adverse Reactions	References
0.01–1% (*w*/*w*)	Increased riboflavin and chlorhexidine permeability, peaking at 0.5%. There was no added benefit at 1%.	No irritation reported.	[300,303]
1%	Brinzolamide-loaded nanoliposomes containing TPGS (T-LPs/Brz) showed 5-fold higher permeation than Brz alone and reduced IOP for 4–10 h.	T-LPs/Brz caused mild conjunctival inflammation. The corneal epithelium retained an intact structure.	[299]
0.01–1% (*w*/*w*)	Enhanced the accumulation of chlorhexidine gluconate in the cornea in a concentration-dependent manner up to 0.5% (*w*/*w*).	No treatment-related alterations or irritation reported.	[303]

**Table 6:** Main studies on corneal permeation enhancer properties of Labrasol^®^.

Labrasol^®^ Concentration	Permeation Outcomes	Adverse Reactions	References
0.5%, 2% and 8% (*w*/*v*)	Enhanced fluorescein sodium permeation in a concentration-dependent manner	Not mentioned.	[306]
0.5–5.0% (*v*/*v*)	Increased corneal permeability of baicalin. The highest improvement was seen at 2%.	Non-irritant up to 3%. Slightly irritant at 5%.	[308,310]
4.5% (*w*/*w*)	The formulated Labrasol containing ribavirin microemulsion improved corneal permeability 3-fold compared to aqueous solution.	No irritation or allergic reaction observed.	[309]

**Table 7:** Main studies on corneal permeation enhancer properties of N-methyl-2-pyrrolidone (NMP).

NMP Concentration	Permeation Outcomes	Adverse Reactions	References
0.1–10% (*w*/*w*)	No significant enhancement for timolol maleate or acyclovir.	Non-irritant up to 10%. NMP 10% significantly increased percentage hydration level.	[311]
2.5%, 5% and 10% (*v*/*v*)	Improved corneal permeability of ibuprofen, ribavirin, puerarin, and enoxacin in a concentration-dependent manner, with maximum at 10%.	Non-irritant at concentrations of 0–10% (*v*/*v*), slightly irritant at 15% (*v*/*v*), and moderately irritant at 20% (*v*/*v*).	[312]

**Table 8:** Main studies on corneal permeation enhancer properties of BS.

BS Concentration	Permeation Outcomes	Adverse Reactions	References
GCA and CA 1% (*w*/*v*)	Not mentioned.	Caused mild irritation in a concentration-dependent manner.	[262]
DCA, TCA, UDC, TDC 0.05%	TDC enhanced the in vitro corneal permeation of atenolol. DCA and UDC enhanced the in vitro corneal permeation of timolol maleate and betaxolol hydrochloride.	DCA showed higher toxicity.	[337]
GCA, DCA and TCA	Improved permeability of liposomes compared to cholesterol-containing liposomes.	DCA showed greater toxicity.	[410]
GCA and TCA 0.5%	Enhanced permeability of PCL-PF68 nanoparticles in cornea, iris, and ciliary body.	Not mentioned.	[413]

**Table 9:** Main studies on corneal permeation enhancer properties of SP.

SP Concentration	Permeation Outcomes	Adverse Reactions	References
0.5% and 1%	Not mentioned.	Significant corneal damage (>30%).	[262]
0.01–1%	SP 0.05% increased permeability of atenolol and timolol maleate and, to a smaller extent, betaxolol hydrochloride.	SP 0.015% caused corneal hydration to increase. SP 0.25% caused slight irritation.	[337]
0.5%	Enhanced corneal permeability of atenolol, carteolol, and tilisolol.	Slight ocular irritation after 12 h.	[423]
0.5%	Enhanced corneal permeability of FITC-dextran-4 and FITC-dextran-10.	Not mentioned.	[425]
0.5%	Enhanced corneal permeability of Thyrotropin-releasing Hormone and Luteinizing-Hormone-releasing Hormone in a molecular-weight-dependent manner.	Not mentioned.	[426]
30 mg/mL	SP micelles improved diclofenac permeation with enhanced anti-inflammatory effects.	No significant ocular irritation.	[427]

**Table 10:** Main studies on corneal permeation enhancer properties of DG.

DG Concentration	Permeation Outcomes	Adverse Reactions	References
0.0025% (*w*/*v*)	Slightly increased timolol maleate and betaxolol permeability.	DG 0.0025% increased the corneal hydration value. Concentrations > 0.015% caused significant swelling and opacification of the cornea.	[337]
1 mM	Increased corneal permeation of PEG 200–1000 in a molecular-weight-dependent manner.	Severe corneal epithelial damage.	[436]
0.1 mM	Had no effect as a corneal permeation enhancer for monovalent single-chain variable region (scFv) antibody fragments and divalent miniantibodies.	Not mentioned.	[437]

**Table 11:** Main studies on corneal permeation enhancer properties of escin.

Escin Concentration	Permeation Outcomes	Adverse Reactions	References
0.05% (*w*/*v*)	Enhanced corneal permeability of atenolol, timolol, and levobunolol.	Increased corneal hydration. Showed a similar irritation profile as digitonin	[337]
0.015%	Increased corneal permeability of timolol maleate.	Not mentioned.	[445]

**Table 12:** Main studies on corneal permeation enhancer properties of azone.

Azone Concentration	Permeation Outcomes	Adverse Reactions	References
1%	Enhanced ex vivo corneal permeation and in vivo anti-inflammatory efficiency compared to the azone-free formulations.	No signs of ocular irritancy.	[451]
0.1%	Enhanced the corneal penetration of hydrophilic compounds (acetazolamide, sulfacetamide, guanethidine and cimetidine).	No in vivo irritating signs.	[452]
5%	Enhanced cyclosporine corneal permeation, leading to faster steady state.	No toxicity to corneal epithelium.	[453]
0.1%, 0.4% and 0.7%	Increased the in vivo ocular bioavailability of cimetidine 3.9- and 22-fold, respectively. Above 0.1%, did not produce higher ocular permeation	Exerted toxic and irritating effects on the cornea in a concentration-dependent manner. The highest dose caused moderate congestion.	[454]
0.2%	Improved permeability of Gd-DTPA.	No corneal toxicity.	[455]
0.025%	No significant effect on levobunolol corneal permeability.	Not mentioned.	[456]
0.125–0.625%	Increased corneal permeability of S-timolol maleate (STM).	No signs of ophthalmic irritation.	[457]
0.125–0.625%	increased the in vitro corneal permeation of Latanoprost in a concentration-dependent manner up 0.5%.	No signs of ophthalmic irritation.	[458]

**Table 14:** Main studies on corneal permeation enhancer properties of cytochalasins.

Cytochalasin Concentration	Permeation Outcomes	Adverse Reactions	References
Cytochalasin B 0.1–1 mM	Reduced transcorneal electrical resistance in a concentration-dependent manner. The effect of cytochalasin B was predominantly on the junctional portion of the corneal epithelium.	From all permeation enhancers tested (EDTA, digitonin, and deoxycholic acid), cytochalasin B appeared to present the least harmful effects to the corneal tissue.	[412]
Cytochalasin B 1 mM	Improved corneal permeation of PEG 400 to PEG 700. Above the molecular weight of PEG 700, there was no significant change.	Not mentioned.	[436]
Cytochalasin B 10 μg/mL	Enhanced inulin corneal permeation but had no effect on glucose or epinephrine.	Not mentioned.	[524]
Cytochalasin B 3 nM	Increased in vivo gene delivery into ocular tissues by eye drops of plasmid/poly(ethylene oxide)-poly(propylene oxide)-poly(ethylene oxide) (Pluronic^®^ P105) polymeric micelles.	Not mentioned.	[525]

**Table 15:** Main studies on corneal permeation enhancer properties of borneol.

Borneol Concentration	Outcomes	Adverse Reactions	References
0.05% and 0.1%	0.05% and 0.1% had no effect on the fluconazole corneal permeability. Higher and significant corneal-permeation-enhancing effects when combined with menthol.	No significant differences in corneal hydration levels, in vivo irritation, and blinking frequency.	[89]
0.05% and 0.1%	The optimal penetration-enhancing concentration of borneol was 0.1%. The penetration-enhancing effects of borneol were increased when used in combination with Labrasol.	The maximum safe concentration of borneol was 0.2%. No obvious corneal or iris irritation symptoms occurred when using 0.1% borneol.	[308]
0.025%, 0.05% and 0.1%	Improved the penetration of puerarin eye drops. 0.1% led to an increase in timolol maleate eye drop corneal permeability when compared to the control group.	No impact on corneal hydration.	[526]
0.05%, 0.1% and 0.2%	Improved the corneal permeability of rhodamine B, sodium fluorescein, and FITC-dextrans of 4 kDa in a borneol-concentration-dependent manner. The paracellular permeability was correlated to the molecular weight of the drugs tested.	No damage was observed to the epithelium or endothelium. Ocular irritation tests indicated good corneal biocompatibility.	[527]
0.01%, 0.02% and 0.04%	Increased the corneal permeability of geniposide. This enhancing effect was temporary and closely dose-related.	All formulations were well tolerated, and no ocular damage or clinically abnormal signs were observed.	[528]
0.1%	Both natural and synthetic borneol increased corneal permeability of dexamethasone, ribavirin, and tobramycin.	No ocular irritation observed. No damage to the corneal epithelium was found. Neither toxic nor inflammatory reactions were found.	[529]
0.04%	Increased the concentrations of the seven alkaloids studied in the aqueous humor.	Not mentioned.	[530]

**Table 16:** Main studies on corneal permeation enhancer properties of menthol.

Menthol Concentration	Outcomes	Adverse Reactions	References
0.05% and 0.1%	Enhanced fluconazole permeation. Higher effects when combined with borneol.	No irritation, no impact on corneal hydration.	[89]
0.1% and 0.2%	The optimal penetration-enhancing concentration of menthol was found to be 0.2%. The penetration-enhancing effects of menthol were increased when used in combination with Labrasol.	The maximum safe concentration of menthol was 0.4%. There was no significant tissue irritation or damage.	[308]
0.025%, 0.05% and 0.1%	Increased permeability of dexamethasone disodium phosphate.	Mild irritation, resolved in 48 h.	[531]
0.1%, 0.2%, and 0.3%.	Improved natamycin penetration under iontophoresis.	The maximum safe concentration of menthol was 0.4%. There was no significant tissue damage.	[533]

## References

[1] World Health Organization (2019). World Report on Vision.

[2] Hughes P.M., Olejnik O., Chang-Lin J.E., Wilson C.G. (2005). Topical and systemic drug delivery to the posterior segments. Adv. Drug Deliv. Rev..

[3] Le Bourlais C., Acar L., Zia H., Sado P.A., Needham T., Leverge R. (1997). Ophthalmic Drug Delivery Systems—Recent Advances. Prog. Retin. Eye Res..

[4] Patel A., Cholkar K., Agrahari V., Mitra A.K. (2013). Ocular drug delivery systems—An overview. World J. Pharmacol..

[5] Morrison P.W.J., Khutoryanskiy V.V. (2014). Anatomy of the Eye and the Role of Ocular Mucosa in Drug Delivery. Mucoadhesive Mater. Drug Deliv. Syst..

[6] Gaudana R., Ananthula H.K., Parenky A., Mitra A.K. (2010). Ocular drug delivery. AAPS J..

[7] Lin S., Ge C., Wang D., Xie Q., Wu B., Wang J., Nan K., Zheng Q., Chen W. (2019). Overcoming the Anatomical and Physiological Barriers in Topical Eye Surface Medication Using a Peptide-Decorated Polymeric Micelle. ACS Appl. Mater. Interfaces.

[8] Hamalainen K.M., Kananen K., Auriola S., Kontturi K., Urtti A. (1997). Characterization of Paracellular and Aqueous Penetration Routes in Cornea, Conjunctiva, and Sclera. Investig. Ophthalmol. Vis. Sci..

[9] Lee V.H.L., Robinson J.R. (1979). Mechanistic and Quantitative Evaluation of Precorneal Pilocarpine Disposition in Albino Rabbits. J. Pharm. Sci..

[10] Morrison P.W.J., Khutoryanskiy V.V. (2014). Advances in ophthalmic drug delivery. Ther. Deliv..

[11] Moiseev R.V., Morrison P.W.J., Steele F., Khutoryanskiy V.V. (2019). Penetration enhancers in ocular drug delivery. Pharmaceutics.

[12] Liu R., Liu Z., Zhang C., Zhang B. (2011). Gelucire44/14 as a novel absorption enhancer for drugs with different hydrophilicities: In vitro and in vivo improvement on transcorneal permeation. J. Pharm. Sci..

[13] Kaur I.P., Smitha R. (2002). Penetration Enhancers and Ocular Bioadhesives: Two New Avenues for Ophthalmic Drug Delivery. Drug Dev. Ind. Pharm..

[14] Thareja A., Hughes H., Alvarez-Lorenzo C., Hakkarainen J.J., Ahmed Z. (2021). Penetration enhancers for topical drug delivery to the ocular posterior segment—A systematic review. Pharmaceutics.

[15] Pucker A.D., Nichols J.J. (2012). Analysis of Meibum and Tear Lipids. Ocul. Surf..

[16] Bron A.J., Tiffany J.M., Gouveia S.M., Yokoi N., Voon L.W. (2004). Functional Aspects of the Tear Film Lipid Layer. Exp. Eye Res..

[17] Cwiklik L. (2016). Tear film lipid layer: A molecular level view. Biochim. Biophys. Acta Biomembr..

[18] Foulks G.N., Bron A.J. (2003). Meibomian gland dysfunction: A clinical scheme for description, diagnosis, classification, and grading. Ocul. Surf..

[19] Sun X. (2023). Mechanism of Tear Electrolytes Concentration in Tear Formation on Ocular Surface. J. Eye Dis. Disord..

[20] Stahl U., Willcox M., Stapleton F. (2012). Osmolality and tear film dynamics. Clin. Exp. Optom..

[21] Zhou L., Beuerman R.W. (2012). Tear analysis in ocular surface diseases. Prog. Retin. Eye Res..

[22] Berlutti F., Pantanella F., Natalizi T., Frioni A., Paesano R., Polimeni A., Valenti P. (2011). Antiviral Properties of Lactoferrin—A Natural Immunity Molecule. Molecules.

[23] Zhang H., Fu G., Zhang D. (2014). Cloning, characterization, and production of a novel lysozyme by different expression hosts. J. Microbiol. Biotechnol..

[24] Willcox M.D.P., Argüeso P., Georgiev G.A., Holopainen J.M., Laurie G.W., Millar T.J., Papas E.B., Rolland J.P., Schmidt T.A., Stahl U. (2017). TFOS DEWS II Tear Film Report. Ocul. Surf..

[25] Hattrup C.L., Gendler S.J. (2008). Structure and function of the cell surface (tethered) mucins. Annu. Rev. Physiol..

[26] Nautscher N., Bauer A., Steffl M., Amselgruber W.M. (2016). Comparative morphological evaluation of domestic animal cornea. Vet. Ophthalmol..

[27] Eghrari A.O., Riazuddin S.A., Gottsch J.D. (2015). Overview of the Cornea: Structure, Function, and Development. Progress in Molecular Biology and Translational Science.

[28] Yi X.-J., Wang Y., Yu F.-S.X. (2000). Corneal Epithelial Tight Junctions and Their Response to Lipopolysaccharide Challenge. Investig. Ophthalmol. Vis. Sci..

[29] Møller-Pedersen T. (2004). Keratocyte reflectivity and corneal haze. Exp. Eye Res..

[30] Hassell J.R., Birk D.E. (2010). The molecular basis of corneal transparency. Exp. Eye Res..

[31] Fini M.E., Stramer B.M. (2005). How the Cornea Heals: Cornea-Specific Repair Mechanisms Affecting Surgical Outcomes. Cornea.

[32] Sridhar M.S. (2018). Anatomy of cornea and ocular surface. Indian J. Ophthalmol..

[33] de Oliveira R.C., Wilson S.E. (2020). Descemet’s membrane development, structure, function and regeneration. Exp. Eye Res..

[34] Barar J., Javadzadeh A.R., Omidi Y. (2008). Ocular novel drug delivery: Impacts of membranes and barriers. Expert Opin. Drug Deliv..

[35] Tuft S.J., Coster D.J. (1990). The Corneal Endothelium. Eye.

[36] Pellegrini G., Golisano O., Paterna P., Lambiase A., Bonini S., Rama P., De Luca M. (1999). Location and Clonal Analysis of Stem Cells and Their Differentiated Progeny in the Human Ocular Surface. J. Cell Biol..

[37] Nichols B.A. (1996). Conjunctiva. Microsc. Res. Tech..

[38] Rusciano G., Zito G., Pesce G., Del Prete S., Cennamo G., Sasso A. (2016). Assessment of conjunctival microvilli abnormality by micro-Raman analysis. J. Biophotonics.

[39] Hornof M., Toropainen E., Urtti A. (2005). Cell culture models of the ocular barriers. Eur. J. Pharm. Biopharm..

[40] Keeley F.W., Morin J.D., Vesely S. (1984). Characterization of Collagen from Normal Human Sclera. Exp. Eye Res..

[41] Meek K.M. (2008). The cornea and sclera. Collagen: Structure and Mechanics.

[42] Ross R., Bornstein P. (1971). Elastic Fibers in the Body. Sci. Am..

[43] You J., Willcox M.D., Madigan M.C., Wasinger V., Schiller B., Walsh B.J., Graham P.H., Kearsley J.H., Li Y. (2013). Tear Fluid Protein Biomarkers. Advances in Clinical Chemistry.

[44] Watson P.G., Young R.D. (2004). Scleral structure, organisation and disease. A review. Exp. Eye Res..

[45] Holly F.J., Lemp M.A. (1977). Tear Physiology and Dry Eyes. Surv. Ophthalmol..

[46] Davis K., Carter R., Tully T., Negulescu I., Storey E. (2015). Comparative evaluation of aqueous humor viscosity. Vet. Ophthalmol..

[47] Spreull J.S.A. (1966). Symposium: The Corneal Ulcer*-I Anatomy and Physiology of the Cornea of the Dog. J. Small Anim. Pract..

[48] Labelle P. (2017). The Eye. Pathologic Basis of Veterinary Disease Expert Consult.

[49] Wilson S.E. (2020). Bowman’s layer in the cornea- structure and function and regeneration. Exp. Eye Res..

[50] Gukasyan H.J., Kim K.-J., Lee V.H. (2008). The Conjunctival Barrier in Ocular Drug Delivery. Drug Absorption Studies: In Situ, In Vitro and In Silico Models.

[51] Downie L.E., Bandlitz S., Bergmanson J.P.G., Craig J.P., Dutta D., Maldonado-Codina C., Ngo W., Siddireddy J.S., Wolffsohn J.S. (2021). CLEAR—Anatomy and physiology of the anterior eye. Contact Lens Anterior Eye.

[52] Boote C., Sigal I.A., Grytz R., Hua Y., Nguyen T.D., Girard M.J.A. (2020). Scleral structure and biomechanics. Prog. Retin. Eye Res..

[53] Maliborski A., Różycki R. (2014). Diagnostic imaging of the nasolacrimal drainage system. Part I. Radiological anatomy of lacrimal pathways. Physiology of tear secretion and tear outflow. Med. Sci. Monit..

[54] Ludwig A. (2005). The use of mucoadhesive polymers in ocular drug delivery. Adv. Drug Deliv. Rev..

[55] Nagataki S., Mishima S. (1980). Pharmacokinetics of instilled drugs in the human eye. Int. Ophthalmol. Clin..

[56] Mishima S., Gasset A., Klyce S.D., Baum J.L. (1966). Determination of tear volume and tear flow. Investig. Ophthalmol..

[57] Takahashi Y., Kakizaki H., Nakano T., Asamoto K., Ichinose A., Iwaki M. (2011). Anatomy of the vertical lacrimal canaliculus and lacrimal punctum: A macroscopic study. Ophthalmic Plast. Reconstr. Surg..

[58] Tucker N.A., Tucker S.M., Linberg J.V. (1996). The Anatomy of the Common Canaliculus. Arch. Ophthalmol..

[59] Mouly S., Mahé I., Haouchine B., Sanson-Le-Pors M.J., Blain P., Tillet Y., Dewailly J., Mongold J.J., Bergmann J.F. (2006). Pharmacodynamics of a new ophthalmic mydriatic insert in healthy volunteers: Potential alternative as drug delivery system prior to cataract surgery. Basic Clin. Pharmacol. Toxicol..

[60] Ghate D., Edelhauser H.F. (2008). Barriers to Glaucoma Drug Delivery. J. Glaucoma.

[61] Maurice D.M. (1980). Factors influencing the penetration of topically applied drugs. Int. Ophthalmol. Clin..

[62] Karki R., Meena M., Prakash T., Rajeswari T., Goli D., Kumar S. (2011). Reduction in drop size of ophthalmic topical drop preparations and the impact of treatment. J. Adv. Pharm. Technol. Res..

[63] Shell J.W. (1982). Pharmacokinetics of topically applied ophthalmic drugs. Surv. Ophthalmol..

[64] Agrahari V., Mandal A., Agrahari V., Trinh H.M., Joseph M., Ray A., Hadji H., Mitra R., Pal D., Mitra A.K. (2016). A comprehensive insight on ocular pharmacokinetics. Drug Deliv. Transl. Res..

[65] Patton T.F., Robinson J.R. (1976). Quantitative Precorneal Disposition of Topically Applied Pilocarpine Nitrate in Rabbit Eyes. J. Pharm. Sci..

[66] Chrai S.S., Makoid M.C., Eriksen S.P., Robinson J.R. (1974). Drop Size and Initial Dosing Frequency Problems of Topically Applied Ophthalmic Drugs. J. Pharm. Sci..

[67] Van Santvliet L., Ludwig A. (2004). Determinants of eye drop size. Surv. Ophthalmol..

[68] Abelson M.B., Udell I.J., Weston J.H. (1981). Normal Human Tear pH by Direct Measurement. Arch. Ophthalmol..

[69] Beckwith-Cohen B., Elad D., Bdolah-Abram T., Ofri R. (2014). Comparison of tear pH in dogs, horses, and cattle. Vet. Ophthalmol..

[70] Fletcher E.L., Brennan F.A. (1993). The effect of solution tonicity on the eye. Clin. Exp. Optom..

[71] Maurice D. (1971). The Tonicity of an Eye Drop and Its Dilution by Tears. Exp. Eye Res..

[72] Green-Church K.B., Nichols K.K., Kleinholz N.M., Zhang L., Nichols J.J. (2008). Investigation of the human tear film proteome using multiple proteomic approaches. Mol. Vis..

[73] Kim D.W., Lee S.H., Ku S.K., Cho S.H., Cho S.W., Yoon G.H., Hwang H.S., Park J., Eum W.S., Kwon O.S. (2013). Transduced PEP-1-FK506BP ameliorates corneal injury in Botulinum toxin A-induced dry eye mouse model. BMB Rep..

[74] Toropainen E., Ranta V.P., Vellonen K.S., Palmgrén J., Talvitie A., Laavola M., Suhonen P., Hämäläinen K.M., Auriola S., Urtti A. (2003). Paracellular and passive transcellular permeability in immortalized human corneal epithelial cell culture model. Eur. J. Pharm. Sci..

[75] Ranta V.P., Toropainen E., Talvitie A., Auriola S., Urtti A. (2002). Simultaneous determination of eight β-blockers by gradient high-performance liquid chromatography with combined ultraviolet and fluorescence detection in corneal permeability studies in vitro. J. Chromatogr. B.

[76] Toropainen E., Ranta V.P., Talvitie A., Suhonen P., Urtti A. (2001). Culture Model of Human Corneal Epithelium for Prediction of Ocular Drug Absorption. Investig. Ophthalmol. Vis. Sci..

[77] Khurana V., Vadlapudi A.D., Vadlapatla R.K., Pal D., Mitra A.K. (2015). Functional characterization and molecular identification of Vitamin C transporter (SVCT2) in human corneal epithelial (HCEC) and retinal pigment epithelial (D407) cells. Curr. Eye Res..

[78] Zhou X., Li X., Xu J., Cheng Y., Cao F. (2021). Latanoprost-loaded cyclodextrin microaggregate suspension eye drops for enhanced bioavailability and stability. Eur. J. Pharm. Sci..

[79] Kawazu K., Yamada K., Nakamura M., Ota A. (2000). Characterization of Cyclosporin A Transport in Cultured Rabbit Corneal Epithelial Cells: P-Glycoprotein Transport Activity and Binding to Cyclophilin. Investig. Ophthalmol. Vis. Sci..

[80] Kawazu K., Shiono H., Tanioka H., Ota A., Ikuse T., Takashina H., Kawashima Y. (1998). Beta adrenergic antagonist permeation across cultured rabbit corneal epithelial cells grown on permeable supports. Curr. Eye Res..

[81] Kawazu K., Midori Y., Ota A. (1999). Cultured Rabbit Corneal Epithelium Elicits Levofloxacin Absorption and Secretion. J. Pharm. Pharmacol..

[82] Enríquez-de-Salamanca A., Calder V., Gao J., Galatowicz G., García-Vázquez C., Fernández I., Stern M.E., Diebold Y., Calonge M. (2008). Cytokine responses by conjunctival epithelial cells: An in vitro model of ocular inflammation. Cytokine.

[83] Civiale C., Paladino G., Marino C., Trombetta F., Pulvirenti T., Enea V. (2003). Multilayer primary epithelial cell culture from bovine conjunctiva as a model for in vitro toxicity tests. Ophthalmic Res..

[84] Palumbo P., Picchini U., Beck B., Van Gelder J., Delbar N., DeGaetano A. (2008). A general approach to the apparent permeability index. J. Pharmacokinet. Pharmacodyn..

[85] Resende A.P., Silva B., Braz B.S., Nunes T., Gonçalves L., Delgado E. (2017). Ex vivo permeation of erythropoietin through porcine conjunctiva, cornea, and sclera. Drug Deliv. Transl. Res..

[86] Srinivasan B., Kolli A.R., Esch M.B., Abaci H.E., Shuler M.L., Hickman J.J. (2015). TEER Measurement Techniques for In Vitro Barrier Model Systems. J. Lab. Autom..

[87] Powell D.W. (1981). Barrier function of epithelia. Am. J. Physiol..

[88] Silva B., Marto J., Braz B.S., Delgado E., Almeida A.J., Gonçalves L. (2020). New nanoparticles for topical ocular delivery of erythropoietin. Int. J. Pharm..

[89] Liu J., Fu S., Wei N., Hou Y., Zhang X., Cui H. (2012). The effects of combined menthol and borneol on fluconazole permeation through the cornea ex vivo. Eur. J. Pharmacol..

[90] Rasoanirina B.N.V., Lassoued M.A., Kamoun A., Bahloul B., Miladi K., Sfar S. (2020). Voriconazole-loaded self-nanoemulsifying drug delivery system (SNEDDS) to improve transcorneal permeability. Pharm. Dev. Technol..

[91] Bhosale V.A., Srivastava V., Valamla B., Yadav R., Singh S.B., Mehra N.K. (2022). Preparation and Evaluation of Modified Chitosan Nanoparticles using Anionic Sodium Alginate Polymer for Treatment of Ocular Disease. Pharmaceutics.

[92] Pawar P.K., Majumdar D.K. (2006). Effect of Formulation Factors on In Vitro Permeation of Moxifloxacin from Aqueous Drops Through Excised Goat, Sheep, and Buffalo Corneas. AAPS PharmSciTech.

[93] Barbalho G.N., Falcão M.A., Lopes J.M.S., Lopes J.M., Contarato J.L.A., Gelfuso G.M., Cunha-Filho M., Gratieri T. (2023). Dynamic Ex Vivo Porcine Eye Model to Measure Ophthalmic Drug Penetration Under Simulated Lacrimal Flow. Pharmaceutics.

[94] Zeiss C.J. (2013). Translational models of ocular disease. Vet. Ophthalmol..

[95] del Amo E.M., Urtti A. (2015). Rabbit as an animal model for intravitreal pharmacokinetics: Clinical predictability and quality of the published data. Exp. Eye Res..

[96] Zernii E.Y., Baksheeva V.E., Iomdina E.N., Averina O.A., Permyakov S.E., Philippov P.P., Zamyatnin A.A., Senin I.I. (2016). Rabbit Models of Ocular Diseases: New Relevance for Classical Approaches. CNS Neurol. Disord. Drug Targets.

[97] Bouhenni R.A., Dunmire J., Sewell A., Edward D.P. (2012). Animal models of glaucoma. J. Biomed. Biotechnol..

[98] Shimizu S., Ochiai Y., Kamijima K., Takai N., Watanabe S., Aihara M. (2024). Development and characterization of a chronic high intraocular pressure model in New Zealand white rabbits for glaucoma research. Exp. Eye Res..

[99] Chen W.L., Lin C.T., Lin N.T., Tu I.H., Li J.W., Chow L.P., Liu K.R., Hu F.R. (2009). Subconjunctival injection of bevacizumab (Avastin) on corneal neovascularization in different rabbit models of corneal angiogenesis. Investig. Ophthalmol. Vis. Sci..

[100] Chen J., Ding X., Du W., Tang X., Yu W.Z. (2021). Inhibition of corneal neovascularization by topical application of nintedanib in rabbit models. Int. J. Ophthalmol..

[101] Ashton P. (2015). Huge Therapeutic Advances: Bigger Drug Delivery Opportunities. Ophthalmic Drug Deliv..

[102] Irimia T., Ghica M.V., Popa L., Anuţa V., Arsene A.L., Dinu-Pîrvu C.E. (2018). Strategies for improving ocular drug bioavailability and corneal wound healing with chitosan-based delivery systems. Polymers.

[103] Rupenthal I.D. (2015). Ocular Drug Delivery Technologies: Exciting Times Ahead. Ophthalmic Drug Deliv..

[104] Kali G., Haddadzadegan S., Bernkop-Schnürch A. (2024). Cyclodextrins and derivatives in drug delivery: New developments, relevant clinical trials, and advanced products. Carbohydr. Polym..

[105] Loftsson T., Stefánsson E. (1997). Effect of Cyclodextrins on Topical Drug Delivery to the Eye. Drug Dev. Ind. Pharm..

[106] Loftsson T., Brewster M.E. (1996). Pharmaceutical Applications of Cyclodextrins. 1. Drug Solubilization and Stabilization. J. Pharm. Sci..

[107] Rajewski R.A., Stella V.J. (1996). Pharmaceutical Applications of Cyclodextrins. 2. In Vivo Drug Delivery. J. Pharm. Sci..

[108] Loftsson T., Jarho P., Másson M., Järvinen T. (2005). Cyclodextrins in drug delivery. Expert Opin. Drug Deliv..

[109] Cal K., Centkowska K. (2008). Use of cyclodextrins in topical formulations: Practical aspects. Eur. J. Pharm. Biopharm..

[110] Rowe R.C., Sheskey P.J., Owen S.C. (2006). Handbook of Pharmaceutical Excipients.

[111] Vincze A., Facskó R., Budai-Szűcs M., Katona G., Gyarmati B., Csorba A., Zelkó R., Nagy Z.Z., Szente L., Balogh G.T. (2023). Cyclodextrin-enabled nepafenac eye drops with improved absorption open a new therapeutic window. Carbohydr. Polym..

[112] Chaudhari P., Ghate V.M., Lewis S.A. (2019). Supramolecular cyclodextrin complex: Diversity, safety, and applications in ocular therapeutics. Exp. Eye Res..

[113] Soe H.M.S.H., Maw P.D., Loftsson T., Jansook P. (2022). A Current Overview of Cyclodextrin-Based Nanocarriers for Enhanced Antifungal Delivery. Pharmaceuticals.

[114] Stefánsson E., Loftsson T. (2010). Microspheres and nanotechnology for drug delivery. Retinal Pharmacotherapy.

[115] Soe H.M.S.H., Kerdpol K., Rungrotmongkol T., Pruksakorn P., Autthateinchai R., Wet-osot S., Loftsson T., Jansook P. (2023). Voriconazole Eye Drops: Enhanced Solubility and Stability through Ternary Voriconazole/Sulfobutyl Ether β-Cyclodextrin/Polyvinyl Alcohol Complexes. Int. J. Mol. Sci..

[116] Frijlink H.W., Eissens A.C., Schoonen A.J.M., Lerk C.F. (1990). The effects of cyclodextrins on drug absorption II. In vivo observations. Int. J. Pharm..

[117] Loftsson T., Stefánsson E. (2022). Aqueous eye drops containing drug/cyclodextrin nanoparticles deliver therapeutic drug concentrations to both anterior and posterior segment. Acta Ophthalmol..

[118] Fang G., Zhao R., Zhu L., Wang Q., Peng S., Kang L., Lu H., Zhang G., Tang B. (2025). Nanoemulsion-based pseudopolyrotaxane hydrogel for enhanced corneal bioavailability and treatment of corneal inflammation. J. Control. Release.

[119] Racaniello G.F., Balenzano G., Arduino I., Iacobazzi R.M., Lopalco A., Lopedota A.A., Sigurdsson H.H., Denora N. (2024). Chitosan and Anionic Solubility Enhancer Sulfobutylether-β-Cyclodextrin-Based Nanoparticles as Dexamethasone Ophthalmic Delivery System for Anti-Inflammatory Therapy. Pharmaceutics.

[120] Qin Z., Li B., Deng Q., Wen Y., Feng S., Duan C., Zhao B., Li H., Gao Y., Ban J. (2024). Polymer Nanoparticles with 2-HP-β-Cyclodextrin for Enhanced Retention of Uptake into HCE-T Cells. Molecules.

[121] Xiang Y., Qiu Z., Ding Y., Du M., Gao N., Cao H., Zuo H., Cheng H., Gao X., Zheng S. (2024). Dexamethasone-loaded ROS stimuli-responsive nanogels for topical ocular therapy of corneal neovascularization. J. Control. Release.

[122] Yang J., Ma Y., Luo Q., Liang Z., Lu P., Song F., Zhang Z., Zhou T., Zhang J. (2022). Improving the solubility of vorinostat using cyclodextrin inclusion complexes: The physicochemical characteristics, corneal permeability and ocular pharmacokinetics of the drug after topical application. Eur. J. Pharm. Sci..

[123] Rodrigues-Braz D., Zhu L., Gélizé E., Clarin J.P., Chatagnon X., Benzine Y., Rampignon P., Thouvenin A., Bourges J.L., Behar-Cohen F. (2023). Spironolactone Eyedrop Favors Restoration of Corneal Integrity after Wound Healing in the Rat. Pharmaceuticals.

[124] Mahfufah U., Sya’ban Mahfud M.A., Saputra M.D., Abd Azis S.B., Salsabila A., Asri R.M., Habibie H., Sari Y., Yulianty R., Alsayed A.R. (2024). Incorporation of Inclusion Complexes in the Dissolvable Microneedle Ocular Patch System for the Efficiency of Fluconazole in the Therapy of Fungal Keratitis. ACS Appl. Mater. Interfaces.

[125] Putri R.A., Enggi C.K., Sulistiawati S., Burhanuddin H., Iskandar I.W., Saputra R.R., Rahman L., Sartini S., Rifai Y., Aswad M. (2024). Development of itraconazole ocular delivery system using β-cyclodextrin complexation incorporated into dissolving microneedles for potential improvement treatment of fungal keratitis. J. Biomater. Sci. Polym. Ed..

[126] Xia H., Yang J., Song F., Pu G., Dong F., Liang Z., Zhang J. (2024). Development of ion-triggered in situ gel containing ketoconazole/hydroxypropyl-β-cyclodextrin for ocular delivery: In vitro and in vivo evaluation. Drug Deliv..

[127] Gözcü S., Polat H.K., Gültekin Y., Ünal S., Karakuyu N.F., Şafak E.K., Doğan O., Pezik E., Haydar M.K., Aytekin E. (2024). Formulation of hesperidin-loaded in situ gel for ocular drug delivery: A comprehensive study. J. Sci. Food Agric..

[128] Farkas E., Abboud H., Nagy N., Hofmeister B., Ostorházi E., Tóth B., Pinke B., Mészáros L., Zelkó R., Kazsoki A. (2024). Formulation and Development of Nanofiber-Based Ophthalmic Insert for the Treatment of Bacterial Conjunctivitis. Int. J. Mol. Sci..

[129] Ye X., Li F., Li M., Zhang G., Wang W., Wang Z., Zhang H., Dong L., Lin X., Wu L. (2024). Controlled release of vitamin A palmitate from crosslinked cyclodextrin organic framework for dry eye disease therapy. Int. J. Pharm..

[130] Zhang F., Tan M., Hu Z.E., Zhang Y.T., Qi X.W., Che Y.T., Li J., Zhang S., Li B.J. (2025). A hyaluronic acid-modified cyclodextrin self-assembly system for the delivery of β-carotene in the treatment of dry eye disease. Int. J. Biol. Macromol..

[131] Chaudhari P., Birangal S., Mavlankar N., Pal A., Mallela L.S., Roy S., Kodoth A.K., Ghate V., Nampoothiri M., Lewis S.A. (2022). Oil-free eye drops containing Cyclosporine A/cyclodextrin/PVA supramolecular complex as a treatment modality for dry eye disease. Carbohydr. Polym..

[132] Wang T.Z., Guan B., Liu X.X., Ke L.N., Wang J.J., Nan K.H. (2022). A topical fluorometholone nanoformulation fabricated under aqueous condition for the treatment of dry eye. Colloids Surf. B Biointerfaces.

[133] Tanito M., Hara K., Takai Y., Matsuoka Y., Nishimura N., Jansook P., Loftsson T., Stefánsson E., Ohira A. (2011). Topical Dexamethasone-Cyclodextrin Microparticle Eye Drops for Diabetic Macular Edema Preparation of Dexamethasone-Cyclodextrin Microparticle Eye Drops. Investig. Ophthalmol. Vis. Sci..

[134] Ohira A., Hara K., Jóhannesson G., Tanito M., Ásgrímsdóttir G.M., Lund S.H., Loftsson T., Stefánsson E. (2015). Topical dexamethasone γ-cyclodextrin nanoparticle eye drops increase visual acuity and decrease macular thickness in diabetic macular oedema. Acta Ophthalmol..

[135] Shulman S., Jõhannesson G., Stefánsson E., Loewenstein A., Rosenblatt A., Habot-Wilner Z. (2015). Topical dexamethasone-cyclodextrin nanoparticle eye drops for non-infectious Uveitic macular oedema and vitritis—A pilot study. Acta Ophthalmol..

[136] Loftsson T., Hreinsdóttir D., Stefánsson E. (2010). Cyclodextrin microparticles for drug delivery to the posterior segment of the eye: Aqueous dexamethasone eye drops. J. Pharm. Pharmacol..

[137] Yang L., Jonas J.B., Wei W. (2014). Central serous chorioretinopathy and bright light: Authors reply. Acta Ophthalmol..

[138] Johannsdottir S., Jansook P., Stefansson E., Kristinsdottir I.M., Fulop Z., Asgrimsdottir G.M., Thorsteindsottir M., Eiriksson F.F., Loftsson T. (2018). Topical drug delivery to the posterior segment of the eye: Dexamethasone concentrations in various eye tissues after topical administration for up to 15 days to rabbits. J. Drug Deliv. Sci. Technol..

[139] Lu J., Zhu X., Zhang M., Jiang X., Guo W., Jiang F., Cao F. (2023). In vitro and in vivo assessment of structural integrity for HPCD complex@Liposome nanocomposites from ocular surface to the posterior segment of the eye. Carbohydr. Polym..

[140] Zhu X., Li S., Huang J., Yin C., Li Y., Guo W., Jiang F., Cao F. (2025). FRET-based analysis on the fate of liposome and cyclodextrin@liposome nanocomposites from ocular surface to the posterior segment of the eye. J. Control. Release.

[141] Khin S.Y., Soe H.M.S.H., Chansriniyom C., Pornputtapong N., Asasutjarit R., Loftsson T., Jansook P. (2022). Development of Fenofibrate/Randomly Methylated β-Cyclodextrin-Loaded Eudragit^®^ RL 100 Nanoparticles for Ocular Delivery. Molecules.

[142] Lorenzo-Soler L., Praphanwittaya P., Olafsdottir O.B., Kristinsdottir I.M., Asgrimsdottir G.M., Loftsson T., Stefansson E. (2022). Topical noninvasive retinal drug delivery of a tyrosine kinase inhibitor: 3% cediranib maleate cyclodextrin nanoparticle eye drops in the rabbit eye. Acta Ophthalmol..

[143] Alambiaga-Caravaca A.M., Cantó A., Rodilla V., Miranda M., López-Castellano A. (2022). Topical Ocular Administration of Progesterone Decreases Photoreceptor Cell Death in Retinal Degeneration Slow (rds) Mice. Pharmaceuticals.

[144] Higashi T., Goto T., Onodera R., Hirotsu T., Ikeda H.O., Motoyama K. (2024). Sustained Release Formulation of Hydroxypropyl-β-cyclodextrin Eye Drops Using Xanthan Gum. Chem. Pharm. Bull..

[145] Finnegan S., Percival S.L. (2015). EDTA: An Antimicrobial and Antibiofilm Agent for Use in Wound Care. Adv. Wound Care.

[146] Ghaffarieh A., Ciolino J.B. (2021). Potential of Application of Iron Chelating Agents in Ophthalmic Diseases. Semin. Ophthalmol..

[147] Meldolesi J., Castiglioni G., Parma R., Nassivera N., De P. (1978). Ca++-Dependent Disassembly and Reassembly of Occluding Junctions in Guinea Pig Pancreatic Acinar Cells Effect of Drugs. J. Cell Biol..

[148] Cereijido M., Robbins S., Dolan W.J., Rotunno C.A., Sabatini D.D. (1978). Polarized Monolayers Formed by Epithelial Cells on a Permeable and Translucent Support. J. Cell Biol..

[149] Martinez-Palomo A., Meza I., Beaty G., Cereijido M. (1980). Experimental Modulation of Occluding Junctions in a Cultured Transporting Epithelium. J. Cell Biol..

[150] Gonzalez-Mariscal L., Chávez De Ramírez B., Cereijido M. (1985). Tight Junction Formation in Cultured Epithelial Cells (MDCK). J. Membr. Biol..

[151] Ma T.Y., Tran D., Hoa N., Nguyen D., Merryfield M., Tarnawski A. (2000). Mechanism of Extracellular Calcium Regulation of Intestinal Epithelial Tight Junction Permeability: Role of Cytoskeletal Involvement. Microcirculation.

[152] Klingler C., Kniesel U., Bamforth S.D., Wolburg H., Engelhardt B., Risau W. (2000). Disruption of epithelial tight junctions is prevented by cyclic nucleotide-dependent protein kinase inhibitors. Histochem. Cell Biol..

[153] Overduin M., Harvey T.S., Bagby S., Tong K.I., Yau P., Takeichi M., Ikura M. (1995). Solution structure of the epithelial cadherin domain responsible for selective cell adhesion. Science.

[154] Contreras-Ruiz L., Schulze U., García-Posadas L., Arranz-Valsero I., López-García A., Paulsen F., Diebold Y. (2012). Structural and functional alteration of corneal epithelial barrier under inflammatory conditions. Curr. Eye Res..

[155] Meng W., Takeichi M. (2009). Adherens junction: Molecular architecture and regulation. Cold Spring Harb. Perspect. Biol..

[156] Ramachandran C., Srinivas S.P. (2010). Formation and disassembly of adherens and tight junctions in the corneal endothelium: Regulation by actomyosin contraction. Investig. Ophthalmol. Vis. Sci..

[157] Morrison P.W.J., Khutoryanskiy V.V. (2014). Enhancement in corneal permeability of riboflavin using calcium sequestering compounds. Int. J. Pharm..

[158] Rodriguez I., Antonio Vázquez J., Pastrana L., Khutoryanskiy V.V. (2017). Enhancement and inhibition effects on the corneal permeability of timolol maleate: Polymers, cyclodextrins and chelating agents. Int. J. Pharm..

[159] Ahuja M., Shridhar A., Kanti D. (2006). Effect of Formulation Factors on In Vitro Permeation of Diclofenac from Experimental and Marketed Aqueous Eye Drops Through Excised Goat Cornea. Yakugaku Zasshi.

[160] Malhotra M., Majumdar D.K. (2002). Effect of preservative, antioxidant and viscolizing agents on in vitro transcorneal permeation of ketorolac tromethamine. J. Pharm. Pharmacol..

[161] Kikuchi T., Suzuki M., Kusai A., Iseki K., Sasaki H. (2005). Synergistic effect of EDTA and boric acid on corneal penetration of CS-088. Int. J. Pharm..

[162] Malhotra S., Khare A., Grover K., Singh I., Pawar P. (2014). Design and Evaluation of Voriconazole Eye Drops for the Treatment of Fungal Keratitis. J. Pharm..

[163] Scholz M., Chang Lin J.-E., Lee V.H.L., Keipert S. (2002). Pilocarpine Permeability Across Ocular Tissues and Cell Cultures: Influence of Formulation Parameters. Pharm. Res..

[164] Pescina S., Carra F., Padula C., Santi P., Nicoli S. (2016). Effect of pH and penetration enhancers on cysteamine stability and trans-corneal transport. Eur. J. Pharm. Biopharm..

[165] Chetoni P., Burgalassi S., Monti D., Saettone M.F. (2003). Ocular toxicity of some corneal penetration enhancers evaluated by electrophysiology measurements on isolated rabbit corneas. Toxicol. Vitr..

[166] Malhotra M., Majumdar D.K. (2005). In Vivo Ocular Availability of Ketorolac Following Ocular Instillations of Aqueous, Oil, and Ointment Formulations to Normal Corneas of Rabbits: A Technical Note. AAPS PharmSciTech.

[167] Epstein S.P., Ahdoot M., Marcus E., Asbell P.A. (2009). Comparative toxicity of preservatives on immortalized corneal and conjunctival epithelial cells. J. Ocul. Pharmacol. Ther..

[168] Grass G.M., Robinson J.R. (1988). Mechanisms of Corneal Drug Penetration II: Ultrastructural Analysis of Potential Pathways for Drug Movement. J. Pharm. Sci..

[169] Grass G.M., Wood R.W., Robinson J.R. (1985). Effects of calcium chelating agents on corneal permeability. Investig. Ophthalmol. Vis. Sci..

[170] Kralj M., Tušek-Božić L., Frkanec L. (2008). Biomedical potentials of crown ethers: Prospective antitumor agents. ChemMedChem.

[171] Morrison P.W.J., Porfiryeva N.N., Chahal S., Salakhov I.A., Lacourt C., Semina I.I., Moustafine R.I., Khutoryanskiy V.V. (2017). Crown Ethers: Novel Permeability Enhancers for Ocular Drug Delivery?. Mol. Pharm..

[172] Steed J.W. (2001). First-and second-sphere coordination chemistry of alkali metal crown ether complexes. Coord. Chem. Rev..

[173] Davis F., Higson S. (2011). Crown Ethers, Cryptands and Other Compounds. Macrocycles.

[174] Marjanović M., Kralj M., Supek F., Frkanec L., Piantanida I., Šmuc T., Tušek-Božić L. (2007). Antitumor potential of crown ethers: Structure-activity relationships, cell cycle disturbances, and cell death studies of a series of ionophores. J. Med. Chem..

[175] Song M.Z., Zhu L.Y., Gao X.K., Dou J.M., Sun D.Z. (2005). Microcalorimetric study on host-guest complexation of naphtho-15-crown-5 with four ions of alkaline earth metal. J. Zhejiang Univ. Sci. B.

[176] Ullah F., Khan T.A., Iltaf J., Anwar S., Khan M.F.A., Khan M.R., Ullah S., Rehman M.F.U., Mustaqeem M., Kotwica-Mojzych K. (2022). Heterocyclic Crown Ethers with Potential Biological and Pharmacological Properties: From Synthesis to Applications. Appl. Sci..

[177] Chehardoli G., Bahmani A. (2019). The role of crown ethers in drug delivery. Supramol. Chem..

[178] Mahmoud D.B., Afifi S.A., El Sayed N.S. (2020). Crown ether nanovesicles (crownsomes) repositioned phenytoin for healing of corneal ulcers. Mol. Pharm..

[179] Guibal E. (2005). Heterogeneous catalysis on chitosan-based materials: A review. Prog. Polym. Sci..

[180] Nagpal K., Singh S.K., Mishra D.N. (2010). Chitosan Nanoparticles: A Promising System in Novel Drug Delivery. Chem. Pharm. Bull..

[181] Agnihotri S.A., Mallikarjuna N.N., Aminabhavi T.M. (2004). Recent advances on chitosan-based micro- and nanoparticles in drug delivery. J. Control. Release.

[182] Domard A., Chatelet C., Damour O., Domard A. (2001). Influence of the degree of acetylation on some biological properties of chitosan films. Biomaterials.

[183] Herdiana Y., Wathoni N., Shamsuddin S., Muchtaridi M. (2022). Drug release study of the chitosan-based nanoparticles. Heliyon.

[184] Wang L.Y., Ma G.H., Su Z.G. (2005). Preparation of uniform sized chitosan microspheres by membrane emulsification technique and application as a carrier of protein drug. J. Control. Release.

[185] Zhu X., Su M., Tang S., Wang L., Liang X., Meng F., Hong Y., Xu Z. (2012). Synthesis of thiolated chitosan and preparation nanoparticles with sodium alginate for ocular drug delivery. Mol. Pharm..

[186] Ricci F., Racaniello G.F., Lopedota A., Laquintana V., Arduino I., Lopalco A., Cutrignelli A., Franco M., Sigurdsson H.H., Denora N. (2022). Chitosan/sulfobutylether-β-cyclodextrin based nanoparticles coated with thiolated hyaluronic acid for indomethacin ophthalmic delivery. Int. J. Pharm..

[187] Li J., Jin X., Yang Y., Zhang L., Liu R., Li Z. (2020). Trimethyl chitosan nanoparticles for ocular baicalein delivery: Preparation, optimization, in vitro evaluation, in vivo pharmacokinetic study and molecular dynamics simulation. Int. J. Biol. Macromol..

[188] Shinde U.A., Joshi P.N., Jain D.D., Singh K. (2019). Preparation and Evaluation of N-Trimethyl Chitosan Nanoparticles of Flurbiprofen for Ocular Delivery. Curr. Eye Res..

[189] Asasutjarit R., Theerachayanan T., Kewsuwan P., Veeranodha S., Fuongfuchat A., Ritthidej G.C. (2015). Development and Evaluation of Diclofenac Sodium Loaded-N-Trimethyl Chitosan Nanoparticles for Ophthalmic Use. AAPS PharmSciTech.

[190] Alhowyan A.A., Kalam M.A., Iqbal M., Raish M., El-Toni A.M., Alkholief M., Almomen A.A., Alshamsan A. (2023). Mesoporous Silica Nanoparticles Coated with Carboxymethyl Chitosan for 5-Fluorouracil Ocular Delivery: Characterization, In Vitro and In Vivo Studies. Molecules.

[191] Silva B., Gonçalves L.M., Braz B.S., Delgado E. (2023). Topical Administration of a Nanoformulation of Chitosan-Hyaluronic Acid-Epoetin Beta in a Rat Model of Glaucoma. Pharmaceuticals.

[192] Chhonker Y.S., Prasad Y.D., Chandasana H., Vishvkarma A., Mitra K., Shukla P.K., Bhatta R.S. (2015). Amphotericin-B entrapped lecithin/chitosan nanoparticles for prolonged ocular application. Int. J. Biol. Macromol..

[193] Alkholief M., Kalam M.A., Raish M., Ansari M.A., Alsaleh N.B., Almomen A., Ali R., Alshamsan A. (2023). Topical Sustained-Release Dexamethasone-Loaded Chitosan Nanoparticles: Assessment of Drug Delivery Efficiency in a Rabbit Model of Endotoxin-Induced Uveitis. Pharmaceutics.

[194] Rubenicia A.M.L., Cubillan L.D.P., Sicam V.A.D.P., Macabeo A.P.G., Villaflores O.B., Castillo A.L. (2021). Intraocular pressure reduction effect of 0.005% latanoprost eye drops in a hyaluronic acid-chitosan nanoparticle drug delivery system in albino rabbits. Transl. Vis. Sci. Technol..

[195] Li N., Zhao Z., Ma H., Liu Y., Nwafor E.O., Zhu S., Jia L., Pang X., Han Z., Tian B. (2022). Optimization and Characterization of Low-Molecular-Weight Chitosan-Coated Baicalin mPEG-PLGA Nanoparticles for the Treatment of Cataract. Mol. Pharm..

[196] Abdullah T., Ibrahim N., Warsi M. (2016). Chondroitin sulfate-chitosan nanoparticles for ocular delivery of bromfenac sodium: Improved permeation, retention, and penetration. Int. J. Pharm. Investig..

[197] Schipper N.G.M., Olsson S., Hoogstraate J.A., deBoer A.G., Varum K.M., Artursson P. (1997). Chitosans as Absorption Enhancers for Poorly Absorbable Drugs 2: Mechanism of Absorption Enhancement. Pharm. Res..

[198] Pratap-Singh A., Guo Y., Baldelli A., Singh A. (2023). Mercaptonicotinic acid activated thiolated chitosan (MNA-TG-chitosan) to enable peptide oral delivery by opening cell tight junctions and enhancing transepithelial transport. Sci. Rep..

[199] Vllasaliu D., Exposito-Harris R., Heras A., Casettari L., Garnett M., Illum L., Stolnik S. (2010). Tight junction modulation by chitosan nanoparticles: Comparison with chitosan solution. Int. J. Pharm..

[200] Wang S., Gao Z., Liu L., Li M., Zuo A., Guo J. (2022). Preparation, in vitro and in vivo evaluation of chitosan-sodium alginate-ethyl cellulose polyelectrolyte film as a novel buccal mucosal delivery vehicle. Eur. J. Pharm. Sci..

[201] Dodane V., Amin Khan M., Merwin J.R. (1999). Effect of chitosan on epithelial permeability and structure. Int. J. Pharm..

[202] Shi L., Li Z., Liang Z., Zhang J., Liu R., Chu D., Han L., Zhu L., Shen J., Li J. (2022). A dual-functional chitosan derivative platform for fungal keratitis. Carbohydr. Polym..

[203] Fu T., Yi J., Lv S., Zhang B. (2017). Ocular amphotericin B delivery by chitosan-modified nanostructured lipid carriers for fungal keratitis-targeted therapy. J. Liposome Res..

[204] Liu Y., Cui X., Zhao L., Zhang W., Zhu S., Ma J. (2021). Chitosan Nanoparticles to Enhance the Inhibitory Effect of Natamycin on Candida Albicans. J. Nanomater..

[205] Sanap S.N., Bisen A.C., Kedar A., Yadav K.S., Krishna A., Akhir A., Chopra S., Mugale M.N., Bhatta R.S. (2022). Chitosan/HPMC-based mucoadhesive film co-loaded with fluconazole and ofloxacin for management of polymicrobial keratitis. Int. J. Biol. Macromol..

[206] Gao N., Ju X., Jiao X., Qi Y., Tian Y., Jiang S., Niu Z., Zhao S., Yang R. (2024). Breaking Down the Barriers of Drug Resistance and Corneal Permeability with Chitosan-Poly(ethylene glycol)-LK13 Peptide Conjugate to Combat Fungal Keratitis. ACS Infect. Dis..

[207] Sun X., Sheng Y., Li K., Sai S., Feng J., Li Y., Zhang J., Han J., Tian B. (2022). Mucoadhesive phenylboronic acid conjugated chitosan oligosaccharide-vitamin E copolymer for topical ocular delivery of voriconazole: Synthesis, in vitro/vivo evaluation, and mechanism. Acta Biomater..

[208] Cui X., Li X., Xu Z., Guan X., Ma J., Ding D., Zhang W. (2021). Fabrication and Characterization of Chitosan/Poly(Lactic-Co-Glycolic Acid) Core-Shell Nanoparticles by Coaxial Electrospray Technology for Dual Delivery of Natamycin and Clotrimazole. Front. Bioeng. Biotechnol..

[209] Latifi A., Esmaeili F., Mohebali M., Yasami-Khiabani S., Rezaeian M., Soleimani M., Kazemirad E., Amani A. (2024). Chitosan nanoparticles improve the effectivity of miltefosine against Acanthamoeba. PLoS Negl. Trop. Dis..

[210] Padaga S.G., Ch S., Paul M., Wable B.D., Ghosh B., Biswas S. (2024). Chitosan oligosaccharide/pluronic F127 micelles exhibiting anti-biofilm effect to treat bacterial keratitis. Carbohydr. Polym..

[211] Padaga S.G., Bhatt H., Ch S., Paul M., Itoo A.M., Ghosh B., Roy S., Biswas S. (2024). Glycol Chitosan-Poly(lactic acid) Conjugate Nanoparticles Encapsulating Ciprofloxacin: A Mucoadhesive, Antiquorum-Sensing, and Biofilm-Disrupting Treatment Modality for Bacterial Keratitis. ACS Appl. Mater. Interfaces.

[212] Ch S., Padaga S.G., Ghosh B., Roy S., Biswas S. (2023). Chitosan-poly(lactide-co-glycolide)/poloxamer mixed micelles as a mucoadhesive thermo-responsive moxifloxacin eye drop to improve treatment efficacy in bacterial keratitis. Carbohydr. Polym..

[213] Meng S., Hu H., Qiao Y., Wang F., Zhang B.N., Sun D., Zhou L., Zhao L., Xie L., Zhang H. (2023). A Versatile Hydrogel with Antibacterial and Sequential Drug-Releasing Capability for the Programmable Healing of Infectious Keratitis. ACS Nano.

[214] Chang Y.F., Cheng Y.H., Ko Y.C., Chiou S.H., Liu C.J. (2022). Development of topical chitosan/β-glycerophosphate-based hydrogel loaded with levofloxacin in the treatment of keratitis: An ex-vivo study. Heliyon.

[215] Lu Y., Geng W., Li L., Xie F., Zhang M., Xie H., Cai J. (2025). Enhanced antibacterial and antibiofilm activities of quaternized ultra-highly deacetylated chitosan against multidrug-resistant bacteria. Int. J. Biol. Macromol..

[216] Sikhondze S.S., Makoni P.A., Walker R.B., Khamanga S.M.M. (2023). Chitosan-Coated SLN: A Potential System for Ocular Delivery of Metronidazole. Pharmaceutics.

[217] Javed S., Abbas G., Shah S., Rasul A., Irfan M., Saleem A., Hosny K.M., Bukhary S.M., Safhi A.Y., Sabei F.Y. (2023). Tobramycin-loaded nanoparticles of thiolated chitosan for ocular drug delivery: Preparation, mucoadhesion and pharmacokinetic evaluation. Heliyon.

[218] De Gaetano F., Marino A., Marchetta A., Bongiorno C., Zagami R., Cristiano M.C., Paolino D., Pistarà V., Ventura C.A. (2021). Development of chitosan/cyclodextrin nanospheres for levofloxacin ocular delivery. Pharmaceutics.

[219] Kalam M.A., Iqbal M., Alshememry A., Alkholief M., Alshamsan A. (2022). Development and Evaluation of Chitosan Nanoparticles for Ocular Delivery of Tedizolid Phosphate. Molecules.

[220] Silva B., Gonçalves L.M., São Braz B., Delgado E. (2023). Topical ocular delivery of nanoparticles with epoetin beta in Wistar Hannover rats. Sci. Rep..

[221] Omran S., Elnaggar Y.S.R., Abdallah O.Y. (2024). Controlled release, chitosan-tethered luteolin phytocubosomes; Formulation optimization to in-vivo antiglaucoma and anti-inflammatory ocular evaluation. Int. J. Biol. Macromol..

[222] Rahbar N., Darvish S., Farrahi F., Kouchak M. (2024). Chitosan/carbomer nanoparticles-laden in situ gel for improved ocular delivery of timolol: In vitro, in vivo, and ex vivo study. Drug Deliv. Transl. Res..

[223] Shajari G., Erfan-Niya H., Fathi M., Amiryaghoubi N. (2024). In situ forming hydrogels based on modified gellan gum/chitosan for ocular drug delivery of timolol maleate. Int. J. Biol. Macromol..

[224] Shahab M.S., Rizwanullah M., Alshehri S., Imam S.S. (2020). Optimization to development of chitosan decorated polycaprolactone nanoparticles for improved ocular delivery of dorzolamide: In vitro, ex vivo and toxicity assessments. Int. J. Biol. Macromol..

[225] Kailasam V., Kumara B.N., Prasad K.S., Nirmal J. (2024). Combination of self-assembling system and N,O-carboxymethyl chitosan improves ocular residence of anti-glaucoma drug. Eur. J. Pharm. Biopharm..

[226] Badran M.M., Alomrani A.H., Almomen A., Bin Jardan Y.A., Abou El Ela A.E.S. (2022). Novel Metoprolol-Loaded Chitosan-Coated Deformable Liposomes in Thermosensitive In Situ Gels for the Management of Glaucoma: A Repurposing Approach. Gels.

[227] Xiong X., Jiang H., Liao Y., Du Y., Zhang Y., Wang Z., Zheng M., Du Z. (2023). Liposome-trimethyl chitosan nanoparticles codeliver insulin and siVEGF to treat corneal alkali burns by inhibiting ferroptosis. Bioeng. Transl. Med..

[228] Sharma D.S., Wadhwa S., Gulati M., Kumar B., Chitranshi N., Gupta V.K., Alrouji M., Alhajlah S., AlOmeir O., Vishwas S. (2023). Chitosan modified 5-fluorouracil nanostructured lipid carriers for treatment of diabetic retinopathy in rats: A new dimension to an anticancer drug. Int. J. Biol. Macromol..

[229] Mohamed H.B., Shafie M.A.A., Mekkawy A.I. (2022). Chitosan Nanoparticles for Meloxicam Ocular Delivery: Development, In Vitro Characterization, and In Vivo Evaluation in a Rabbit Eye Model. Pharmaceutics.

[230] Xu X., Sun L., Zhou L., Cheng Y., Cao F. (2020). Functional chitosan oligosaccharide nanomicelles for topical ocular drug delivery of dexamethasone. Carbohydr. Polym..

[231] Arafa M.G., Girgis G.N.S., El-Dahan M.S. (2020). Chitosan-coated PLGA nanoparticles for enhanced ocular anti-inflammatory efficacy of atorvastatin calcium. Int. J. Nanomed..

[232] Alqurshi A., Hanafy A.F., Abdalla A.M., Guda T.K., Gabr K.E., Royall P.G. (2019). Ocular anti-inflammatory activity of prednisolone acetate loaded chitosan-deoxycholate self-assembled nanoparticles. Int. J. Nanomed..

[233] Fathalla Z., Al Fatease A., Abdelkader H. (2023). Formulation and In-Vitro/Ex-Vivo Characterization of Pregelled Hybrid Alginate-Chitosan Microparticles for Ocular Delivery of Ketorolac Tromethamine. Polymers.

[234] Adwan S., Al-Akayleh F., Qasmieh M., Obeidi T. (2024). Enhanced Ocular Drug Delivery of Dexamethasone Using a Chitosan-Coated Soluplus-Based Mixed Micellar System. Pharmaceutics.

[235] Xing Y., Zhu L., Zhang K., Li T., Huang S. (2021). Nanodelivery of triamcinolone acetonide with PLGA-chitosan nanoparticles for the treatment of ocular inflammation. Artif. Cells Nanomed. Biotechnol..

[236] Dandamudi M., McLoughlin P., Behl G., Rani S., Coffey L., Chauhan A., Kent D., Fitzhenry L. (2021). Chitosan-coated PLGA nanoparticles encapsulating triamcinolone acetonide as a potential candidate for sustained ocular drug delivery. Pharmaceutics.

[237] Yamaguchi M., Ueda K., Isowaki A., Ohtori A., Takeuchi H., Ohguro N., Tojo K. (2009). Mucoadhesive Properties of Chitosan-Coated Ophthalmic Lipid Emulsion Containing Indomethacin in Tear Fluid. J. Pharm. Soc. Jpn..

[238] Rahman S.N.R., Agarwal N., Goswami A., Sree A., Jala A., Venuganti A., Deka A., Borkar R.M., Singh V., Das D. (2023). Studies on spray dried topical ophthalmic emulsions containing cyclosporin A (0.05% w/w): Systematic optimization, in vitro preclinical toxicity and in vivo assessments. Drug Deliv. Transl. Res..

[239] Teba H.E., Khalil I.A., Gebreel R.M., Fahmy L.I., Sorogy H.M.E. (2024). Development of antifungal fibrous ocular insert using freeze-drying technique. Drug Deliv. Transl. Res..

[240] Franca J.R., Foureaux G., Fuscaldi L.L., Ribeiro T.G., Castilho R.O., Yoshida M.I., Cardoso V.N., Fernandes S.O.A., Cronemberger S., Nogueira J.C. (2019). Chitosan/hydroxyethyl cellulose inserts for sustained-release of dorzolamide for glaucoma treatment: In vitro and in vivo evaluation. Int. J. Pharm..

[241] Mirzaeei S., Taghe S., Asare-Addo K., Nokhodchi A. (2021). Polyvinyl Alcohol/Chitosan Single-Layered and Polyvinyl Alcohol/Chitosan/Eudragit RL100 Multi-Layered Electrospun Nanofibers as an Ocular Matrix for the Controlled Release of Ofloxacin: An In Vitro and In Vivo Evaluation. AAPS PharmSciTech.

[242] Franca J.R., Foureaux G., Fuscaldi L.L., Ribeiro T.G., Rodrigues L.B., Bravo R., Castilho R.O., Yoshida M.I., Cardoso V.N., Fernandes S.O. (2014). Bimatoprost-loaded ocular inserts as sustained release drug delivery systems for glaucoma treatment: In Vitro and In Vivo evaluation. PLoS ONE.

[243] Cesar A.L.A., Navarro L.C., Castilho R.O., Goulart G.A.C., Foureaux G., Ferreira A.J., Cronemberger S., Gomes Faraco A.A. (2021). New antiglaucomatous agent for the treatment of open angle glaucoma: Polymeric inserts for drug release and in vitro and in vivo study. J. Biomed. Mater. Res. A.

[244] Silva D., de Sousa H.C., Gil M.H., Santos L.F., Moutinho G.M., Salema-Oom M., Alvarez-Lorenzo C., Serro A.P., Saramago B. (2020). Diclofenac sustained release from sterilised soft contact lens materials using an optimised layer-by-layer coating. Int. J. Pharm..

[245] Anirudhan T.S., Nair A.S., Parvathy J. (2016). Extended wear therapeutic contact lens fabricated from timolol imprinted carboxymethyl chitosan-g-hydroxy ethyl methacrylate-g-poly acrylamide as a onetime medication for glaucoma. Eur. J. Pharm. Biopharm..

[246] Behl G., Iqbal J., O’Reilly N.J., McLoughlin P., Fitzhenry L. (2016). Synthesis and Characterization of Poly(2-hydroxyethylmethacrylate) Contact Lenses Containing Chitosan Nanoparticles as an Ocular Delivery System for Dexamethasone Sodium Phosphate. Pharm. Res..

[247] Jiao Z., Huo Q., Lin X., Chu X., Deng Z., Guo H., Peng Y., Lu S., Zhou X., Wang X. (2022). Drug-free contact lens based on quaternized chitosan and tannic acid for bacterial keratitis therapy and corneal repair. Carbohydr. Polym..

[248] Mehta P., Al-Kinani A.A., Arshad M.S., Singh N., van der Merwe S.M., Chang M.W., Alany R.G., Ahmad Z. (2019). Engineering and Development of Chitosan-Based Nanocoatings for Ocular Contact Lenses. J. Pharm. Sci..

[249] Hoyo J., Ivanova K., Guaus E., Tzanov T. (2019). Multifunctional ZnO NPs-chitosan-gallic acid hybrid nanocoating to overcome contact lenses associated conditions and discomfort. J. Colloid Interface Sci..

[250] Ibrahim S.S. (2019). The Role of Surface Active Agents in Ophthalmic Drug Delivery: A Comprehensive Review. J. Pharm. Sci..

[251] Berthod A., Tomer S., Dorsey J.G. (2001). Polyoxyethylene alkyl ether nonionic surfactants: Physicochemical properties and use for cholesterol determination in food. Talanta.

[252] Tomasino C. (2005). Effect of wet processing and chemical finishing on fabric hand. Effect of Mechanical and Physical Properties on Fabric Hand.

[253] Abdelbary G., El-Gendy N. (2008). Niosome-encapsulated gentamicin for ophthalmic controlled delivery. AAPS PharmSciTech.

[254] Kapoor Y., Howell B.A., Chauhan A. (2009). Liposome assay for evaluating ocular toxicity of surfactants. Investig. Ophthalmol. Vis. Sci..

[255] Matsuda S., Hisama M., Shibayama H., Norihiko I., Iwaki M. (2009). In Vitro Eye Irritancy Test of Lauryl Derivatives and Polyoxyethylene Alkyl Derivatives with the Reconstructed Rabbit Corneal Epithelium Model. J. Oleo Sci..

[256] Chiou G.C.Y., Shen Z.F., Zheng Y.Q., Chen Y.J. (1992). Enhancement of Systemic Delivery of Peptide Drugs via Ocular Route with Surfactants. Drug Dev. Res..

[257] Chiou G.C.Y., Li B.H.P. (1993). Chronic Systemic Delivery of Insulin Through the Ocular Route. J. Ocul. Pharmacol..

[258] Rohde B.H., Chiou G.C.Y. (1991). Effect of Permeation Enhancers on Beta-Endorphin Systemic Uptake After Topical Application to the Eye. Ophthalmic Res..

[259] Lee Y.C., Simamora P., Yalkowsky S.H. (1997). Effect of Brij-78 on Systemic Delivery of Insulin from an Ocular Device. Int. J. Pharm..

[260] Srinivasan R., Jain S.K. (1998). Insulin delivery through the ocular route. Drug Deliv..

[261] Morgan R.V. (1995). Delivery of Systemic Regular Insulin Via the Ocular Route in Cats. J. Ocul. Pharmacol. Ther..

[262] Furrer P., Mayer J.M., Plazonnet B., Gurny R. (2002). Ocular Tolerance of Absorption Enhancers in Ophthalmic Preparations. Int. J. Pharm..

[263] Chiou G.C.Y., Chlng A., Chuang Y. (1989). Improvement of Systemic Absorption of Insulin Through Eyes with Absorption Enhancers. J. Pharm. Sci..

[264] Chiou G.C.Y., Shen Z.F., Zheng Y.Q. (1991). Systemic Absorption of Oxytocin and Vasopressin Through Eyes in Rabbits. J. Ocul. Pharmacol..

[265] Pillion D.J., Atchison J.A., Stott J., McCracken D., Gargiulo C., Meezan E. (1994). Efficacy of Insulin Eyedrops. J. Ocul. Pharmacol..

[266] Morgan R.V., Huntzicker M.A. (1996). Delivery of Systemic Regular Insulin via the Ocular Route in Dogs. J. Ocul. Pharmacol..

[267] Pillion D.J., McCracken D.L., Yang M., Atchison J.A. (1992). Glucagon Administration to the Rat via Eye Drops. J. Ocul. Pharmacol..

[268] Fruijtier-Pölloth C. (2005). Safety assessment on polyethylene glycols (PEGs) and their derivatives as used in cosmetic products. Toxicology.

[269] Xiong G.M., Ang H., Lin J., Lui Y.S., Phua J.L., Chan J.N., Venkatraman S., Foin N., Huang Y. (2016). Materials technology in drug eluting balloons: Current and future perspectives. J. Control. Release.

[270] Durak S., Rad M.E., Yetisgin A.A., Sutova H.E., Kutlu O., Cetinel S., Zarrabi A. (2020). Niosomal drug delivery systems for ocular disease-recent advances and future prospects. Nanomaterials.

[271] Zimmer A.K., Maincent P., Thouvenot P., Kreuter J. (1994). Hydrocortisone delivery to healthy and inflamed eyes using a micellar polysorbate 80 solution or albumin nanoparticles. Int. J. Pharm..

[272] Taniguchi K., Itakura K., Morisaki K., Hayashi S. (1988). Effects of Tween 80 and Liposomes on the Corneal Permeability of Anti-Inflammatory Steroids. J. Pharmacobiodyn..

[273] Barbalho G.N., Brugger S., Raab C., Lechner J.S., Gratieri T., Keck C.M., Rupenthal I.D., Agarwal P. (2024). Development of Transferosomes for Topical Ocular Drug Delivery of Curcumin. Eur. J. Pharm. Biopharm..

[274] Kakkar S., Kaur I.P. (2011). Spanlastics—A novel nanovesicular carrier system for ocular delivery. Int. J. Pharm..

[275] Ibrahim S.S., Abd-allah H. (2022). Spanlastic nanovesicles for enhanced ocular delivery of vanillic acid: Design, in vitro characterization, and in vivo anti-inflammatory evaluation. Int. J. Pharm..

[276] ElMeshad A.N., Mohsen A.M. (2016). Enhanced corneal permeation and antimycotic activity of itraconazole against Candida albicans via a novel nanosystem vesicle. Drug Deliv..

[277] Abdelbari M.A., El-Mancy S.S., Elshafeey A.H., Abdelbary A.A. (2021). Implementing spanlastics for improving the ocular delivery of clotrimazole: In vitro characterization, ex vivo permeability, microbiological assessment and in vivo safety study. Int. J. Nanomed..

[278] Üstündağ-Okur N., Gökçe E.H., Bozbiyik D.I., Eğrilmez S., Özer Ö., Ertan G. (2014). Preparation and in vitro-in vivo evaluation of ofloxacin loaded ophthalmic nano structured lipid carriers modified with chitosan oligosaccharide lactate for the treatment of bacterial keratitis. Eur. J. Pharm. Sci..

[279] Naguib S.S., Hathout R.M., Mansour S. (2017). Optimizing novel penetration enhancing hybridized vesicles for augmenting the in-vivo effect of an anti-glaucoma drug. Drug Deliv..

[280] Hippalgaonkar K., Adelli G.R., Hippalgaonkar K., Repka M.A., Majumdar S. (2013). Indomethacin-loaded solid lipid nanoparticles for ocular delivery: Development, characterization, and in vitro evaluation. J. Ocul. Pharmacol. Ther..

[281] Tatke A., Dudhipala N., Janga K.Y., Balguri S.P., Avula B., Jablonski M.M., Majumdar S. (2019). In situ gel of triamcinolone acetonide-loaded solid lipid nanoparticles for improved topical ocular delivery: Tear kinetics and ocular disposition studies. Nanomaterials.

[282] Alvarez-Trabado J., López-García A., Martín-Pastor M., Diebold Y., Sanchez A. (2018). Sorbitan ester nanoparticles (SENS) as a novel topical ocular drug delivery system: Design, optimization, and in vitro/ex vivo evaluation. Int. J. Pharm..

[283] Patel N., Nakrani H., Raval M., Sheth N. (2016). Development of loteprednol etabonate-loaded cationic nanoemulsified in-situ ophthalmic gel for sustained delivery and enhanced ocular bioavailability. Drug Deliv..

[284] Tayel S.A., El-Nabarawi M.A., Tadros M.I., Abd-Elsalam W.H. (2013). Promising ion-sensitive in situ ocular nanoemulsion gels of terbinafine hydrochloride: Design, in vitro characterization and in vivo estimation of the ocular irritation and drug pharmacokinetics in the aqueous humor of rabbits. Int. J. Pharm..

[285] Moghimipour E., Farsimadan N., Salimi A. (2022). Ocular Delivery of Quercetin Using Microemulsion System: Design, Characterization, and Ex-Vivo Transcorneal Permeation. Iran. J. Pharm. Res..

[286] Bharti S.K., Kesavan K. (2017). Phase-Transition W/O Microemulsions for Ocular Delivery: Evaluation of Antibacterial Activity in the Treatment of Bacterial Keratitis. Ocul. Immunol. Inflamm..

[287] Soliman O.A.E.A., Mohamed E.A., Khatera N.A.A. (2019). Enhanced ocular bioavailability of fluconazole from niosomal gels and microemulsions: Formulation, optimization, and in vitro-in vivo evaluation. Pharm. Dev. Technol..

[288] Mohammadi M., Elahimehr Z., Mahboobian M.M. (2021). Acyclovir-Loaded Nanoemulsions: Preparation, Characterization and Irritancy Studies for Ophthalmic Delivery. Curr. Eye Res..

[289] Agha O.A., Girgis G.N.S., El-Sokkary M.M.A., Soliman O.A.E.A. (2023). Spanlastic-laden in situ gel as a promising approach for ocular delivery of Levofloxacin: In-vitro characterization, microbiological assessment, corneal permeability and in-vivo study. Int. J. Pharm. X.

[290] Maher S., Geoghegan C., Brayden D.J. (2023). Safety of surfactant excipients in oral drug formulations. Adv. Drug Deliv. Rev..

[291] Yasser M., El Naggar E.E., Elfar N., Teaima M.H., El-Nabarawi M.A., Elhabal S.F. (2024). Formulation, optimization and evaluation of ocular gel containing nebivolol HCl-loaded ultradeformable spanlastics nanovesicles: In vitro and in vivo studies. Int. J. Pharm. X.

[292] Abdelmonem R., Elhabal S.F., Abdelmalak N.S., El-Nabarawi M.A., Teaima M.H. (2021). Formulation and characterization of acetazolamide/carvedilol niosomal gel for glaucoma treatment: In vitro, and in vivo study. Pharmaceutics.

[293] Jain N., Verma A., Jain N. (2020). Formulation and investigation of pilocarpine hydrochloride niosomal gels for the treatment of glaucoma: Intraocular pressure measurement in white albino rabbits. Drug Deliv..

[294] Aldawsari M.F., Moglad E.H., Alotaibi H.F., Alkahtani H.M., Khafagy E.S. (2023). Ophthalmic Bimatoprost-Loaded Niosomal In Situ Gel: Preparation, Optimization, and In Vivo Pharmacodynamics Study. Polymers.

[295] Sayed S., Abdelmoteleb M., Amin M.M., Khowessah O.M. (2020). Effect of Formulation Variables and Gamma Sterilization on Transcorneal Permeation and Stability of Proniosomal Gels as Ocular Platforms for Antiglaucomal Drug. AAPS PharmSciTech.

[296] Yousry C., Zikry P.M., Salem H.M., Basalious E.B., El-Gazayerly O.N. (2020). Integrated nanovesicular/self-nanoemulsifying system (INV/SNES) for enhanced dual ocular drug delivery: Statistical optimization, in vitro and in vivo evaluation. Drug Deliv. Transl. Res..

[297] Shukr M.H. (2016). Novel in situ gelling ocular inserts for voriconazole-loaded niosomes: Design, in vitro characterisation and in vivo evaluation of the ocular irritation and drug pharmacokinetics. J. Microencapsul..

[298] Fouda N.H., Abdelrehim R.T., Hegazy D.A., Habib B.A. (2018). Sustained ocular delivery of dorzolamide-HCL via proniosomal gel formulation: In-vitro characterization, statistical optimization, and in-vivo pharmacodynamic evaluation in rabbits. Drug Deliv..

[299] Jin Q., Li H., Jin Z., Huang L., Wang F., Zhou Y., Liu Y., Jiang C., Oswald J., Wu J. (2018). TPGS modified nanoliposomes as an effective ocular delivery system to treat glaucoma. Int. J. Pharm..

[300] Ostacolo C., Caruso C., Tronino D., Troisi S., Laneri S., Pacente L., Del Prete A., Sacchi A. (2013). Enhancement of corneal permeation of riboflavin-5′-phosphate through vitamin E TPGS: A promising approach in corneal trans-epithelial cross linking treatment. Int. J. Pharm..

[301] Kumbhar P.S., Nadaf S., Manjappa A.S., Jha N.K., Shinde S.S., Chopade S.S., Shete A.S., Disouza J.I., Sambamoorthy U., Kumar S.A. (2022). D-ɑ-tocopheryl polyethylene glycol succinate: A review of multifarious applications in nanomedicines. OpenNano.

[302] Vadlapudi A.D., Cholkar K., Vadlapatla R.K., Mitra A.K. (2014). Aqueous nanomicellar formulation for topical delivery of biotinylated lipid prodrug of acyclovir: Formulation development and ocular biocompatibility. J. Ocul. Pharmacol. Ther..

[303] Caruso C., Porta A., Tosco A., Eletto D., Pacente L., Bartollino S., Costagliola C. (2020). A novel vitamin E TPGS-based formulation enhances chlorhexidine bioavailability in corneal layers. Pharmaceutics.

[304] Signorini S., Pescina S., Ricci C., del Favero E., Vivero-Lopez M., Alvarez-Lorenzo C., Santi P., Padula C., Nicoli S. (2024). Innovative formulations for the ocular delivery of coenzyme Q10. Drug Deliv. Transl. Res..

[305] Lam C.H.I., Zuo B., Chan H.H.L., Leung T.W., Abokyi S., Catral K.P.C., Tse D.Y.Y. (2024). Coenzyme Q10 eyedrops conjugated with vitamin E TPGS alleviate neurodegeneration and mitochondrial dysfunction in the diabetic mouse retina. Front. Cell. Neurosci..

[306] Guo P., Li N., Fan L., Lu J., Liu B., Zhang B., Wu Y., Liu Z., Li J., Pi J. (2019). Study of penetration mechanism of labrasol on rabbit cornea by Ussing chamber, RT-PCR assay, Western blot and immunohistochemistry. Asian J. Pharm. Sci..

[307] Liu Z., Zhang X., Li J., Liu R., Shu L., Jin J. (2009). Effects of Labrasol on the corneal drug delivery of baicalin. Drug Deliv..

[308] Huang L., Bai J., Yang H., Liu J., Cui H. (2015). Combined use of borneol or menthol with labrasol promotes penetration of baicalin through rabbit cornea in vitro. Eur. J. Pharm. Sci..

[309] Ibrahim M.M., Maria D.N., Wang X.D., Simpson R.N., Hollingsworth T.J., Jablonski M.M. (2020). Enhanced corneal penetration of a poorly permeable drug using bioadhesive multiple microemulsion technology. Pharmaceutics.

[310] Liu R., Liu Z., Shu L., Zhang C., Zhang B. (2010). Effect of three penetration enhancers on corneal permeability of mangiferin in vitro. Zhongguo Zhongyao Zazhi.

[311] Montenegro L., Bucolo C., Puglisi G., Montenegro L. (2003). Enhancer effects on in vitro corneal permeation of timolol and acyclovir. Int. J. Pharm..

[312] Li X., Pan W., Ju C., Liu Z., Pan H., Zhang H., Nie S. (2009). Evaluation of Pharmasolve^®^ corneal permeability enhancement and its irritation on rabbit eyes. Drug Deliv..

[313] Gutiérrez-Méndez N., Chavez-Garay D.R., Leal-Ramos M.Y. (2022). Lecithins: A comprehensive review of their properties and their use in formulating microemulsions. J. Food Biochem..

[314] List G.R. (2015). Soybean Lecithin: Food, Industrial Uses, and Other Applications. Polar Lipids: Biology, Chemistry, and Technology.

[315] Caparosa M.H., Hartel R.W. (2020). Characterizing Lecithin Interactions in Chocolate Using Interfacial Properties and Rheology. J. Am. Oil Chem. Soc..

[316] Kent C. (2005). Regulatory enzymes of phosphatidylcholine biosynthesis: A personal perspective. Biochim. Biophys. Acta.

[317] Waite K.A., Vance D.E. (2004). Dimethylethanolamine does not prevent liver failure in phosphatidylethanolamine N-methyltransferase-deficient mice fed a choline-deficient diet. Biochim. Biophys. Acta.

[318] Exton J.H. (1994). Phosphatidylcholine breakdown and signal transduction. Biochim. Biophys. Acta.

[319] Spernath A., Aserin A., Ziserman L., Danino D., Garti N. (2007). Phosphatidylcholine embedded microemulsions: Physical properties and improved Caco-2 cell permeability. J. Control. Release.

[320] Chetoni P., Monti D., Tampucci S., Matteoli B., Ceccherini-Nelli L., Subissi A., Burgalassi S. (2015). Liposomes as a potential ocular delivery system of distamycin A. Int. J. Pharm..

[321] Tan G., Yu S., Pan H., Li J., Liu D., Yuan K., Yang X., Pan W. (2017). Bioadhesive chitosan-loaded liposomes: A more efficient and higher permeable ocular delivery platform for timolol maleate. Int. J. Biol. Macromol..

[322] Londhe V.Y., Sharma S. (2022). Formulation, characterization, optimization and in-vivo evaluation of methazolamide liposomal in-situ gel for treating glaucoma. J. Drug Deliv. Sci. Technol..

[323] Peng X., Zhang T., Wu Y., Wang X., Liu R., Jin X. (2023). mPEG-CS-modified flexible liposomes-reinforced thermosensitive sol-gel reversible hydrogels for ocular delivery of multiple drugs with enhanced synergism. Colloids Surf. B.

[324] Goldstein M.H., Silva F.Q., Blender N., Tran T., Vantipalli S. (2022). Ocular benzalkonium chloride exposure: Problems and solutions. Eye.

[325] Bacchetti F., Schito A.M., Milanese M., Castellaro S., Alfei S. (2024). Anti Gram-Positive Bacteria Activity of Synthetic Quaternary Ammonium Lipid and Its Precursor Phosphonium Salt. Int. J. Mol. Sci..

[326] Karamov E.V., Larichev V.F., Kornilaeva G.V., Fedyakina I.T., Turgiev A.S., Shibaev A.V., Molchanov V.S., Philippova O.E., Khokhlov A.R. (2022). Cationic Surfactants as Disinfectants Against SARS-CoV-2. Int. J. Mol. Sci..

[327] Barros A.C., Melo L.F., Pereira A. (2022). A Multi-Purpose Approach to the Mechanisms of Action of Two Biocides (Benzalkonium Chloride and Dibromonitrilopropionamide): Discussion of Pseudomonas Fluorescens’ Viability and Death. Front. Microbiol..

[328] Schito A.M., Piatti G., Caviglia D., Zuccari G., Alfei S. (2021). Broad-spectrum bactericidal activity of a synthetic random copolymer based on 2-methoxy-6-(4-vinylbenzyloxy)benzylammonium hydrochloride. Int. J. Mol. Sci..

[329] McCarlie S.J., du Preez L.L., Hernandez J.C., Boucher C.E., Bragg R.R. (2024). Transcriptomic signature of bacteria exposed to benzalkonium chloride. Res. Microbiol..

[330] Baudouin C., Labbé A., Liang H., Pauly A., Brignole-Baudouin F. (2010). Preservatives in eyedrops: The good, the bad and the ugly. Prog. Retin. Eye Res..

[331] Green K., Chapman J. (1986). Benzalkonium chloride kinetics in young and adult albino and pigmented rabbit eyes. Cutan. Ocul. Toxicol..

[332] Thacker M., Sahoo A., Reddy A.A., Bokara K.K., Singh S., Basu S., Singh V. (2023). Benzalkonium chloride-induced dry eye disease animal models: Current understanding and potential for translational research. Indian J. Ophthalmol..

[333] Georgiev G.A., Yokoi N., Koev K., Kutsarova E., Ivanova S., Kyumurkov A., Jordanova A., Krastev R., Lalchev Z. (2011). Surface chemistry study of the interactions of benzalkonium chloride with films of meibum, corneal cells lipids, and whole tears. Investig. Ophthalmol. Vis. Sci..

[334] Green K., Tonjum A. (1971). Influence of various agents on corneal permeability. Am. J. Ophthalmol..

[335] Majumdar S., Hippalgaonkar K., Repka M.A. (2008). Effect of chitosan, benzalkonium chloride and ethylenediaminetetraacetic acid on permeation of acyclovir across isolated rabbit cornea. Int. J. Pharm..

[336] Roscoe W.R., Durstein N.L. (1984). Preservative Alteration of Corneal Permeability in Humans and Rabbits. J. Toxicol. Cutan. Ocul. Toxicol..

[337] Saettone M.F., Chetoni P., Cerbai R., Mazzanti G., Braghiroli L. (1996). Evaluation of ocular permeation enhancers: In vitro effects on corneal transport of four β-blockers, and in vitro/in vivo toxic activity. Int. J. Pharm..

[338] Johannsdottir S., Jansook P., Stefansson E., Kristinsdottir I.M., Asgrimsdottir G.M., Loftsson T. (2018). Topical drug delivery to the posterior segment of the eye: The effect of benzalkonium chloride on topical dexamethasone penetration into the eye in vivo. J. Drug Deliv. Sci. Technol..

[339] Rouland J.F., Traverso C.E., Stalmans I., El Fekih L., Delval L., Renault D., Baudouin C. (2013). Efficacy and safety of preservative-free latanoprost eyedrops, compared with BAK-preserved latanoprost in patients with ocular hypertension or glaucoma. Br. J. Ophthalmol..

[340] Aptel F., Choudhry R., Stalmans I. (2016). Preservative-free versus preserved latanoprost eye drops in patients with open-angle glaucoma or ocular hypertension. Curr. Med. Res. Opin..

[341] Goldberg I., Gil Pina R., Lanzagorta-Aresti A., Schiffman R.M., Liu C., Bejanian M. (2014). Bimatoprost 0.03%/timolol 0.5% preservative-free ophthalmic solution versus bimatoprost 0.03%/timolol 0.5% ophthalmic solution (Ganfort) for glaucoma or ocular hypertension: A 12-week randomised controlled trial. Br. J. Ophthalmol..

[342] Peace J.H., Ahlberg P., Wagner M., Lim J.M., Wirta D., Branch J.D. (2015). Polyquaternium-1-Preserved Travoprost 0.003% or Benzalkonium Chloride-Preserved Travoprost 0.004% for Glaucoma and Ocular Hypertension. Am. J. Ophthalmol..

[343] Cordeiro M.F., Goldberg I., Schiffman R., Bernstein P., Bejanian M. (2015). Efficacy of a preservative-free formulation of fixed-combination bimatoprost and timolol (Ganfort PF) in treatment-naïve patients vs previously treated patients. Clin. Ophthalmol..

[344] Tokuda N., Kitaoka Y., Matsuzawa A., Tsukamoto A., Sase K., Sakae S., Takagi H. (2017). Changes in Ocular Surface Characteristics after Switching from Benzalkonium Chloride-Preserved Latanoprost to Preservative-Free Tafluprost or Benzalkonium Chloride-Preserved Tafluprost. J. Ophthalmol..

[345] Amiri D., Sessa M., Andersen M., Kolko M. (2024). Persistence and adherence with Latanoprost: A Danish register-based cohort study in older patients with glaucoma. Acta Ophthalmol..

[346] Aptel F., Pfeiffer N., Schmickler S., Clarke J., Lavín-Dapena C., Moreno-Montañés J., Zarnowski T., Csutak A., Jugaste T., Volksone L. (2019). Noninferiority of Preservative-free Versus BAK-preserved Latanoprost-timolol Fixed Combination Eye Drops in Patients with Open-angle Glaucoma or Ocular Hypertension. J. Glaucoma.

[347] Kitazawa Y., Smith P., Sasaki N., Kotake S., Bae K., Iwamoto Y. (2011). Travoprost 0.004%/timolol 0.5%-fixed combination with and without benzalkonium chloride: A prospective, randomized, doubled-masked comparison of safety and efficacy. Eye.

[348] Lewis R.A., Katz G.J., Weiss M.J., Landry T.A., Dickerson J.E., James J.E., Hua S.Y., Sullivan E.K., Montgomery D.B., Wells D.T. (2007). Travoprost 0.004% With and Without Benzalkonium Chloride: A Comparison of Safety and Efficacy. J. Glaucoma.

[349] Tonjum A.M. (1975). Effects of Benzalkonium Chloride Upon The Corneal Epithelium Studied with Scanning Electron Microscopy. Acta Ophthalmol..

[350] Debbasch C., Brignole F., Pisella P.-J., Warnet J.-M., Rat P., Baudouin C. (2001). Quaternary Ammoniums and Other Preservatives’ Contribution in Oxidative Stress and Apoptosis on Chang Conjunctival Cells. Investig. Ophthalmol. Vis. Sci..

[351] Rogov A.G., Goleva T.N., Sukhanova E.I., Epremyan K.K., Trendeleva T.A., Ovchenkova A.P., Aliverdieva D.A., Zvyagilskaya R.A. (2020). Mitochondrial Dysfunctions May Be One of the Major Causative Factors Underlying Detrimental Effects of Benzalkonium Chloride. Oxid. Med. Cell. Longev..

[352] Datta S., Baudouin C., Brignole-Baudouin F., Denoyer A., Cortopassi G.A. (2017). The eye drop preservative benzalkonium chloride potently induces mitochondrial dysfunction and preferentially affects LHON mutant cells. Investig. Ophthalmol. Vis. Sci..

[353] Ammar D.A., Kahook M.Y. (2011). Effects of glaucoma medications and preservatives on cultured human trabecular meshwork and non-pigmented ciliary epithelial cell lines. Br. J. Ophthalmol..

[354] Ammar D.A., Noecker R.J., Kahook M.Y. (2010). Effects of benzalkonium chloride-preserved, polyquad-preserved, and sofZia-preserved topical glaucoma medications on human ocular epithelial cells. Adv. Ther..

[355] Ayaki M., Iwasawa A. (2010). Cytotoxicity of prostaglandin analog eye drops preserved with benzalkonium chloride in multiple corneoconjunctival cell lines. Clin. Ophthalmol..

[356] Ayaki M., Iwasawa A., Inoue Y. (2010). Toxicity of antiglaucoma drugs with and without benzalkonium chloride to cultured human corneal endothelial cells. Clin. Ophthalmol..

[357] Guzman-Aranguez A., Calvo P., Ropero I., Pintor J. (2014). In vitro effects of preserved and unpreserved anti-allergic drugs on human corneal epithelial cells. J. Ocul. Pharmacol. Ther..

[358] Kim J.H., Kim E.J., Kim Y.H., Kim Y.I., Lee S.H., Jung J.C., Lee K.W., Park Y.J. (2015). In Vivo Effects of Preservative-free and Preserved Prostaglandin Analogs: Mouse Ocular Surface Study. Korean J. Ophthalmol..

[359] Kim Y.-H., Jung J.-C., Jung S.-Y., Yu S., Lee K.W., Park Y.J. (2015). Comparison of the Efficacy of Fluorometholone With and Without Benzalkonium Chloride in Ocular Surface Disease. J. Ocul. Pharmacol. Ther..

[360] Pauly A., Brasnu E., Riancho L., Brignole-Baudouin F., Baudouin C. (2011). Multiple endpoint analysis of BAC-preserved and unpreserved antiallergic eye drops on a 3D-reconstituted corneal epithelial model. Mol. Vis..

[361] Izzotti A., La Maestra S., Micale R.T., Longobardi M.G., Saccà S.C. (2015). Genomic and post-genomic effects of anti-glaucoma drugs preservatives in trabecular meshwork. Mutat. Res..

[362] Kahook M.Y., Noecker R. (2008). Quantitative analysis of conjunctival goblet cells after chronic application of topical drops. Adv. Ther..

[363] Liang H., Brignole-Baudouin F., Riancho L., Baudouin C. (2012). Reduced in vivo ocular surface toxicity with polyquad-preserved travoprost versus benzalkonium-preserved travoprost or latanoprost ophthalmic solutions. Ophthalmic Res..

[364] Hedengran A., Freiberg J., May Hansen P., Boix-Lemonche G., Utheim T.P., Dartt D.A., Petrovski G., Heegaard S., Kolko M. (2024). Comparing the effect of benzalkonium chloride-preserved, polyquad-preserved, and preservative-free prostaglandin analogue eye drops on cultured human conjunctival goblet cells. J. Optom..

[365] Nagai N., Murao T., Okamoto N., Ito Y. (2010). Comparison of Corneal Wound Healing Rates after Instillation of Commercially Available Latanoprost and Travoprost in Rat Debrided Corneal Epithelium. J. Oleo Sci..

[366] Jaenen N., Baudouin C., Pouliquen P., Manni G., Figueiredo A., Zeyen T. (2007). Ocular symptoms and signs with preserved and preservative-free glaucoma medications. Eur. J. Ophthalmol..

[367] Uusitalo H., Egorov E., Kaarniranta K., Astakhov Y., Ropo A. (2016). Benefits of switching from latanoprost to preservative-free tafluprost eye drops: A meta-analysis of two phase IIIb clinical trials. Clin. Ophthalmol..

[368] Horsley M.B., Kahook M.Y. (2009). Effects of prostaglandin analog therapy on the ocular surface of glaucoma patients. Clin. Ophthalmol..

[369] Hommer A., Kimmich F. (2011). Switching patients from preserved prostaglandin-analog monotherapy to preservative-free tafluprost. Clin. Ophthalmol..

[370] Lopes N.L.V., Gracitelli C.P.B., Chalita M.R., Faria N.V.L. (2019). Ocular Surface Evaluation After the Substitution of Benzalkonium Chloride Preserved Prostaglandin Eye Drops by a Preservative-free Prostaglandin Analogue. Discov. Innov. Ophthalmol. J..

[371] Rossi G.C.M., Scudeller L., Rolle T., Pasinetti G.M., Bianchi P.E. (2015). From benzalkonium chloride-preserved Latanoprost to Polyquad-preserved Travoprost: A 6-month study on ocular surface safety and tolerability. Expert Opin. Drug Saf..

[372] Aihara M., Oshima H., Araie M. (2013). Effects of SofZia-preserved travoprost and benzalkonium chloride-preserved latanoprost on the ocular surface—A multicentre randomized single-masked study. Acta Ophthalmol..

[373] Tomić M., Kaštelan S., Metež Soldo K., Salopek-Rabatić J. (2013). Influence of BAK-preserved prostaglandin analog treatment on the ocular surface health in patients with newly diagnosed primary open-angle glaucoma. Biomed Res. Int..

[374] Economou M.A., Laukeland H.K., Grabska-Liberek I., Rouland J.F. (2018). Better tolerance of preservative-free latanoprost compared to preserved glaucoma eye drops: The 12-month real-life FREE study. Clin. Ophthalmol..

[375] Lazreg S., Merad Z., Nouri M.T., Garout R., Derdour A., Ghroud N., Kherroubi R., Meziane M., Belkacem S., Ouhadj O. (2018). Efficacy and safety of preservative-free timolol 0.1% gel in open-angle glaucoma and ocular hypertension in treatment-naïve patients and patients intolerant to other hypotensive medications. J. Fr. Ophtalmol..

[376] Aihara M., Ikeda Y., Mizoue S., Arakaki Y., Kita N., Kobayashi S. (2016). Effect of switching to travoprost preserved with SofZia in glaucoma patients with chronic superficial punctate keratitis while receiving BAK-preserved latanoprost. J. Glaucoma.

[377] Noecker R. (2001). Effects of Common Ophthalmic Preservatives on Ocular Health. Adv. Ther..

[378] Chandran S., Roy A., Saha R.N. (2008). Effect of pH and Formulation Variables on In Vitro Transcorneal Permeability of Flurbiprofen: A Technical Note. AAPS PharmSciTech.

[379] Camber O., Edman P. (1987). Influence of some preservatives on the corneal permeability of pilocarpine and dexamethasone, in vitro. Int. J. Pharm..

[380] Gasset A.R., Ishii Y., Kaufman H.E., Miller T. (1974). Cytotoxicity of ophthalmic preservatives. Am. J. Ophthalmol..

[381] Mao X., Aue D.L., Buchalla W., Hiller K.A., Maisch T., Hellwig E., Al-Ahmad A., Cieplik F. (2020). Cetylpyridinium chloride: Mechanism of action, antimicrobial efficacy in biofilms, and potential risks of resistance. Antimicrob. Agents Chemother..

[382] Riveira-Muñoz E., Garcia-Vidal E., Bañó-Polo M., León R., Blanc V., Clotet B., Ballana E. (2023). Cetylpyridinium Chloride-Containing Mouthwashes Show Virucidal Activity against Herpes Simplex Virus Type 1. Viruses.

[383] D’Amico F., Moro M., Saracino M., Marmiere M., Cilona M.B., Lloyd-Jones G., Zangrillo A. (2023). Efficacy of Cetylpyridinium Chloride mouthwash against SARS-CoV-2: A systematic review of randomized controlled trials. Mol. Oral Microbiol..

[384] Godbey R.E.W., Green K., Hull D.S. (1973). Influence of Cetylpyridinium Chloride on Corneal Permeability to Penicillin. J. Pharm. Sci..

[385] Green K., Bowman K.A., Elijah R.D., Mermelstein R., Kilpper R.W. (1985). Dose-effect response of the rabbit Eye to cetylpyridinium chloride. Cutan. Ocul. Toxicol..

[386] Li X., Muller R.H., Keck C.M., Bou-Chacra N.A. (2016). Mucoadhesive dexamethasone acetate-polymyxin B sulfate cationic ocular nanoemulsion—Novel combinatorial formulation concept. Pharmazie.

[387] Romero G.B., Keck C.M., Müller R.H., Bou-Chacra N.A. (2016). Development of cationic nanocrystals for ocular delivery. Eur. J. Pharm. Biopharm..

[388] Karpinski T.M., Szkaradkiewicz A.K. (2015). Chlorhexidine—Pharmaco-biological activity and application. Eur. Rev. Med. Pharmacol. Sci..

[389] Ashton P., Diepold R., Platzer A., Lee V.H.L. (1990). The Effect of Chlorhexidine Acetate on the Corneal Penetration of Sorbitol from an Arnolol Formulation in the Albino Rabbit. J. Ocul. Pharmacol..

[390] Poppolo Deus F., Ouanounou A. (2022). Chlorhexidine in Dentistry: Pharmacology, Uses, and Adverse Effects. Int. Dent. J..

[391] Steinsapir K.D., Woodward J.A. (2017). Chlorhexidine Keratitis: Safety of Chlorhexidine as a Facial Antiseptic. Dermatol. Surg..

[392] Palka L., Nowakowska-Toporowska A., Dalewski B. (2022). Is Chlorhexidine in Dentistry an Ally or a Foe? A Narrative Review. Healthcare.

[393] Epstein N.E. (2021). Review: Perspective on ocular toxicity of presurgical skin preparations utilizing Chlorhexidine Gluconate/Hibiclens/Chloraprep. Surg. Neurol. Int..

[394] Bever G.J., Brodie F.L., Hwang D.G. (2016). Corneal Injury from Presurgical Chlorhexidine Skin Preparation. World Neurosurg..

[395] Romano V., Ferrara M., Gatti F., Airaldi M., Borroni D., Aragona E., Rocha-de-Lossada C., Gabrielli F., Papa F.T., Romano M.R. (2024). Topical Antiseptics in Minimizing Ocular Surface Bacterial Load Before Ophthalmic Surgery: A Randomized Controlled Trial. Am. J. Ophthalmol..

[396] de Buy Wenniger L.M., Pusl T., Beuers U. (2013). Bile Salts. Encyclopedia of Biological Chemistry.

[397] Tazuma S., Takikawa H. (2017). Bile Acids in Gastroenterology: Basic and Clinical.

[398] Boatright J.H., Nickerson J.M., Moring A.G., Pardue M.T. (2009). Bile acids in treatment of ocular disease. J. Ocul. Biol. Dis. Inform..

[399] Ridlon J.M., Harris S.C., Bhowmik S., Kang D.J., Hylemon P.B. (2016). Consequences of bile salt biotransformations by intestinal bacteria. Gut Microbes.

[400] Maldonado-Valderrama J., Wilde P., MacIerzanka A., MacKie A. (2011). The role of bile salts in digestion. Adv. Colloid Interface Sci..

[401] Monte M.J., Marin J.J.G., Antelo A., Vazquez-Tato J. (2009). Bile acids: Chemistry, physiology, and pathophysiology. World J. Gastroenterol..

[402] Neves M.C., Filipe H.A.L., Reis R.L., Ramalho J.P.P., Coreta-Gomes F., Moreno M.J., Loura L.M.S. (2019). Interaction of bile salts with lipid bilayers: An atomistic molecular dynamics study. Front. Physiol..

[403] Malik N.A. (2016). Solubilization and Interaction Studies of Bile Salts with Surfactants and Drugs: A Review. Appl. Biochem. Biotechnol..

[404] Macierzanka A., Torcello-Gómez A., Jungnickel C., Maldonado-Valderrama J. (2019). Bile salts in digestion and transport of lipids. Adv. Colloid Interface Sci..

[405] Pavlović N., Goločorbin-Kon S., Danić M., Stanimirov B., Al-Salami H., Stankov K., Mikov M. (2018). Bile acids and their derivatives as potential modifiers of drug release and pharmacokinetic profiles. Front. Pharmacol..

[406] Small D.M. (1968). Size and Structure of Bile Salt Micelles. Bile Salt Chemistry.

[407] Faustino C., Serafim C., Rijo P., Reis C.P. (2016). Bile acids and bile acid derivatives: Use in drug delivery systems and as therapeutic agents. Expert Opin. Drug Deliv..

[408] Hofmann A.F., Hagey L.R. (2008). Bile acids: Chemistry, pathochemistry, biology, pathobiology, and therapeutics. Cell. Mol. Life Sci..

[409] Holm R., Müllertz A., Mu H. (2013). Bile salts and their importance for drug absorption. Int. J. Pharm..

[410] Dai Y., Zhou R., Liu L., Lu Y., Qi J., Wu W. (2013). Liposomes containing bile salts as novel ocular delivery systems for tacrolimus (FK506): In vitro characterization and improved corneal permeation. Int. J. Nanomed..

[411] Hayakawa E., Chien D.S., Inagaki K., Yamamoto A., Wang W., Lee V.H.L. (1992). Conjunctival Penetration of Insulin and Peptide Drugs in the Albino Rabbit. Pharm. Res..

[412] Rojanasakul Y., Liaw J., Robinson J.R. (1990). Mechanisms of action of some penetration enhancers in the cornea: Laser scanning confocal microscopic and electrophysiology studies. Int. J. Pharm..

[413] Mahaling B., Katti D.S. (2016). Understanding the influence of surface properties of nanoparticles and penetration enhancers for improving bioavailability in eye tissues in vivo. Int. J. Pharm..

[414] Behl T., Kumar K., Brisc C., Rus M., Nistor-Cseppento D.C., Bustea C., Aron R.A.C., Pantis C., Zengin G., Sehgal A. (2021). Exploring the multifocal role of phytochemicals as immunomodulators. Biomed. Pharmacother..

[415] Nguyen L.T., Farcas A.C., Socaci S.A., Tofana M., Diaconeasa Z.M., Pop O.L., Salanta L.C. (2020). An Overview of Saponins—A Bioactive Group. Bull. UASVM Food Sci. Technol..

[416] Wang X., Ma Y., Xu Q., Shikov A.N., Pozharitskaya O.N., Flisyuk E.V., Liu M., Li H., Vargas-Murga L., Duez P. (2023). Flavonoids and saponins: What have we got or missed?. Phytomedicine.

[417] Guclu-Ustundag Ö., Mazza G. (2007). Saponins: Properties, applications and processing. Crit. Rev. Food Sci. Nutr..

[418] Vincken J.P., Heng L., de Groot A., Gruppen H. (2007). Saponins, classification and occurrence in the plant kingdom. Phytochemistry.

[419] Chen K., Wang N., Zhang X., Wang M., Liu Y., Shi Y. (2023). Potentials of saponins-based adjuvants for nasal vaccines. Front. Immunol..

[420] Grizzle W.E. (1991). Systemic absorption of insulin delivered topically to the rat eye. Investig. Ophthalmol. Vis. Sci..

[421] Pillion D.J., Recchia J., Wang P., Marcianit D.J., Kensils C.R. (1995). DS-1, a Modified Quillaja Saponin, Enhances Ocular and Nasal Absorption of Insulin. J. Pharm. Sci..

[422] Recchia J., Lurantos M.H.A., Amsden J.A., Storey J., Kensil C.R. (1995). A Semisynthetic Quillaja Saponin as a Drug Delivery Agent for Aminoglycoside Antibiotics. Pharm. Res..

[423] Sasaki H., Igarashi Y., Nagano T., Nishida K., Nakamura J. (1995). Different Effects of Absorption Promoters on Corneal and Conjunctival Penetration of Ophthalmic Beta-Blockers. Pharm. Res..

[424] Lu P., Wang R., Xing Y., Gao Y., Zhang Q., Xing B., Zhang Y., Yu C., Cai X., Shang Q. (2021). Development and evaluation of Panax notoginseng saponins contained in an in situ pH-Triggered gelling system for sustained ocular posterior segment drug delivery. Acupunct. Herb. Med..

[425] Sasaki H., Yamamura K., Tei C., Nishida K., Nakamura J. (1995). Ocular Permeability of FITC-Dextran with Absorption Promoter for Ocular Delivery of Peptide Drug. J. Drug Target..

[426] Sasaki H., Yamamura K., Mukai T., Nishida K., Nakamura J., Nakashima M., Ichikawa M. (2000). Modification of Ocular Permeability of Peptide Drugs by Absorption Promoters. Biol. Pharm. Bull..

[427] Li M., Lan J., Li X., Xin M., Wang H., Zhang F., Lu X., Zhuang Z., Wu X. (2019). Novel ultra-small micelles based on ginsenoside Rb1: A potential nanoplatform for ocular drug delivery. Drug Deliv..

[428] Sudji I.R., Subburaj Y., Frenkel N., García-Sáez A.J., Wink M. (2015). Membrane disintegration caused by the steroid saponin digitonin is related to the presence of cholesterol. Molecules.

[429] Wolosin J.M. (1988). Membrane Biology Regeneration of Resistance and Ion Transport in Rabbit Corneal Epithelium after Induced Surface Cell Exfoliation. J. Membr. Biol..

[430] Zuurendonk P.F., Tager J.M. (1974). Rapid separation of particulate components and soluble cytoplasm of isolated rat-liver cells. Biochim. Biophys. Acta.

[431] Dubinsky W.P., Cockrell R.S. (1975). Ca^2+^ transport across plasma and mitochondrial membranes of isolated hepatocytes. FEBS Lett..

[432] Siess E.A., Wieland O.H. (1976). Phosphorylation State of Cytosolic and Mitochondrial Adenine Nucleotides and of Pyruvate Dehydrogenase in Isolated Rat Liver Cells. Biochem. J..

[433] Murphy E., Coll K., Rich T.L., Williamson J.R. (1980). Hormonal effects on calcium homeostasis in isolated hepatocytes. J. Biol. Chem..

[434] Akiyama T., Takagi S., Sankawa U., Inari S., Saito H. (1980). Saponin-Cholesterol Interaction in the Multibilayers of Egg Yolk Lecithin As Studied by Deuterium Nuclear Magnetic Resonance: Digitonin and Its Analogues. Biochemistry.

[435] Frenkel N., Makky A., Sudji I.R., Wink M., Tanaka M. (2014). Mechanistic investigation of interactions between steroidal saponin digitonin and cell membrane models. J. Phys. Chem. B.

[436] Liaw J., Robinson J.R. (1992). The effect of polyethylene glycol molecular weight on corneal transport and the related influence of penetration enhancers. Int. J. Pharm..

[437] Thiel M.A., Coster D.J., Standfield S.D., Brereton H.M., Mavrangelos C., Zola H., Taylor S. (2002). Penetration of engineered antibody fragments into the eye. Clin. Exp. Immunol..

[438] Gallelli L., Cione E., Wang T., Zhang L. (2021). Glucocorticoid-like activity of escin: A new mechanism for an old drug. Drug Des. Devel. Ther..

[439] Wang H., Zhang L., Jiang N., Wang Z., Chong Y., Fu F. (2013). Anti-inflammatory effects of escin are correlated with the glucocorticoid receptor/NF-κB signaling pathway, but not the COX/PGF2α signaling pathway. Exp. Ther. Med..

[440] Zhao S.Q., Xu S.Q., Cheng J., Cao X.L., Zhang Y., Zhou W.P., Huang Y.J., Wang J., Hu X.M. (2018). Anti-inflammatory effect of external use of escin on cutaneous inflammation: Possible involvement of glucocorticoids receptor. Chin. J. Nat. Med..

[441] Gallelli L. (2019). Escin: A review of its anti-edematous, antiinflammatory, and venotonic properties. Drug Des. Devel. Ther..

[442] Wang K., Jiang Y., Wang W., Ma J., Chen M. (2015). Escin activates AKT-Nrf2 signaling to protect retinal pigment epithelium cells from oxidative stress. Biochem. Biophys. Res. Commun..

[443] Zhang F., Man X., Yu H., Liu L., Li Y. (2015). Synergistic protective effects of escin and low dose glucocorticoids against vascular endothelial growth factor induced blood retinal barrier breakdown in retinal pigment epithelial and umbilical vein endothelial cells. Mol. Med. Rep..

[444] Zhang F., Li Y., Zhang L., Mu G. (2013). Synergistic protective effects of escin and low-dose glucocorticoids on blood-retinal barrier breakdown in a rat model of retinal ischemia. Mol. Med. Rep..

[445] Burgalassi S., Monti D., Brignoccoli A., Fabiani O., Lenzi C., Pirone A., Chetoni P. (2004). Development of Cultured Rabbit Corneal Epithelium for Drug Permeation Studies: A Comparison with Excised Rabbit Cornea. J. Ocul. Pharmacol. Ther..

[446] Lane M.E. (2013). Skin penetration enhancers. Int. J. Pharm..

[447] Williams A.C., Barry B.W. (2004). Penetration enhancers. Adv. Drug Deliv. Rev..

[448] Narasimha Murthy S., Shivakumar H.N. (2010). Topical and Transdermal Drug Delivery. Handbook of Non-Invasive Drug Delivery Systems.

[449] Beastall J., Washington C. (1988). Mechanism of action of Azone as a percutaneous penetration enhancer: Lipid bilayer fluidity and transition temperature effects. Int. J. Pharm..

[450] Trommer H., Neubert R.H.H. (2006). Overcoming the stratum corneum: The modulation of skin penetration. A review. Ski. Pharmacol. Physiol..

[451] Abrego G., Alvarado H., Souto E.B., Guevara B., Bellowa L.H., Parra A., Calpena A., Garcia M.L. (2015). Biopharmaceutical profile of pranoprofen-loaded PLGA nanoparticles containing hydrogels for ocular administration. Eur. J. Pharm. Biopharm..

[452] Tang-Liu D.D.-S., Richman J.B., Weinkam R.J., Takruri H. (1994). Effects of Four Penetration Enhancers on Corneal Permeability of Drugs in Vitro. J. Pharm. Sci..

[453] Newton C., Gebhardt B.M., Kaufman H.E. (1988). Topically Applied Cyclosporine in Azone Prolongs Corneal Allograft Survival. Investig. Ophthalmol. Vis. Sci..

[454] Ismail M.I., Chen C.-C., Richman J.B., Andersen J.S., Tang-Liu D.D.-S. (1992). Comparison of Azone and Hexamethylene Lauramide in Toxicologic Effects and Penetration Enhancement of Cimetidine in Rabbit Eyes. Pharm. Res..

[455] Mao X., Zhang S., Hen H., Du L., Li G., Li B., Zhang H. (2010). Corneal permeability assay of topical eye drop solutions in rabbits by MRI. J. Huazhong Univ. Sci. Technol. Med. Sci..

[456] Tang-Liu D.D.-S., Burke P.J. (1988). The effect of azone on ocular levobunolol absorption: Calculating the area under the curve and its standard error using tissue sampling compartments. Pharm. Res..

[457] Afouna M.I., Hussein A.K., Ahmed O.A. (2014). Influence of the Interplay between AzoneTM as Permeation Enhancer and Carbopol-974^®^ as a Mucoadhesive upon the in vitro Transcorneal Release and the in vivo Antiglaucoma Effect of S-Timolol Maleate Ophthalmic Gel Formulations. Int. J. PharmTech Res..

[458] Afouna M.I., Roshdy H.R., Ibrahim H.M., Naim A.B., El-Marzoqi A. (2016). Maximization of the in vitro transcorneal release and the in vivo IOP-lowering effects of Latanoprost ophthalmic gel formulations using Azone as a penetration enhancer and Carbopol-974^®^ as a mucoadhesive. Drug Dev. Ind. Pharm..

[459] Milletti F. (2012). Cell-penetrating peptides: Classes, origin, and current landscape. Drug Discov. Today.

[460] Heitz F., Morris M.C., Divita G. (2009). Twenty years of cell-penetrating peptides: From molecular mechanisms to therapeutics. Br. J. Pharmacol..

[461] Jiang K., Gao X., Shen Q., Zhan C., Zhang Y., Xie C., Wei G., Lu W. (2017). Discerning the composition of penetratin for safe penetration from cornea to retina. Acta Biomater..

[462] Brasseur R., Divita G. (2010). Happy birthday cell penetrating peptides: Already 20years. Biochim. Biophys. Acta Biomembr..

[463] Jallouk A.P., Palekar R.U., Pan H., Schlesinger P.H., Wickline S.A. (2015). Modifications of Natural Peptides for Nanoparticle and Drug Design. Adv. Protein Chem. Struct. Biol..

[464] Liu C., Tai L., Zhang W., Wei G., Pan W., Lu W. (2014). Penetratin, a potentially powerful absorption enhancer for noninvasive intraocular drug delivery. Mol. Pharm..

[465] Morofuji R., Enomoto H., Honda T., Oyama Y., Ishida R., Kudo K., Okabe K. (2023). Exploring Cell-Penetrating Peptides as Penetration Enhancers in Eye Drop Formulations Using a Reconstructed Human Corneal Epithelial Model. Biol. Pharm. Bull..

[466] Morofuji R., Kudo K., Honda T., Kinugasa S., Matsuo T., Okabe K. (2024). Enhancing Corneal Drug Penetration Using Penetratin for Ophthalmic Suspensions. Biol. Pharm. Bull..

[467] Liu C., Jiang K., Tai L., Liu Y., Wei G., Lu W., Pan W. (2016). Facile Noninvasive Retinal Gene Delivery Enabled by Penetratin. ACS Appl. Mater. Interfaces.

[468] Yang X., Wang L., Li L., Han M., Tang S., Wang T., Han J., He X., He X., Wang A. (2019). A novel dendrimer-based complex co-modified with cyclic RGD hexapeptide and penetratin for noninvasive targeting and penetration of the ocular posterior segment. Drug Deliv..

[469] Jiang K., Chen J., Tai L., Liu C., Chen X., Wei G., Lu W., Pan W. (2020). Inhibition of post-trabeculectomy fibrosis via topically instilled antisense oligonucleotide complexes co-loaded with fluorouracil. Acta Pharm. Sin. B.

[470] Ge Y., Zhang A., Sun R., Xu J., Yin T., He H., Gou J., Kong J., Zhang Y., Tang X. (2020). Penetratin-modified lutein nanoemulsion in-situ gel for the treatment of age-related macular degeneration. Expert Opin. Drug Deliv..

[471] Gao X., Fan X., Jiang K., Hu Y., Liu Y., Lu W., Wei G. (2023). Intraocular siRNA Delivery Mediated by Penetratin Derivative to Silence Orthotopic Retinoblastoma Gene. Pharmaceutics.

[472] Thareja A., Leigh T., Hakkarainen J.J., Hughes H., Alvarez-Lorenzo C., Fernandez-Trillo F., Blanch R.J., Ahmed Z. (2024). Improving corneal permeability of dexamethasone using penetration enhancing agents: First step towards achieving topical drug delivery to the retina. Int. J. Pharm..

[473] Sun C., Zhang S., Xu N., Liu K., Wei F., Zhang X., Zhang J., Gao S., Yu Y., Ding X. (2024). Topical Ophthalmic Liposomes Dual-Modified with Penetratin and Hyaluronic Acid for the Noninvasive Treatment of Neovascular Age-Related Macular Degeneration. Int. J. Nanomed..

[474] Toffoletto N., Salema-Oom M., Nicoli S., Pescina S., González-Fernández F.M., Pinto C.A., Saraiva J.A., Alves de Matos A.P., Vivero-Lopez M., Huete-Toral F. (2024). Dexamethasone phosphate and penetratin co-eluting contact lenses: A strategy to enhance ocular drug permeability. Int. J. Pharm..

[475] Frankel A.D., Pabo C.O. (1988). Cellular Uptake of the Tat Protein from Human Immunodeficiency Virus. Cell.

[476] Green M., Loewenstein P.M. (1988). Autonomous Functional Domains of Chemically Synthesized Human Immunodeficiency Virus Tat Trans-Activator Protein. Cell.

[477] Zhang X., Li Y., Cheng Y., Tan H., Li Z., Qu Y., Mu G., Wang F. (2015). Tat PTD-endostatin: A novel anti-angiogenesis protein with ocular barrier permeability via eye-drops. Biochim. Biophys. Acta Gen. Subj..

[478] Mann D.A., Frankel A.D. (1991). Endocytosis and targeting of exogenous HIV-1 Tat protein. EMBO J..

[479] Fawell S., Seery J., Daikh Y., Moore C., Chen L.L., Pepinsky B., Barsoum J. (1994). Tat-mediated delivery of heterologous proteins into cells. Proc. Natl. Acad. Sci. USA.

[480] Kaplan I.M., Wadia J.S., Dowdy S.F. (2005). Cationic TAT peptide transduction domain enters cells by macropinocytosis. J. Control. Release.

[481] Richard J.P., Melikov K., Brooks H., Prevot P., Lebleu B., Chernomordik L.V. (2005). Cellular uptake of unconjugated TAT peptide involves clathrin-dependent endocytosis and heparan sulfate receptors. J. Biol. Chem..

[482] Wang Y., Lin H., Lin S., Qu J., Xiao J., Huang Y., Xiao Y., Fu X., Yang Y., Li X. (2010). Cell-penetrating peptide TAT-mediated delivery of acidic FGF to retina and protection against ischemia-reperfusion injury in rats. J. Cell. Mol. Med..

[483] Li Y., Li L., Li Z., Sheng J., Zhang X., Feng D., Zhang X., Yin F., Wang A., Wang F. (2016). Tat PTD-Endostatin-RGD: A novel protein with anti-angiogenesis effect in retina via eye drops. Biochim. Biophys. Acta Gen. Subj..

[484] Zhu M., Yang H., Chen Z., Xia X., Deng Q., Shen Y. (2020). A cell-permeable peptide inhibitor of p55PIK signaling alleviates ocular inflammation in mouse models of uveitis. Exp. Eye Res..

[485] Huang J., Zhang Y., Lin T., Yin H., Pan Y., Zhu M., Zhang M. (2023). A cell-permeable peptide inhibitor of p55PIK signaling alleviates suture-induced corneal neovascularization and inflammation. Heliyon.

[486] Rohira H., Shankar S., Yadav S., Shah S.G., Chugh A. (2021). Enhanced in vivo antifungal activity of novel cell penetrating peptide natamycin conjugate for efficient fungal keratitis management. Int. J. Pharm..

[487] Vasconcelos A., Vega E., Pérez Y., Gómara M.J., García M.L., Haro I. (2015). Conjugation of cell-penetrating peptides with poly(lactic-co-glycolic acid)-polyethylene glycol nanoparticles improves ocular drug delivery. Int. J. Nanomed..

[488] Gonzalez-Pizarro R., Parrotta G., Vera R., Sánchez-López E., Galindo R., Kjeldsen F., Badia J., Baldoma L., Espina M., García M.L. (2019). Ocular penetration of fluorometholone-loaded PEG-PLGA nanoparticles functionalized with cell-penetrating peptides. Nanomedicine.

[489] Chu Y., Chen N., Yu H., Mu H., He B., Hua H., Wang A., Sun K. (2017). Topical ocular delivery to laser-induced choroidal neovascularization by dual internalizing rgd and tat peptide-modified nanoparticles. Int. J. Nanomed..

[490] Suda K., Murakami T., Gotoh N., Fukuda R., Hashida Y., Hashida M., Tsujikawa A., Yoshimura N. (2017). High-density lipoprotein mutant eye drops for the treatment of posterior eye diseases. J. Control. Release.

[491] Yang H., Wu P., Wang T., Yu Y., Li J., Liu R., Ruan Q. (2024). Topical ophthalmic instillation of engineered hmscs-derived exosomes: A novel non-invasive therapeutic strategy for ocular posterior-segment disorder. Biochem. Biophys. Res. Commun..

[492] Lou Q., Pan L., Xiang S., Li Y., Jin J., Tan J., Huang B., Nan K., Lin S. (2023). Suppression of NLRP3/Caspase-1/GSDMD Mediated Corneal Epithelium Pyroptosis Using Melatonin-Loaded Liposomes to Inhibit Benzalkonium Chloride-Induced Dry Eye Disease. Int. J. Nanomed..

[493] Li Z., Yu H., Liu C., Wang C., Zeng X., Yan J., Sun Y. (2023). Efficiency co-delivery of ellagic acid and oxygen by a non-invasive liposome for ameliorating diabetic retinopathy. Int. J. Pharm..

[494] Shan S., Jia S., Lawson T., Yan L., Lin M., Liu Y. (2019). The use of TAT peptide-functionalized graphene as a highly nuclear-targeting carrier system for suppression of choroidal melanoma. Int. J. Mol. Sci..

[495] Wu B., Li M., Li K., Hong W., Lv Q., Li Y., Xie S., Han J., Tian B. (2021). Cell penetrating peptide TAT-functionalized liposomes for efficient ophthalmic delivery of flurbiprofen: Penetration and its underlying mechanism, retention, anti-inflammation and biocompatibility. Int. J. Pharm..

[496] Rothbard J.B., Kreider E., VanDeusen C.L., Wright L., Wylie B.L., Wender P.A. (2002). Arginine-rich molecular transporters for drug delivery: Role of backbone spacing in cellular uptake. J. Med. Chem..

[497] Wender P.A., Mitchell D.J., Pattabiraman K., Pelkey E.T., Steinman L., Rothbard J.B. (2000). The design, synthesis, and evaluation of molecules that enable or enhance cellular uptake: Peptoid molecular transporters. Proc. Natl. Acad. Sci. USA.

[498] Mitchell D.J., Kim D.T., Steinman L., Fathman C.G., Rothbard J.B. (2000). Polyarginine enters cells more efficiently than other polycationic homopolymers. J. Pept. Res..

[499] McEwan G.T., Jepson M.A., Hirst B.H., Simmons N.L. (1993). Polycation-induced enhancement of epithelial paracellular permeability is independent of tight junctional characteristics. Biochim. Biophys. Acta.

[500] Ohtake K., Maeno T., Ueda H., Natsume H., Morimoto Y. (2003). Poly-L-Arginine Predominantly Increases the Paracellular Permeability of Hydrophilic Macromolecules across Rabbit Nasal Epithelium In Vitro. Pharm. Res..

[501] Nemoto E., Takahashi H., Kobayashi D., Ueda H., Morimoto Y. (2006). Effects of Poly-L-arginine on the Permeation of Hydrophilic Compounds through Surface Ocular Tissues. Biol. Pharm. Bull..

[502] Liu C., Lan Q., He W., Nie C., Zhang C., Xu T., Jiang T., Wang S. (2017). Octa-arginine modified lipid emulsions as a potential ocular delivery system for disulfiram: A study of the corneal permeation, transcorneal mechanism and anti-cataract effect. Colloids Surf. B Biointerfaces.

[503] Nemoto E., Ueda H., Masayuki A., Hideshi N., Morimoto Y. (2007). Ability of Poly-L-arginine to Enhance Drug Absorption into Aqueous Humor and Vitreous Body after Instillation in Rabbits. Biol. Pharm. Bull..

[504] Morris M.C., Depollier J., Mery J., Heitz F., Divita G. (2001). A peptide carrier for the delivery of biologically active proteins into mammalian cells. Nat. Biotechnol..

[505] Pescina S., Sala M., Padula C., Scala M.C., Spensiero A., Belletti S., Gatti R., Novellino E., Campiglia P., Santi P. (2016). Design and synthesis of new cell penetrating peptides: Diffusion and distribution inside the cornea. Mol. Pharm..

[506] Henriques S.T., Quintas A., Bagatolli L.A., Homblé F., Castanho M.A.R.B. (2007). Energy-independent translocation of cell-penetrating peptides occurs without formation of pores. A biophysical study with pep-1. Mol. Membr. Biol..

[507] Henriques S.T., Castanho M.A.R.B. (2004). Consequences of nonlytic membrane perturbation to the translocation of the cell penetrating peptide pep-1 in lipidic vesicles. Biochemistry.

[508] Henriques S.T., Costa J., Castanho M.A.R.B. (2005). Translocation of β-galactosidase mediated by the cell-penetrating peptide pep-1 into lipid vesicles and human HeLa cells is driven by membrane electrostatic potential. Biochemistry.

[509] Weller K., Lauber S., Lerch M., Renaud A., Merkle H.P., Zerbe O. (2005). Biophysical and biological studies of end-group-modified derivatives of Pep-1. Biochemistry.

[510] Kim D.W., Lee S.H., Ku S.K., Lee J.E., Cha H.J., Youn J.K., Kwon H.Y., Park J.H., Park E.Y., Cho S.W. (2015). The effects of PEP-1-FK506BP on dry eye disease in a rat model. BMB Rep..

[511] Kim D.W., Lee S.H., Shin M.J., Kim K., Ku S.K., Youn J.K., Bin Cho S., Park J.H., Lee C.H., Son O. (2015). PEP-1-FK506BP inhibits alkali burn-induced corneal inflammation on the rat model of corneal alkali injury. BMB Rep..

[512] Carter S.B. (1967). Effects of Cytochalasins on Mammalian Cells. Nature.

[513] Ito S., Sato E., Loewenstein W.R. (1974). Studies on the Formation of a Permeable Cell Membrane Junction I. Coupling under Various Conditions of Membrane Contact. Effects of Colchicine, Cytochalasin B, Dinitrophenol. J. Membr. Biol..

[514] Stevenson B.R., Begg D.A. (1994). Concentration-dependent effects of cytochalasin d on tight junctions and actin filaments in mdck epithelial cells. J. Cell Sci..

[515] Maclean-Fletcher S., Pollard T.D. (1980). Mechanism of Action of Cytochalasin B on Actin. Cell.

[516] Lin D.C., Tobin K.D., Grumet M., Lin S. (1980). Cytochalasins Inhibit Nuclei-Induced Actin Polymerization by Blocking Filament Elongation. J. Cell Biol..

[517] Brown S.S., Spudich J.A. (1979). Cytochalasin inhibits the rate of elongation of actin filament fragments. J. Cell Biol..

[518] Cereijido M., Meza I., Martinez-Palomo A. (1981). Occluding junctions in cultured epithelial monolayers. Am. J. Physiol..

[519] Meza I., Ibarra G., Sabanero M., Martinez-Palomo A., Cereijido M. (1980). Occluding Junctions and Cytoskeletal Components in a Cultured Transporting Epithelium. J. Cell Biol..

[520] Fletcher D.A., Mullins R.D. (2010). Cell mechanics and the cytoskeleton. Nature.

[521] Wickstead B., Gull K. (2011). The evolution of the cytoskeleton. J. Cell Biol..

[522] Lai W.F., Wong W.T. (2020). Roles of the actin cytoskeleton in aging and age-associated diseases. Ageing Res. Rev..

[523] Rojanasakul Y., Robinson J.R. (1991). The cytoskeleton of the cornea and its role in tight junction permeability. Int. J. Pharm..

[524] Lee V.H.L., Carson L.W., Takemoto K.A. (1986). Macromolecular drug absorption in the albino rabbit eye. Int. J. Pharm..

[525] Liaw J., Chang S.F., Hsiao F.C. (2001). In vivo gene delivery into ocular tissues by eye drops of poly(ethylene oxide)-poly(propylene oxide)-poly(ethylene oxide) (PEO-PPO-PEO) polymeric micelles. Gene Ther..

[526] Wang C.J., Huang Q.W., Qi H.Y., Guo P., Huang S.X. (2005). Promoting effect of borneol on the permeability of puerarin eye drops and timolol maleate eye drops through the cornea in vitro. Pharmazie.

[527] Qi H.P., Gao X.C., Zhang L.Q., Wei S.Q., Bi S., Yang Z.C., Cui H. (2013). in vitro evaluation of enhancing effect of borneol on transcorneal permeation of compounds with different hydrophilicities and molecular sizes. Eur. J. Pharmacol..

[528] Song J., Bi H., Xie X., Guo J., Wang X., Liu D. (2013). Natural borneol enhances geniposide ophthalmic absorption in rabbits. Int. J. Pharm..

[529] Yang H., Xun Y., Li Z., Hang T., Zhang X., Cui H. (2009). Influence of Borneol on In Vitro Corneal Permeability and on In Vivo and In Vitro Corneal Toxicity. J. Ocul. Pharmacol. Ther..

[530] Mao Z., Wang X., Liu Y., Huang Y., Liu Y., Di X. (2017). Simultaneous determination of seven alkaloids from rhizoma corydalis decumbentis in rabbit aqueous humor by LC–MS/MS: Application to ocular pharmacokinetic studies. J. Chromatogr. B.

[531] Xu X., Yu N., Bai Z., Xun Y., Jin D., Li Z., Cui H. (2011). Effect of menthol on ocular drug delivery. Graefes Arch. Clin. Exp. Ophthalmol..

[532] Ahn S., Eom Y., Kang B., Park J., Lee H.K., Kim H.M., Song J.S. (2018). Effects of Menthol-Containing Artificial Tears on Tear Stimulation and Ocular Surface Integrity in Normal and Dry Eye Rat Models. Curr. Eye Res..

[533] Bai J.H., Ding X.M., Mou H.Y., Wang S.L., Chen S.H. (2022). Menthol in Combination with Iontophoresis Promotes Natamycin Penetration through the Cornea: In Vitro and In Vivo Studies. Bull. Exp. Biol. Med..

[534] Gelfuso G.M., Ferreira-Nunes R., Dalmolin L.F., Ré A.C.S., Santos G.A., Sá F.A.P., Cunha-Filho M., Alonso A., Neto S.A.M., Anjos J.L.V. (2020). Iontophoresis enhances voriconazole antifungal potency and corneal penetration. Int. J. Pharm..

[535] Rojekar S., Parit S., Gholap A.D., Manchare A., Nangare S.N., Hatvate N., Sugandhi V.V., Paudel K.R., Ingle R.G. (2024). Revolutionizing Eye Care: Exploring the Potential of Microneedle Drug Delivery. Pharmaceutics.

[536] Haley R.M., Gottardi R., Langer R., Mitchell M.J. (2020). Cyclodextrins in drug delivery: Applications in gene and combination therapy. Drug Deliv. Transl. Res..

[537] Alam F., Elsherif M., Alqattan B., Salih A., Lee S.M., Yetisen A.K., Park S., Butt H. (2021). 3D Printed Contact Lenses. ACS Biomater. Sci. Eng..

[538] Kashkooli H.H., Farokh A., Mohammadi S., Marcotulli M., Franco S., Angelini R., Ruocco G., Khalili H., Cidonio G. (2025). Localised Therapies Using 3D-Printed Collagen-Based Micro-Implant for Ocular Indications. Macromol. Mater. Eng..

[539] Salahuddin A., Ashraf A., Ahmad K., Hou H. (2024). Recent advances in chitosan-based smart hydrogel for drug delivery systems. Int. J. Biol. Macromol..

[540] Kalyanwat R., Shrivastava B., Pathak K. (2016). Preparation and Evaluation of Bioadhesive Ocular Inserts of Aceclofenac. Int. J. Pharm. Sci. Rev. Res..

[541] Ioannou N., Luo J., Qin M., Di Luca M., Mathew E., Tagalakis A.D., Lamprou D.A., Yu-Wai-Man C. (2023). 3D-printed long-acting 5-fluorouracil implant to prevent conjunctival fibrosis in glaucoma. J. Pharm. Pharmacol..

[542] Abrigo N.A., Dods K.K., Makovsky C.A., Lohan S., Mitra K., Newcomb K.M., Le A., Hartman M.C.T. (2023). Development of a Cyclic, Cell Penetrating Peptide Compatible with In Vitro Selection Strategies. ACS Chem. Biol..

[543] Najjar K., Erazo-Oliveras A., Brock D.J., Wang T.Y., Pellois J.P. (2017). An L- to D-amino acid conversion in an endosomolytic analog of the cell-penetrating peptide TAT influences proteolytic stability, endocytic uptake, and endosomal escape. J. Biol. Chem..

